# Berberine-Functionalized Bismuth-Doped Carbon Dots in a Pathogen-Responsive Hydrogel System: A Multifaceted Approach to Combating Periodontal Diseases

**DOI:** 10.1021/acsnano.5c00561

**Published:** 2025-05-02

**Authors:** Xuan Li, Regina Huang, Pugeng Li, Fung Kit Tang, Jing He, Hanyu Sun, Xiaoyu Wang, Miao Wang, Xinmiao Lan, Xinna Wang, Sarah Sze Wah Wong, Lijian Jin, Ken Cham-Fai Leung, Hai Ming Wong, Sheng Wang, Lanping Guo, Pei-Hui Ding, Xiaolin Yu

**Affiliations:** † Faculty of Dentistry, The University of Hong Kong, Hong Kong SAR 999077, PR China; ‡ Hospital of Stomatology, Guanghua School of Stomatology, Guangdong Provincial Key Laboratory of Stomatology, Sun Yat-sen University, Guangzhou 510055, PR China; § Beijing Area Major Laboratory of Peptide and Small Molecular Drugs, Engineering Research Centre of Ministry of Education of China, Beijing Laboratory of Biomedical Materials, School of Pharmaceutical Science, Capital Medical University, Beijing 100069, PR China; ∥ Department of Mechanical Engineering, The University of Hong Kong, Hong Kong SAR 999077, PR China; ⊥ Immunology of Fungal Infections Unit, Institut Pasteur, Paris 75015, France; # Department of Chemistry, The Hong Kong Baptist University, Kowloon, Hong Kong SAR 999077, PR China; ∇ State Key Laboratory for Quality Ensurance and Sustainable Use of Dao-di Herbs, National Resource Center for Chinese Materia Medica, China Academy of Chinese Medical Sciences, Beijing 100700, PR China; ○ Stomatology Hospital, School of Stomatology, Zhejiang University School of Medicine, Hangzhou 310006, PR China

**Keywords:** pathogen-responsive hydrogel, periodontitis, immune-responses modulation, alveolar bone loss, subgingival microbiota

## Abstract

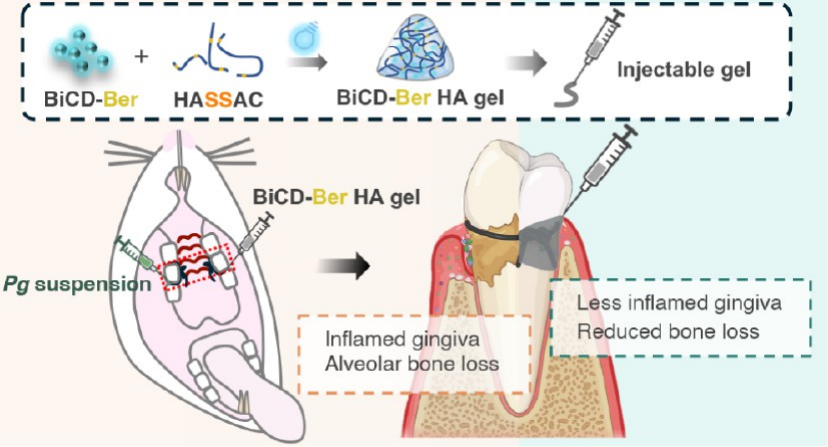

Periodontal disease, a global health burden linked to dysbiotic oral polymicrobial communities and disrupted immune-inflammatory responses, is critically mediated by*Porphyromonas gingivalis*(*Pg*)—the keystone pathogen that sabotages host immunity, triggers tissue inflammation and destruction, and disrupts microbiota balance. Effective therapies should combine antimicrobial action, immune modulation, virulence suppression, and microbiome restoration. Bismuth ions and berberine, which exhibit antimicrobial and epithelial barrier-protecting effects, show potential effectiveness in treating periodontal diseases but face practical limitations due to poor water solubility and bioavailability. To address this, we developed bismuth-doped carbon dots functionalized with structure-modified berberine (BiCD-Ber) as a multifunctional nanomedicine. BiCD-Ber eradicated *Pg* in various forms, restored *Pg*-perturbed immune responses in gingival fibroblasts, and preserved epithelial barrier integrity. The doped bismuth ions neutralized *Pg* virulence factors by blocking the catalytic sites of gingipains. To facilitate *in vivo* delivery, BiCD-Ber was encapsulated in a disulfide-modified hyaluronic acid hydrogel that degrades in response to *Pg* metabolites. This BiCD-Ber hydrogel system modulated subgingival microbiota, alleviated inflammation in gingiva, and thereby prevented alveolar bone loss. This approach to concurrently eliminating *Pg*, modulating inflammatory responses , suppressing virulence factors, and restoring microbiota showcases great potential in managing periodontitis effectively.

## Introduction

Periodontal disease, also known as gum disease, affects a significant portion of the global population and imposes a substantial socioeconomic burden worldwide.^1^ This chronic inflammatory condition impacts the tooth-supporting tissues, leading to gum inflammation, bone loss, and tooth mobility. Currently, severe periodontitis remains the major cause of tooth loss in adults.^2^ Increasing evidence has revealed that periodontal disease shares risk factors with noncommunicable diseases (NCDs) and conditions, such as cardiovascular disease and diabetes.^3^ Hence, effective periodontal treatments have been demonstrated to bring positive outcomes for associated systemic comorbidities,^4−6^ highlighting the importance of periodontal care for overall health and well-being.

It is widely recognized that periodontitis is linked to the dysbiotic oral microbiota and disrupted immuno-inflammatory responses.^7^*Porphyromonas gingivalis*(*Pg*), as the keystone pathogen, drives disease progression by manipulating host immunity, disrupting microbial symbiosis, and expediting tissue destruction through virulence factors like gingipains.^8^ Current antibiofilm therapeutics, such as antibiotics and antiseptic mouthwash, may bring other health pitfalls, such as antibiotic resistance and dysbiosis of the oral microbiota, exacerbating oral or general health issues. Moreover, our group previously reported that *Pg* persisters could survive the metronidazole (MTZ) treatment at lethal concentrations, and those MTZ-tolerant *Pg* cells could recover their population after the growth environment is restored.^9^ Of note, our findings also indicated that MTZ-treated *Pg* maintained its ability to invade host cells and perturb host immuno-inflammatory responses, as the common antibiotic treatment can only reduce the *Pg* cells’ viability but may not be able to deactivate the virulence factors of *Pg*.^10^ These findings underline the limitations of conventional antibiotics and highlight the urgent need for antibiotic-free strategies that prioritize precision, efficacy, and sustainability in periodontal therapy. As such, emerging approaches, such as nanobased antimicrobials,^11^ photodynamic therapy,^12^ and host immune modulation therapy,^13^ targeting pathogen viability and virulence without disrupting commensal microbiota, have attracted significant attention in advanced periodontal treatments. These antibiotic-free approaches can effectively deliver therapeutic agents to penetrate and disrupt pathogenic biofilms while reducing side effects via localized treatments with limited systemic toxicity. It is noteworthy that these strategies not only mitigate the crisis of antibiotic resistance but also align with the multifactorial pathogenesis of periodontitis, offering synergistic solutions to manage periodontal diseases by tackling *Pg* and counteracting its negative effects on the host innate immunity and oral microbial community.

Bismuth-based therapeutics have emerged as a promising antibiotic-free approach to combating microbes due to their exceptional biosafety and antimicrobial efficacy. For example, bismuth-based reagents containing Bi^3+^ have been applied in combination with antibiotics to treat*Helicobacter pylori*infections for a long time.^14^ A recent study reports that bismuth drugs could function as antiviral agents with great potential to fight against severe acute respiratory syndrome coronavirus 2 (SARS-CoV-2).^15^ Advancements in nanotechnology have further unlocked the potential of bismuth, with engineered nanoparticles disrupting bacterial DNA and metabolic pathways to combat antibiotic-resistant strains.^16−18^ The functionalization of bismuth nanoparticles with metallic or organic components enhances their antimicrobial potency while conferring adjunctive capabilities such as photothermal and photodynamic activities.^19−21^ Nowadays, there is an increasing trend of applying bismuth-based drugs or nanomedicines for oral healthcare owing to their exceptional antimicrobial and antibiofilm capacity. Our group has pioneered the development of bismuth reagents/materials to specifically explore their potential applications for oral healthcare. Importantly, our studies have found that bismuth ions exhibit potent antibacterial effects against the planktonic, biofilm, and intracellular forms of *Pg*, while restoring the *Pg*-sabotaged immune responses.^22^ Meanwhile, our results show that bismuth drugs, together with MTZ, could completely eradicate *Pg* cells, including the persisters, through a synergistic effect.^23^ Recently, we further developed a rapid synthetic approach to constructing bismuth metal–organic frameworks as bismuth ion reservoirs from micro- to nanoscale to selectively inhibit the growth of periodontal pathogens, highlighting the potentials of bismuth-based reagents/materials in restoring the dysbiotic oral microbiota via specifically suppressing periodontal pathogens.^24^ However, bismuth-based monotherapy or antibiotic combinations could eradicate the pathogens and persister cells, but they fail to address the multifactorial nature of periodontitis—particularly the protection of gingival epithelial integrity. To bridge this gap, we integrate bismuth ions with berberine, a phytochemical with complementary barrier-protective and anti-inflammatory properties, into a nanoparticle-based platform for more comprehensive periodontal therapy.

Berberine, a plant-extracted alkaloid, has been used in traditional Chinese medicine for a long time. This phytochemical compound exists in many herbal medicines, which are traditionally utilized as antimicrobial and antidiarrheal agents.^25^ Modern research has uncovered various pharmacological properties and medical applications of berberine, including anticancer, immune-modulation, and antidiabetic activities, thereby highlighting its potential as a multitargeted medicinal remedy.^26,27^ Its quaternary ammonium isoquinoline structure enables bacterial membranes disruption and interference with proteins and deoxyribonucleic acid (DNA).^28,29^ In addition, it is capable of protecting and repairing intestinal barrier function by reducing pathogen adherence, maintaining epithelial tight junctions, attenuating inflammation, and modulating the gut microbiome.^30−33^ Similar to the gastrointestinal tract, the oral cavity also accommodates unique microbiomes, while dysbiotic microbiome and a dysfunctional oral mucosa barrier lead to different oral diseases. As a result, berberine may also be a good candidate for treating oral diseases. Indeed, berberine exhibits attractive therapeutic effects on oral conditions like periodontal disease, by inhibiting the growth of periodontal pathogens, promoting osteogenic differentiation, and controlling inflammation at periodontal compartments.^34−36^ However, its clinical translation is hampered by poor solubility and bioavailability, necessitating advanced delivery systems, such as nanoparticle carriers. Moreover, previous studies have explored either antimicrobial or anti-inflammatory effects of berberine, and its combination with other therapeutic agents, such as bismuth ions, in nanoparticle forms remains unexplored. This integration can offer unique advantages, including enhancing the antimicrobial effect, overcoming intrinsic drug limitations, and introducing multifaceted therapeutic strategies for managing periodontal disease.

Carbon dots (CDs), with sizes ranging from 1 to 10 nm, have emerged as versatile nanomaterials with broad applications in bioimaging, drug delivery, biosensing, and theranostics due to their unique optical properties, excellent biocompatibility, and tunable surface functionalities.^37^ Their ability to selectively target specific cells or tissues, along with their low toxicity and high stability, makes them attractive candidates as nanomedicines for treating various diseases.^38^ Our group has successfully constructed red-emissive CDs conjugated with amphotericin B to fortify oral epithelial tissue against invasive fungal infections, demonstrating the theranostic potential of CDs in oral healthcare.^39^ Moreover, the properties of CDs can be fine-tuned based on specific biomedical applications, such as improving imaging contrast, enhancing cellular uptake, controlling the release of therapeutic agents, or boosting antimicrobial effects, by doping metal ions during the synthetic process.^40^ This doping strategy opens up different possibilities for developing advanced CD-based nanomaterials with enhanced performance and tailored functionalities for various biomedical applications.

Recently, injectable hydrogel systems have made significant advancements owing to their flexible properties that enable localized delivery in a convenient and minimally invasive mode. These hydrogel systems offer various benefits, including site-specific targeting, sustained release of therapeutic agents, and enhanced tissue regeneration.^41^ Notably, smart hydrogels, particularly stimuli-responsive hydrogel systems designed to react to physical, chemical, or even biological stimuli, are effective in delivering active compounds or therapeutic agents in a controlled-release manner, making them well-suited for treating different diseases or conditions.^42^ In particular, the pathogen-responsive hydrogel systems can release the antimicrobial agents on demand, thereby improving drug stability, reducing dosing frequency, and minimizing systemic toxicity. Taken together, combining CDs with a hydrogel delivery system offers a synergistic approach to address the complex challenges of managing oral diseases, such as periodontal disease, through targeted delivery of antimicrobial agents and controlled inflammatory responses for improved treatment effectiveness.

Recent advancements in antibiotic-free periodontal therapies have integrated state-of-the-art nanomaterials or biomaterials to address the multifactorial nature of periodontitis. Strategies such as developing highly efficient photosensitizers for pathogen eradication,^43^ introducing photothermal nanoenzyme systems for synergistic pathogen elimination,^44^ and constructing cationic polymer-based hydrogels for immunomodulation^45^ demonstrate progress in targeting specific aspects of the disease. However, effective periodontal treatment requires a triad of actions, including controlling pathogenic biofilms, modulating dysregulated host immunity, and restoring microbial symbiosis, while these objectives are rarely achieved concurrently by existing approaches.

**Scheme 1:**
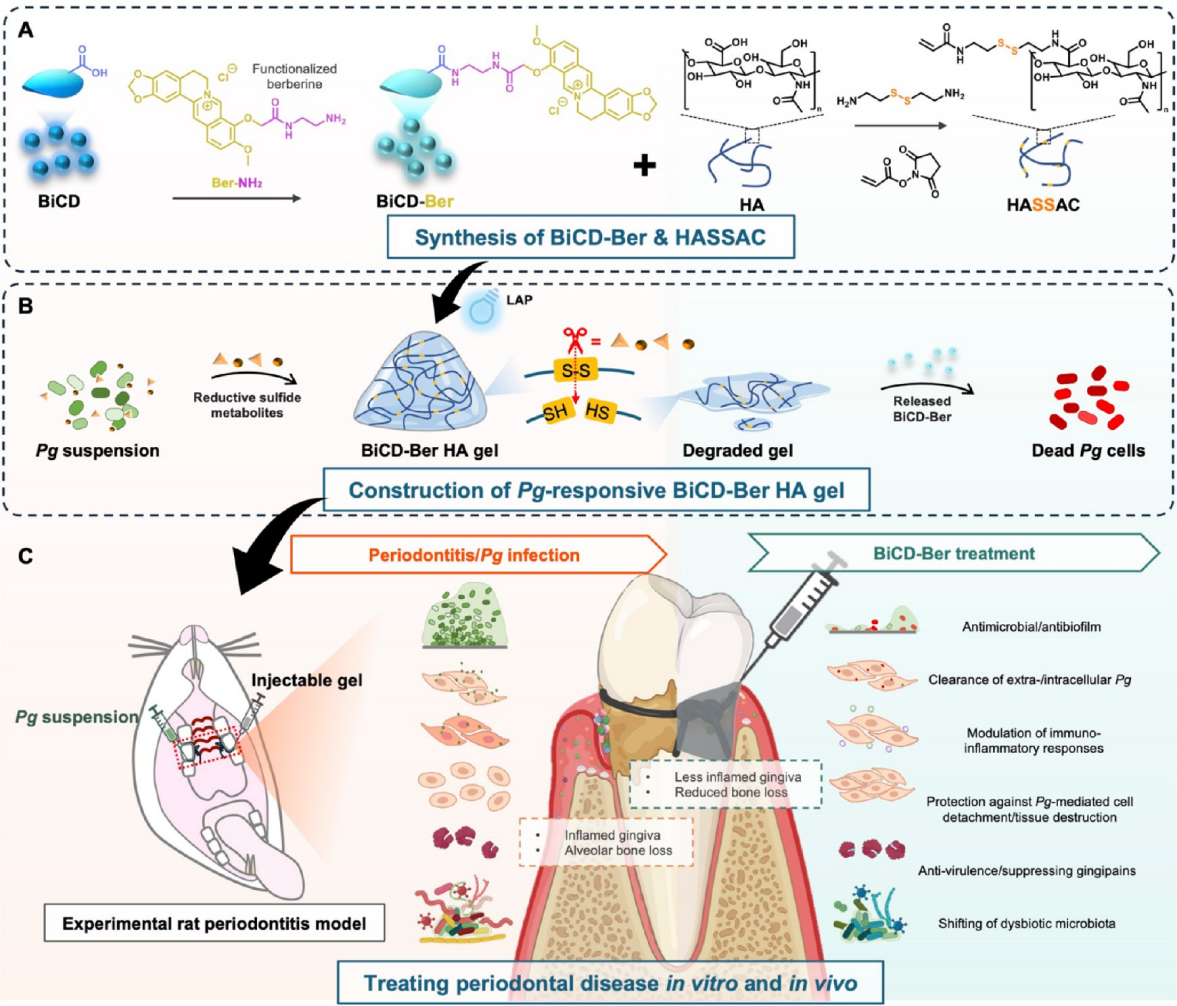
Schematic Illustration of (A) Synthesizing Bismuth-Doped Carbon Dots with Conjugation of Functionalized Berberine (BiCD-Ber) and Disulfide-Bond Modified Hyaluronic Acid to (B) Construct *Pg*-Responsive Hydrogel System with Encapsulation of BiCD-Ber; (C) This Pathogen-Responsive Injectable Hydrogel System Could Combat Periodontal Disease Both In Vitro and In Vivo Via Antimicrobial Activity, Modulation of Host Immuno-Inflammatory Responses, Anti-virulence, And Shifting of the Dysbiotic Subgingival Microbiota Part of the scheme was created in BioRender.

In this work, bismuth-doped CDs conjugated with berberine (BiCD-Ber) were developed as an antibiotic-free nanomedicine for combating *Pg in vitro*, and a *Pg* metabolites-responsive injectable hydrogel was engineered as a local delivery system to more effectively deliver BiCD-Ber *in vivo* (Scheme 1). Our *in vitro* studies demonstrated that BiCD-Ber could effectively eradicate *Pg* in different forms (i.e., planktonic and biofilm) and eliminate *Pg* inside and/or outside human gingival epithelial cells (HGECs) and primary human gingival fibroblasts (pHGF). Moreover, BiCD-Ber could reverse *Pg*-perturbed innate immune responses by restoring the levels of pro-inflammatory cytokines (e.g., IL-6 and IL-8) in IL-1β-stimulated pHGF. Importantly, the detachment of HGECs induced by *Pg* can also be significantly rescued by BiCD-Ber. These findings suggest that BiCD-Ber is capable of eradicating *Pg*, restoring *Pg*-sabotaged host immune responses, and protecting the epithelial barrier from being disrupted by *Pg*, possibly by blocking the active sites of *Pg*-produced protease gingipains . Our *in vivo* studies further showed that BiCD-Ber encapsulated in a *Pg*-responsive hyaluronic acid hydrogel delivery system could effectively prevent alveolar bone loss, alleviate inflammation in gingiva, and modulate subgingival microbiota. By incorporating antimicrobial, antivirulence, immunomodulatory, and microbiota-restorative functions, our study offers a comprehensive approach to periodontal treatment in an antibiotic-free manner. It may shed light on the development of a multifunctional therapeutic system as an effective strategy to manage and prevent the progression of periodontal disease, thereby benefiting both oral and general health.

## Results and Discussion

### Synthesis and Characterization of BiCD and BiCD-Ber

**Figure 1:**
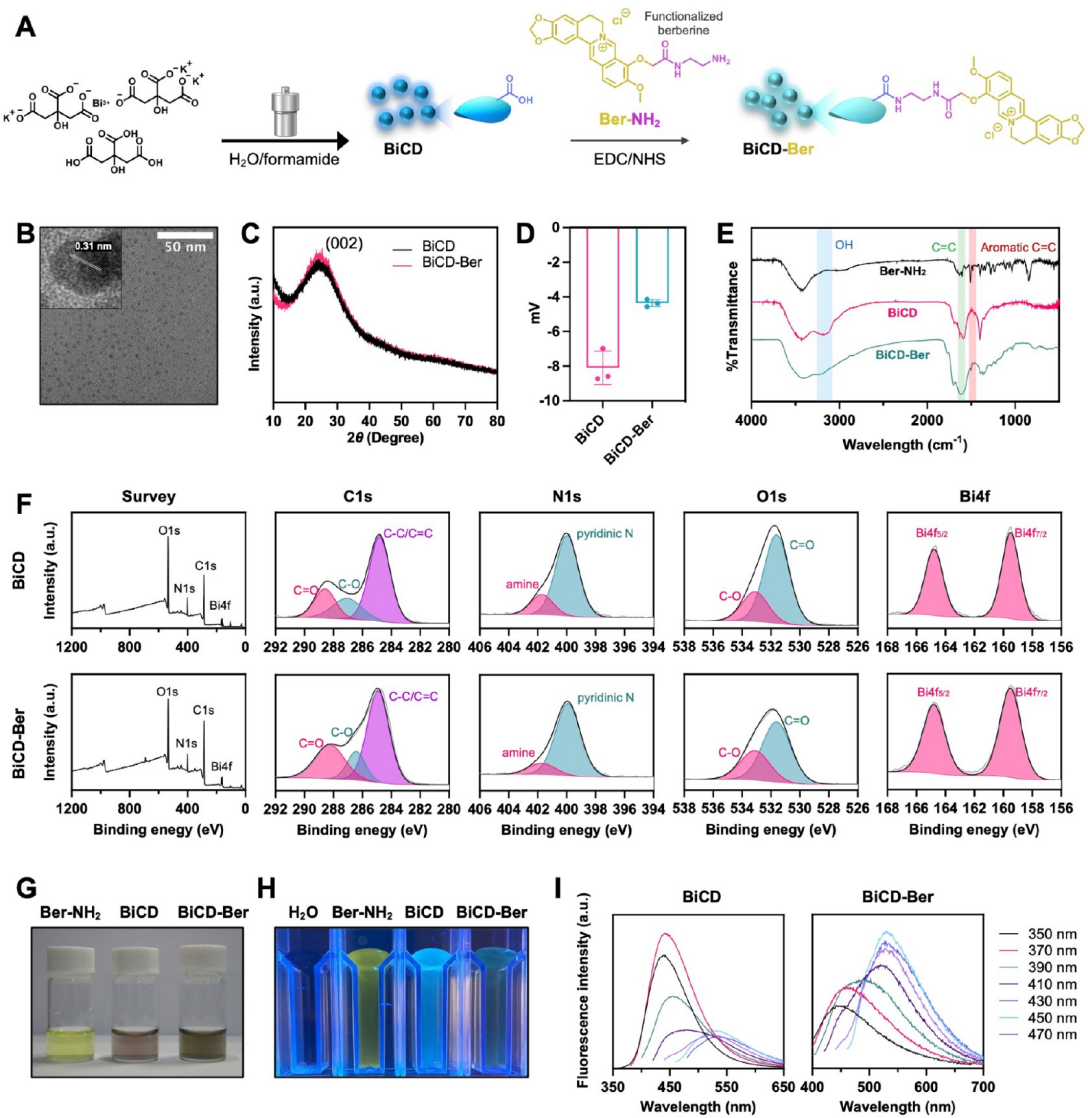
Synthesis and characterization of BiCD and BiCD-Ber. (A) Scheme of BiCD synthesis and the following conjugation with Ber-NH_2_ for BiCD-Ber. Part of the scheme was created in BioRender. (B) Transmission electron microscopy (TEM) image of BiCD with a lattice spacing of 0.31 nm. (C) X-ray diffraction (XRD) patterns of BiCD and BiCD-Ber with a wide angle (002) at approximately 25°. (D) ζ-potentials of BiCD and BiCD-Ber. (E) Fourier transform infrared (FTIR) spectra of BiCD, Ber-NH_2_, and BiCD-Ber. (F) X-ray photoelectron spectroscopy (XPS) analyses of BiCD and BiCD-Ber. Representative (G) optical and (H) fluorescent images of BiCD, Ber-NH_2_, and BiCD-Ber. (I) Fluorescent spectra of BiCD and BiCD-Ber.

In this study, BiCD-Ber was synthesized as illustrated in Figure 1A. First, potassium bismuth citrate and citric acid were adopted as the bismuth and carbon sources of bismuth-doped carbon dots (BiCD), respectively, with both reagents having good solubility in the water-formamide mixed solvent system used for the bottom-up hydrothermal method. Specifically, the citrates could enrich the BiCD with COOH groups and facilitate the incorporation of bismuth ions into the carbon dot skeleton, whereas formamide could contribute to the graphitic nitrogen embedded in the skeleton, granting the particles with fluorescence.^39^ Considering that berberine has no functional group for conjugation, Ber-NH_2_, a berberine derivative with a primary amine modified on its structure, was synthesized from berberine hydrochloride via a five-step reaction (Figure S1), with each step thoroughly characterized by nuclear magnetic resonance (NMR) spectroscopy and mass spectrometry before subsequent conjugation reactions (Figure S12–14). After BiCD was obtained as a black powder, Ber-NH_2_ was subsequently conjugated with BiCD through the 1-ethyl-3-(3-(dimethylamino)propyl) carbodiimide hydrochloride/*N*-hydroxysuccinimide (EDC/NHS) method to produce the final product, BiCD-Ber. This conjugation could introduce the positively charged quaternary ammonium isoquinoline structure to the CDs and change the net electrical charges to a certain extent. Consequently, it enhanced the interactions between the nanomedicines with negatively charged pathogens and host cells via electrostatic adsorption, and codelivered berberine and bismuth ions for a synergistic therapeutic effect.

The TEM images (Figure 1B and inset) with a measured average size distribution of 3.67 nm (Figure S15B) showed that the synthesized BiCD had a lattice spacing of approximately 0.31 nm, indicating a typical (100) in-plane lattice,^46^ whereas the initial investigation of the bismuth peak on the spectra of energy-dispersive X-ray (EDX) analysis suggested the successful doping of bismuth ions into the carbon dot skeleton (Figure S15A). Meanwhile, the X-ray diffraction (XRD) patterns of BiCD and BiCD-Ber exhibited a broad peak at a wide angle of 25° (Figure 1C), corresponding to the (002) plane of graphene.^47^ These results, along with the previously measured lattice spacing, confirmed the partial graphitic structure of the as-synthesized bismuth-doped CD. Additionally, the ζ-potential (Figure 1D) revealed that the net electrical charges of BiCD slightly increased from −8.10 to −4.36 mV after the conjugation. The negative charge of BiCD could be due to the carboxyl group on the particle surface, while BiCD-Ber was less negative as the conjugation converted some of the carboxyl groups into berberine bearing the quinolinium unit.

After the conjugation of Ber-NH_2_, multiple assays have been performed to verify the physiochemical changes of BiCD. Fourier transform infrared (FTIR) spectroscopy was first carried out to identify the functional groups in Ber-NH_2_, BiCD, and BiCD-Ber. As shown in Figure 1E, broad peaks observed between 3050–3300 cm^–1^ and 1550–1690 cm^–1^ in the spectra of BiCD were attributed to the νO–H and νC=O bands, suggesting that the presence of oxygen-containing functional groups on BiCD was readily available for EDC/NHS conjugation. Consequently, the appearance of a typical characteristic aromatic νC=C peak from Ber-NH_2_ at 1506 cm^–1^ in the BiCD-Ber spectrum confirmed the successful incorporation of Ber-NH_2_ into the carbon dots.^48^ Moreover, BiCD was extremely hygroscopic and showed good water solubility, owing to the abundant carboxyl groups on the particle surface, which simultaneously promoted extensive interaction with surrounding water molecules. In contrast, BiCD-Ber presented a mild hydrophobic property due to the conjugation of Ber-NH_2_, which occupied the hygroscopic carboxyl groups and introduced the hydrophobic isoquinoline and dimethoxybenzene moieties. To obtain a detailed elemental composition profile of the particles, XPS analysis was performed. As presented in Figure 1F, BiCD and BiCD-Ber had similar chemical states containing four fundamental elements, C, N, O, and Bi, with only slight differences after conjugation with NH_2_-modified berberine. Generally, the C 1s signal in BiCD could be differentiated into three bands: C–C/C=C (284.8 eV), C–O (287.1 eV), and C=O (288.6 eV), while the O 1s signal demonstrated two corresponding bands for C=O (531.6 eV) and C–O (533.1 eV). The C=O ratio in the O 1s spectrum of BiCD-Ber decreased alongside an increased C–O proportion compared to BiCD, possibly due to the formation of C–O bonds in conjugated Ber-NH_2_. Furthermore, since formamide was employed in the synthesis, N 1s was detected in BiCD with two distinct bands representing pyridinic-N and amine at 399.9 and 401.7 eV, respectively, whereas the successful doping of Bi in the carbon dots was confirmed by Bi 4f_7/2_ and Bi 4f_5/2_ at 159.5 and 164.8 eV, respectively.^49^ Collectively, the XPS analysis was consistent with the FTIR results, corroborating the successful embedding of Bi and Ber-NH_2_ conjugation on the surface of carbon dots.

The aqueous solutions of BiCD and BiCD-Ber at the same concentration also had different colors. Specifically, unmodified BiCD could be visually observed as reddish-brown, while BiCD-Ber appeared as a dark brown solution when viewed with the naked eye (Figure 1G). Referring to their fluorescence, BiCD showed a noticeable change after amine conjugation, with blue fluorescence under excitation at 365 nm significantly reduced and shifting toward a yellowish-green fluorescence (Figure 1H). Indeed, their fluorescence spectra provided further insights into the changes in the fluorescence properties (Figure 1I). Before conjugation, BiCD exhibited a general excitation-dependent emission with a maximum emission peak at 440 nm, which shifted to 530 nm with decreased intensity after conjugation. This shift and fluorescence quenching could be attributed to the inherent properties of Ber-NH_2_.^50^ Its absorption at 430 nm overlapped with the maximum emission of BiCD, thereby altering the overall emission of BiCD-Ber into a yellowish-green color with a static quenching effect (Figure S15C,D).^51^

### Antimicrobial and Antibiofilm Effects of BiCD, Ber-NH_2_, and BiCD-Ber

**Figure 2:**
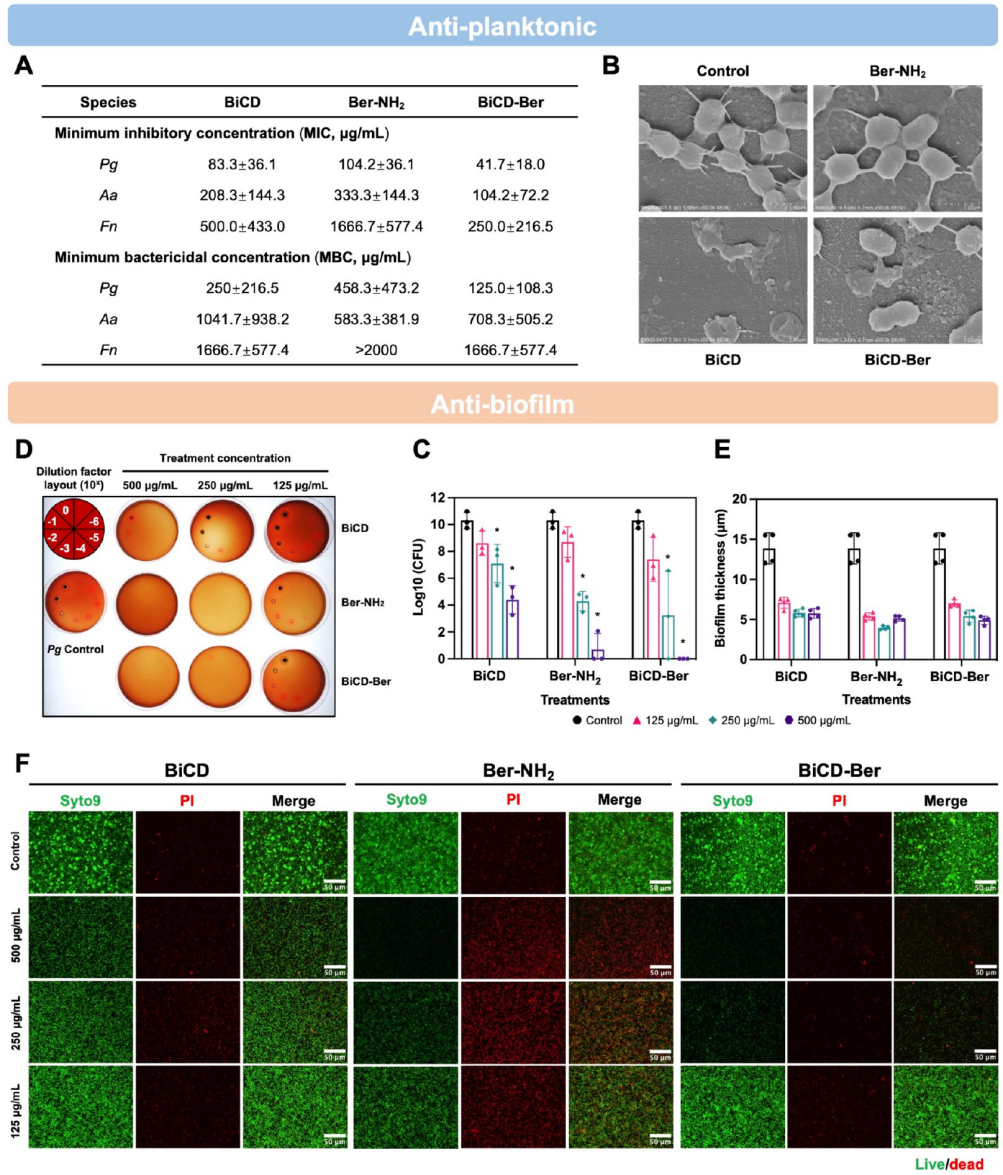
Antimicrobial and antibiofilm effects of BiCD, Ber-NH_2_, and BiCD-Ber. (A) Minimal inhibitory concentrations (MICs, μg/mL) and minimum bactericidal concentrations (MBCs, μg/mL) of BiCD, Ber-NH_2_, and BiCD-Ber against planktonic*P. gingivalis*(*Pg*),*A. actinomycetemcomitans*(*Aa*), and*F. nucleatum*(*Fn*). The assay was performed on three different occasions in duplicate, and the data are presented as the mean ± standard deviation (SD). (B) Morphology of *Pg* with or without treatment of BiCD, Ber-NH_2_, and BiCD-Ber at sub-MIC concentrations was assessed using field emission scanning electron microscopes (FE-SEM). (D) The 3-day-old *Pg* biofilms were treated with BiCD, Ber-NH_2_, and BiCD-Ber at different concentrations for 24 h, representative bacterial spots from *Pg* biofilms with/without treatments under different dilution factors showed the efficacy of treatments and reduction of *Pg* cells, and (C) the live bacteria in the biofilms were presented as log_10_ of the colony-forming units (CFUs). The assay was performed on three different occasions in duplicate, and the data are presented as mean ± SD. The asterisk (*) indicates the significant differences between the treatment and control groups (*p* < 0.05). (E) The thickness of *Pg* biofilms was determined from (F) the confocal images of the 3-day-old biofilms with/without treatments (scale bar: 50 μm).

The antimicrobial effects of BiCD, Ber-NH_2_, and BiCD-Ber were assessed against three selected periodontal pathogens, *Pg*,*Aggregatibacter actinomycetemcomitans*(*Aa*) and *Fusobacterium nucleatum* (*Fn*). Overall, both tested nanomedicines (BiCD and BiCD-Ber) and the synthetic small molecule (Ber-NH_2_) inhibited bacterial growth and demonstrated bactericidal effects at higher concentrations, except for Ber-NH_2_ on *Fn* (Figure 2A). It was noteworthy that *Pg* was more sensitive to the treatments, with lower minimum inhibitory concentration (MIC) and minimal bactericidal concentration (MBC) values, compared to the other two bacteria. In addition, it was found that BiCD and Ber-NH_2_ exhibited an additive effect on suppressing *Pg* with fractional inhibitory concentration (FIC) index values of 0.74 and 0.99 (Figure S16) according to the checkerboard assay. After the conjugation, BiCD-Ber displayed the lowest MIC and MBC values for *Pg* among the treatments, indicating enhanced antimicrobial capacity resulting from the combination of BiCD and Ber-NH_2_. Scanning electron microscopy (SEM) analysis was conducted to evaluate the *Pg* morphology after treatments at their sub-MIC concentrations (Figure 2B). Generally, while all treatment groups were able to effectively lower the total number of bacterial cells, the morphological changes in *Pg* could vary across treatments. Compared to the control group, Ber-NH_2_ treatment at sub-MIC concentration could reduce the bacteria count, but the remaining *Pg* retained their original shape. On the other hand, although both BiCD and BiCD-Ber were capable of reducing the total number of bacteria, the bacterial cells showed substantial morphological changes with large amounts of debris and fragments observed under SEM. These observations might be associated with the antimicrobial mechanisms of the tested nanomedicines and small molecules. In fact, Bi-doped carbon dots could inhibit bacterial growth by interfering with metabolic processes through the actions of bismuth ions. These metal ions, with a strong affinity to S-, N-, and O-containing functional groups, could disturb the activity of key enzymes or proteins with similar elemental functional residues (e.g., cysteine and histidine), consequently perturbing the iron-protein binding and ultimately leading to ferric deprivation-induced death.^52^

For the antibiofilm effect, the 3-day-old *Pg* biofilms were treated with BiCD, Ber-NH_2_, and BiCD-Ber at different concentrations (125, 250, and 500 μg/mL) for 24 h. After 24 h of treatment, the treated *Pg* samples were then diluted and spotted on blood agar plates to observe bacterial reduction by counting the number of visible colonies. As shown in Figure 2C,D, the *Pg* colonies on the blood agar plates confirmed the enhanced antibiofilm effect of BiCD-Ber compared to BiCD, and all treatments at 250 and 500 μg/mL could efficiently reduce *Pg* numbers in the biofilms. Of note, BiCD-Ber at 500 μg/mL completely eradicated the biofilms, whereas BiCD at the same concentration could only reduce bacteria counts by approximately 5 logs. Biofilms, as complex matrix-encased microbial communities, are capable of providing shelter for microbes due to the negatively charged extracellular polymeric matrix (EPS).^53^ Hence, the enhanced antibiofilm activity of BiCD-Ber may be attributed to the conjugation of Ber-NH_2_, which changes the ζ-potential of the nanomedicines. Moreover, the positively charged isoquinoline structure of Ber-NH_2_ promotes the interaction between BiCD-Ber and the negatively charged EPS, increasing the level of accumulation of BiCD-Ber within the biofilms. In terms of the effects of Ber-NH_2_ and nanomedicines on the thickness of *Pg* biofilms, confocal images with fluorescence staining were analyzed (Figure 2E,F). Our findings indicated that all treatments could significantly reduce the biofilm thickness (Figure 2E), and BiCD, Ber-NH_2_, and BiCD-Ber at 250 and 500 μg/mL greatly decreased the bacterial numbers compared to the control group, as evidenced by sparser green fluorescence signals with lower intensity overall (Figure 2F). It was also noted that *Pg* cells eradicated by BiCD and BiCD-Ber could not be stained with propidium iodide (PI), unlike those treated with Ber-NH_2_. This observation aligns with our previous work on Bi-related antimicrobials, which also showed that *Pg* cannot be stained with PI.^24^ This effect may be linked to the antimicrobial mechanism of bismuth ions, as doped Bi^3+^ could perturb *Pg* metabolism, increase oxidative stress, and consequently damage *Pg* DNA. As a result, there is a weaker binding with Syto9 and PI, leading to a diminished fluorescence signal under confocal.^54^

### Clearance of Extracellular and/or Intracellular *Pg* and the Accumulation of Nanomedicines in Host Cells

*Pg* can invade host cells (e.g., human gingival epithelial cells and human gingival fibroblasts) to evade immune clearance.^55−57^ As *Pg* could adhere and invade the host cells via different cell-surface ligands, it is therefore highly crucial to examine the anti-*Pg* effects of the as-synthesized nanomedicines on the *Pg*-infected host cells.

Prior to investigating the clearance effects on extracellular and intracellular *Pg*, the cytotoxicity of BiCD, Ber-NH_2_, and BiCD-Ber on primary gingival fibroblasts (pHGF) (Figure S17) and primary gingival epithelial cells (HGECs) (Figure S18) was evaluated using Cell Counting Kit-8 (CCK-8) and lactate dehydrogenase (LDH) assays, accompanied by optical images showing the cell status after each treatment. Generally, BiCD and BiCD-Ber demonstrated excellent biocompatibility at the tested concentrations, showing no detectable cytotoxicity. In contrast, Ber-NH_2_ reduced cell viability at 500 and 250 μg/mL. Furthermore, the charged Ber-NH_2_ conjugated with BiCD-Ber enhanced the hydrophobic properties of the nanoparticles, thereby facilitating their binding to cell membranes. This was evidenced by an increased presence of black deposits observed on cells that correlated with increasing BiCD-Ber concentrations under the microscope.

**Figure 3:**
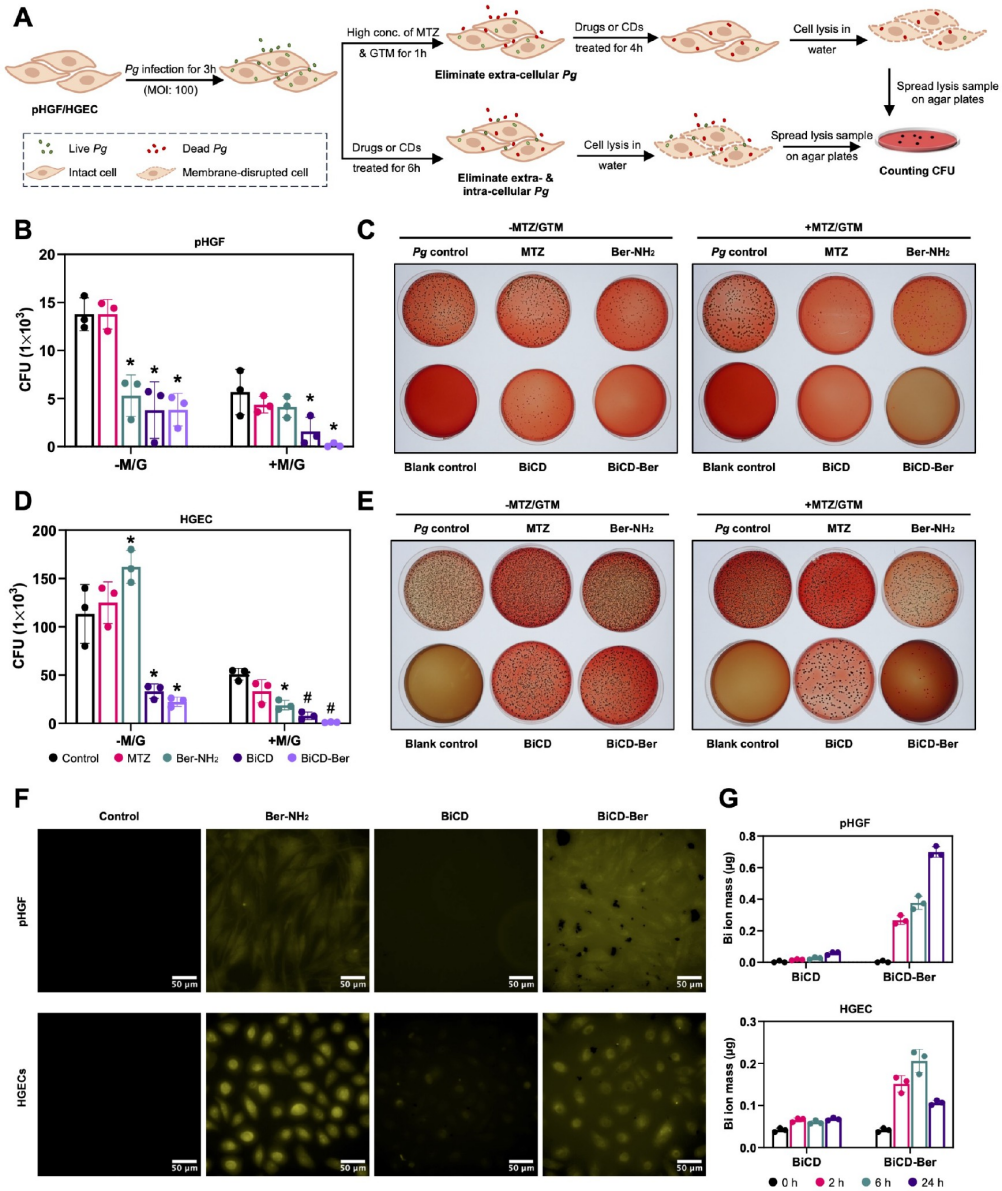
Clearance of extracellular and/or intracellular *Pg* and the accumulation of nanomedicines in host cells. (A) Illustration of the *Pg* infection, antibiotic washing, treatment duration, and evaluation in studying the clearance of extracellular and/or intracellular *Pg.* Part of the scheme was created in BioRender. (B) The number of live bacteria in *Pg*-infected (B) pHGF or (D) HGECs with (+M/G) or without (−M/G) antibiotics washing followed by treatments with BiCD, Ber-NH_2_, and BiCD-Ber for a certain period. The assay was conducted on three different occasions in triplicate, and the data are presented as mean ± SD. The asterisk (*) and pound (#) signs reflect the significant differences between the treatment and control groups with *p-*value <0.05 or 0.0001, respectively. Representative colony formation images of (C) pHGF or (E) HGECs lysis samples showing the eradication efficiency of the treatments. (F) Fluorescent images of BiCD, Ber-NH_2_, and BiCD-Ber-treated pHGF or HGECs for 4 h (scale bar: 50 μm). (G) Bismuth mass in pHGF or HGECs after being treated with BiCD or BiCD-Ber at 50 μg/mL for 2, 6, and 24 h. The experiment was performed on three different occasions in triplicate, and the figure is generated from one repeat; data are presented as mean ± SD.

Based on the cytotoxicity assays, a nontoxic concentration (100 μg/mL) was selected to investigate the anti-*Pg* effects of BiCD, Ber-NH_2_, and BiCD-Ber both extracellularly and/or intracellularly. As mentioned, *Pg* could evade host immunity and escape antibiotic clearance by invading and residing in the host cells. Therefore, it is crucial for developing effective anti-*Pg* drugs/treatments by taking into account the eradication of intracellular *Pg* cells. As illustrated in Figure 3A, two models were considered following *Pg* infection: one involving pretreatment with a high concentration of antibiotics (+M/G) to eliminate the extracellular bacteria and the other with the direct application of drugs or nanomedicine (−M/G). Briefly, two types of host cells were initially infected with *Pg* (followed by either the addition of antibiotics) and then exposed to drugs or nanomedicines at the predetermined cell-friendly concentration to examine their ability to eliminate extracellular and intercellular bacteria. Afterward, immunofluorescent staining (IFS), together with CFU analysis, was conducted to evaluate the antimicrobial effects of BiCD, Ber-NH_2_, and BiCD-Ber on *Pg*-infected cells.

IFS images of pHGF (Figure S19A) and HGEC (Figure S19B) showed that BiCD and BiCD-Ber-treated groups led to reductions in the green dots representing *Pg* cells. Furthermore, CFU results demonstrated that BiCD, Ber-NH_2_, and BiCD-Ber at 100 μg/mL were all effective in eradicating both extracellular and/or intracellular *Pg* compared to MTZ at 20 μg/mL in pHGF. In the +M/G model, after removing extracellular *Pg* using a high concentration of MTZ and gentamicin (GTM), all the treatment groups showed a decrease in intracellular *Pg* numbers, with the BiCD-Ber-treated group displaying the fewest bacteria, indicating its potent effect on eradicating intracellular bacteria (Figure 3B,C). Interestingly, although a similar pattern was observed in HGECs, significantly more *Pg* (approximately 10 times greater than in pHGF) were detected. (Figure 3D,E). This variance was attributed to the different expression of surface proteins and ligands in the two host cell types, resulting in varying affinities for *Pg*. Specifically, BiCD and BiCD-Ber were effective in eradicating most extra- and intracellular *Pg* compared to MTZ and Ber-NH_2_ in the −M/G model. Particularly, after elimination of the extracellular bacteria, it became more evident that BiCD and BiCD-Ber had potent anti-*Pg* effects against intracellular bacteria compared to the commonly used antibiotic, MTZ (+M/G model), with BiCD-Ber eradicating nearly 97.8% of intracellular *Pg*. Considering the significant clearance of both extracellular and/or intracellular *Pg* by the nanomedicines, especially BiCD-Ber, it was hypothesized that both the nanomedicines and bacteria were internalized by the cells through endocytosis.^58−61^ In fact, endocytosis, a process distinct from the uptake of small molecules like MTZ and Ber-NH_2_, may lead to the colocalization of nanomedicines and pathogens within the host cells, thereby facilitating targeted delivery and eradication. In addition, the varying efficiency in eradicating intracellular *Pg* by BiCD and BiCD-Ber could be attributed to the differences in their antibacterial potency as well as the variations in the amount of internalized nanomedicine influenced by particle charges.

To further explore the impact of particle charges on nanomedicine internalization, fluorescent images were captured after treating cells with BiCD, Ber-NH_2_, and BiCD-Ber at 50 μg/mL for 4 h (Figure 3F). BiCD exhibited minimal detectability in pHGF and relatively weak fluorescence in HGECs. Conversely, although the fluorescence of BiCD-Ber was lower than that of Ber-NH_2_, its signal could be clearly observed in both pHGF and HGECs, suggesting the accumulation of nanoparticles within the cells. Subsequent quantification of bismuth mass in the cells was conducted to investigate the internalization dynamics of BiCD and BiCD-Ber. As shown in Figure 3G, the bismuth mass remained relatively low even after 24 h of treatment with BiCD in pHGF or HGECs. However, when BiCD-Ber was applied, there was a noticeable increase in the bismuth content in comparison to BiCD-treated cells. This increase is likely attributed to the positively charged isoquinoline structure on BiCD-Ber, resulting in the nanomedicine having a less negative charge and facilitating its overall cellular uptake. Additionally, the intracellular bismuth mass increased in a time-dependent manner in pHGF with BiCD-Ber treatment, yet a decline was observed in HGECs at 24 h, indicating the potential excretion of BiCD-Ber by epithelial cells.

### BiCD and BiCD-Ber Restored *Pg*-Perturbed Immuno-Inflammatory Response and Protected Against *Pg*-Mediated Disruption of Cell Attachment

**Figure 4:**
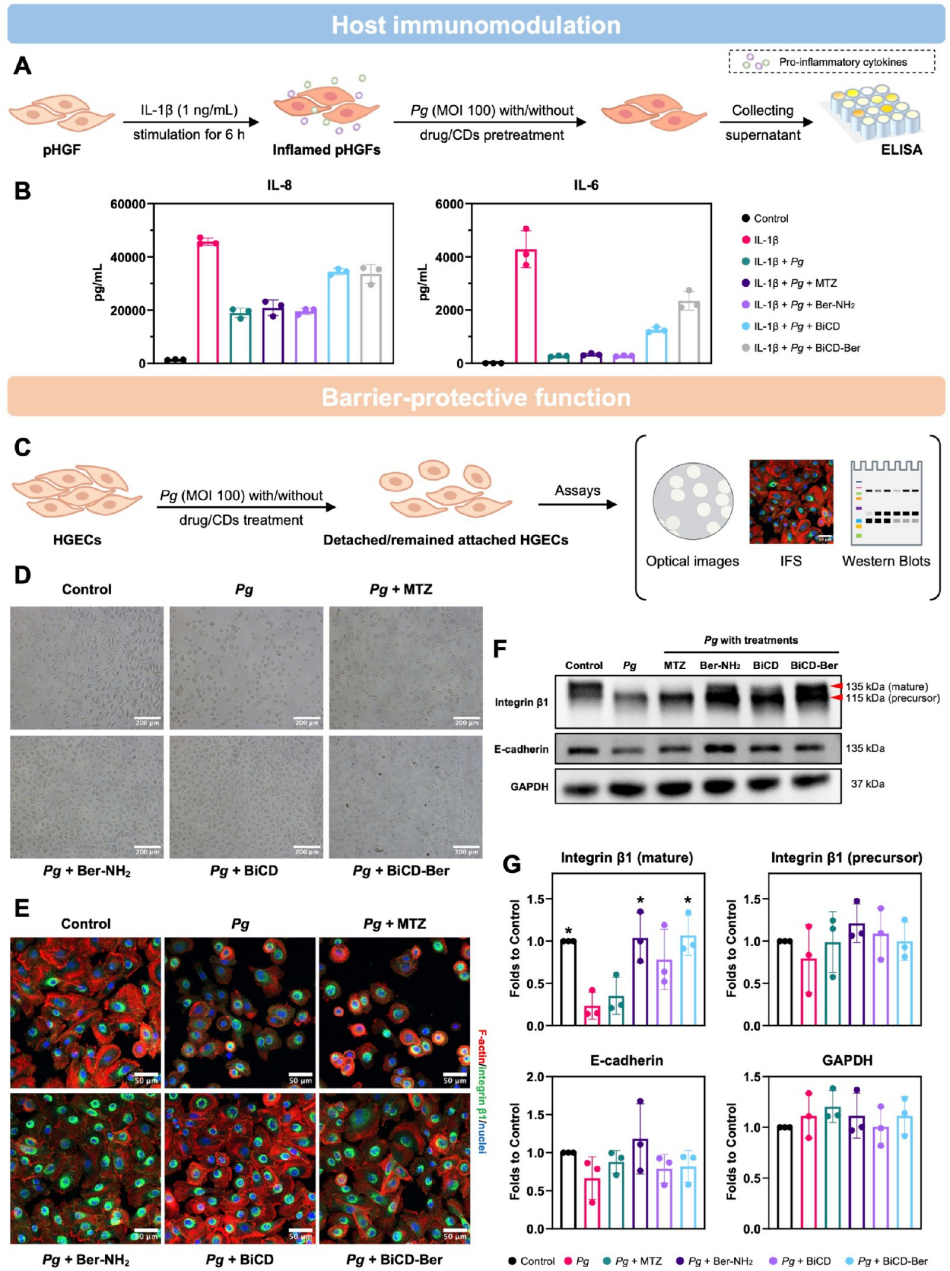
Bi-doped nanomedicines restored *Pg*-perturbed immuno-inflammatory response and protected against *Pg*-mediated disruption of cell attachment. (A) Schematic illustration of IL-1β-primed pHGF infected by *Pg* with/without treatments, followed by ELISA analysis of the pro-inflammatory cytokine levels in the supernatants; and the (B) concentrations of IL-8 and IL-6 in the supernatants from one representative biological repeat displayed as mean ± SD. The treatments and analysis were conducted for three different occasions in triplicate. (C) The effect of Ber-NH_2_, BiCD and BiCD-Ber on *Pg*-mediated HGEC detachment was investigated as illustrated. (D) The optical images of the cells infected by *Pg* with or without treatments indicate the cell attachment and junction status (scale bar: 200 μm). (E) Immunofluorescent staining of integrin β1 in HGECs followed by the previously mentioned treatments (scale bar: 50 μm). (F) Representative Western blots of integrin β1, E-cadherin, and GAPDH further revealed the effects of treatments on protecting against *Pg*-mediated digestion of cell-surface ligands. (G) The intensity of different protein blots was calculated from three biological repeats and statistically analyzed with the data presented as mean ± SD. The asterisk (*) indicates the significant differences between the control/treatment groups with the *Pg* group with *p-*value <0.05. Part of the scheme was created in BioRender.

*Pg* produces cysteine-based proteases (gingipains) that degrade key immune-regulatory cell surface proteins (e.g., CD31 and CD14) and cytokines (e.g., IL-6 and IL-8), thereby subverting host immunity.^10,62−66^ Additionally, gingipains disrupt epithelial integrity by cleaving junctional proteins like E-cadherin and integrin β1.^67,68^ These findings underscore the need for therapies that eliminate *Pg* while neutralizing its virulence factors to counteract these pathogenic effects. This study investigated the modulatory effects of synthesized nanomedicines on *Pg*-perturbed immune-inflammatory responses in pHGF. Specifically, the cells were first primed with IL-1β at 1 ng/mL for 6 h to induce the expression and secretion of pro-inflammatory cytokines, and then infected by *Pg* with or without treatments of drugs (MTZ or Ber-NH_2_) or nanomedicines (BiCD or BiCD-Ber). After 18 h, the supernatants were collected for ELISA analysis of IL-8 and IL-6 (Figure 4A). The results showed that the priming of IL-1β led to a significant increase in IL-8 and IL-6 expression levels, which were reduced after *Pg* infection, while *Pg* treated with MTZ or Ber-NH_2_ continued to decrease IL-8 and IL-6 levels. It is noteworthy that only treatments with BiCD and BiCD-Ber could partially reverse the decrease of cytokine previously induced by *Pg*, with BiCD-Ber exhibiting a stronger effect than BiCD on IL-6, implying the modulatory effects of synthesized nanomedicines on the *Pg*-perturbed innate immune response (Figure 4B).

As introduced, *Pg* can degrade cell-surface ligands or proteins, leading to cell detachment and disruption of the tissue structure. Therefore, this study also aimed to examine the protective effects of the as-synthesized nanomedicines on epithelial cells against *Pg*-mediated detachment. As shown in Figure 4C, confluent HGECs were infected with *Pg* with or without the treatment of nanomedicines for 24 h. The cell status and detachment were recorded and assessed using optical images, IFS, and Western blots. The results revealed that the *Pg*-infected group showed decreased cell density, with the remaining cells adopting a rounded morphology and losing their junctions. Likewise, cells infected with MTZ-treated *Pg* displayed characteristics similar to those treated with *Pg* alone. On the contrary, cells infected with *Pg* treated with Ber-NH_2_, BiCD, or BiCD-Ber did not detach from the plate, indicating protective effects against *Pg*-induced cell detachment (Figure 4D). Meanwhile, IFS analysis was conducted on HGECs under the same treatments to observe changes in integrin β1 and F-actin (Figure 4E). In the *Pg* and MTZ-treated *Pg* groups, the cell cytoskeleton was altered, with F-actin losing its filamentous shape. Conversely, cells infected with *Pg* treated with Ber-NH_2_, BiCD, and BiCD-Ber displayed intact cell junctions, well-distributed F-actin, and abundant integrin β1. As the primary antibody of integrin β1 used in this study could detect both mature (membrane-bound) and precursor (intracellular) forms of integrin β1, its paranuclear high-intensity signal was contributed by the precursor form, while the microspike-like low-intensity fluorescent signal at the cell edge indicated the membrane-bound mature form functioning in focal adhesion. A close-up look of the immunofluorescence images (Figures 4E and S20) illustrated that cells infected with Ber-NH_2_-, BiCD-, or BiCD-Ber-treated *Pg* presented well-stretched microspike-like integrin β1 at the cell edge, similar to the control group. In contrast, cells infected with *Pg* or MTZ-treated *Pg* showed a loss of the microspike-like integrin β1. Importantly, the mature form of integrin β1 was degraded by *Pg* and MTZ-treated *Pg*, but this degradation could be prevented in the presence of Ber-NH_2_, BiCD, and BiCD-Ber (Figure 4F,G). Moreover, the groups treated with BiCD-Ber and Ber-NH_2_, along with the control group, displayed significantly more mature integrin β1 compared to the *Pg*-treated group. Yet, no differences were observed in the precursor form among all groups, suggesting that this protective effect primarily occurred extracellularly. While optical and immunofluorescent imaging revealed the loss of cell junctions in *Pg*-infected groups, E-cadherin levels remained statistically unchanged compared to the treatment and control groups (Figure 4G). This might be due to the insufficient expression of E-cadherin in HGECs under two-dimensional culture, and the *Pg* infections and other treatments may primarily affect integrin β1 rather than E-cadherin.

Collectively, our findings demonstrate that bismuth-doped nanomedicines (BiCD and BiCD-Ber) counteract *Pg*-perturbed immune responses and protect against tissue destruction through a synergistic interplay between bismuth ions and berberine. Given the key role of *Pg*-secreted gingipains in subverting host immune responses and degrading cell surface ligands, we propose that BiCD-Ber exerts immunomodulatory and barrier-protective effects by directly inhibiting gingipain activity.

### BiCD and BiCD-Ber Suppressed the Proteinase Activity of Recombinant Truncated RgpB and Kgp

**Figure 5:**
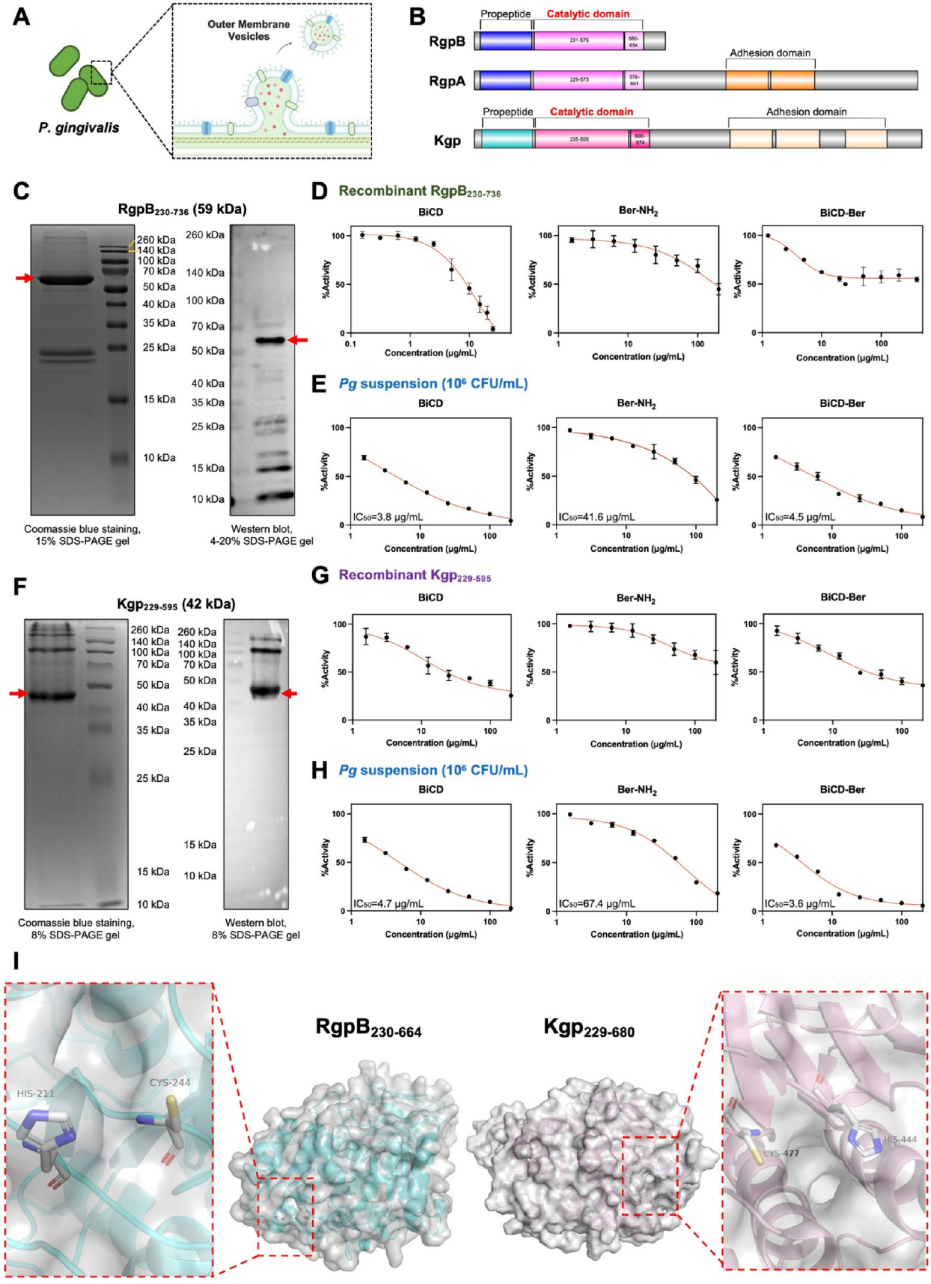
(A) *Pg* could generate outer membrane vesicles containing different membrane proteins and various virulence factors including gingipains. Part of the scheme was created in BioRender. (B) Schematic diagram of the structural domains of different gingipains. (C) Well-expressed and purified RgpB_230–736_ was examined by using Coomassie brilliant blue (CBB) staining and Western blot analysis. Red arrows pointing at the protein bands with their correlated molecular weight. The inhibitory effects of BiCD, Ber-NH_2_, and BiCD-Ber were investigated on (D) the amidolytic activity of recombinant RgpB_230–736_ and (E) the arginine-specific amidolytic activity of *Pg* suspension. (F) Recombinant Kgp_229–595_ was also expressed and examined using CBB and Western blot analysis. The suppression by BiCD, Ber-NH_2_, and BiCD-Ber on (G) the recombinant Kgp_229–595_ and (H) the lysine-specific amidolytic activity of the *Pg* suspension was studied *in vitro*. (I) Crystal structures of RgpB_230–664_ (PDB:1CVR) and Kgp_229–680_ (PDB:4TKX) with their catalytic pockets (red dashed square) showing the active sites composed of a histidine and a cysteine residue.

*Pg* is able to generate outer membrane vesicles containing enriched membrane proteins and various virulence factors, such as lipopolysaccharides, fimbriae, and gingipains, which play a role in the progression of periodontitis (Figure 5A).^69^ Among these, gingipains are cysteine-based proteinases categorized under the peptidase family C25,^70^ which can disrupt host innate immune responses via degrading cytokines, cell-surface ligands, and complements.^71^ Currently, two types of gingipains have been identified: arginine-specific gingipains (RgpA and RgpB) and lysine-specific gingipain (Kgp). Precisely, RgpA and RgpB share a high level of sequence similarity (approximately 97%) in their catalytic domains, while both RgpA and Kgp are composed of a signal peptide, an N-terminal propeptide domain, an arginine-specific or lysine-specific catalytic domain, and a large C-terminal adhesion domain (Figure 5B). Since the catalytic domains of the gingipains function as the key components in degrading biomacromolecules, plasmid construction was performed based on the sequences in the catalytic domains of RgpB and Kgp using pET28a. Truncated proteins representing the active regions of both arginine- and lysine-specific gingipains were expressed in*E. coli*BL21 (DE3) and then purified as described in the supplementary file.

The successful expression and purification of truncated RgpB (RgpB_230–736_, residues 230–736, 59 kDa) were verified by Coomassie blue staining and Western blot analysis (Figure 5C). BiCD, Ber-NH_2_, and BiCD-Ber exhibited distinct inhibitory effects on the proteinase activity of RgpB_230–736_, with BiCD showing the most potent inhibitory effect (Figure 5D). Additionally, the tested nanomedicines or compounds also suppressed the arginine-specific amidolytic activity of *Pg* cells, with the prominent inhibitory effect observed for BiCD and BiCD-Ber, as determined by their respective IC_50_ values (3.8 and 4.5 μg/mL, respectively) (Figure 5E). Comparably, the truncated Kgp (Kgp_229–595_, residues 229–595, 42 kDa) was also expressed (Figure 5F), and BiCD, Ber-NH_2_, and BiCD-Ber inhibited the proteinase activity of Kgp_229–595_ (Figure 5G), as well as the lysine-specific amidolytic activity of *Pg* cells (Figure 5H). BiCD displayed superior inhibitory effects on the recombinant RgpB_230–736_ compared to BiCD-Ber, possibly due to its greater water solubility, facilitating closer binding/interaction with the protease. Notably, both BiCD and BiCD-Ber demonstrated stronger inhibitory effects than Ber-NH_2_ on *Pg* cells, suggesting that these two nanomedicines were effective in suppressing the bacterial membrane-bond gingipains. Here, the protein structures, together with catalytic pockets of RgpB_230–664_ (PDB: 1CVR) and Kgp_229–680_ (PDB: 4TKX), are illustrated in Figure 5I, showing that both proteins have a pair of active sites with histidine acting as the proton donor and the cysteine residue as a nucleophile. Meanwhile, bismuth ions have been reported to have a high affinity for N-, O-, and S-based ligands.^72^ Thus, the suppression of recombinant gingipains and *Pg* cells arises from Bi^3+^ in Bi-doped nanomedicines binding to functional groups (imidazole and thiol) at the gingipain active sites, sterically hindering substrate access. This mechanism elucidates how BiCD-Ber not only eradicated *Pg* but also neutralized its virulence, as demonstrated by preserved integrin β1 levels and restored pro-inflammatory cytokine balance (Figure 4). These results directly link gingipain inhibition to the observed therapeutic outcomes, including immune modulation and epithelial barrier protection. By targeting both bacterial viability and virulence factors, BiCD-Ber offered a comprehensive strategy to disrupt *Pg* pathogenesis.

### Construction of *Pg*-Responsive Hydrogel with Encapsulation of BiCD-Ber

**Figure 6:**
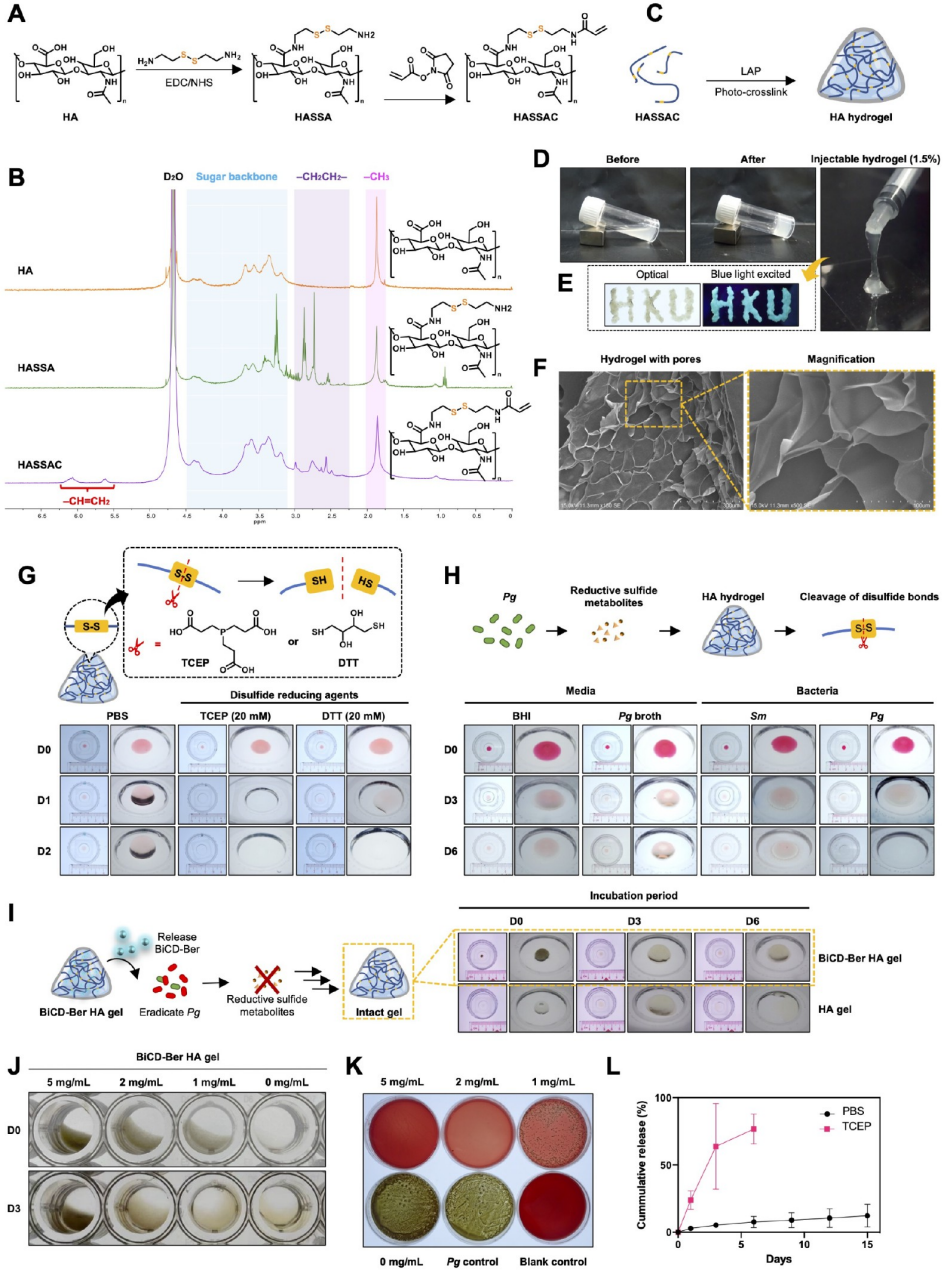
Construction of injectable *Pg*-responsive hydrogel for delivering BiCD-Ber. (A) A two-step structure modification of hyaluronic acid (HA) was conducted to obtain a disulfide-modified and acrylated HA derivative (HASSAC). (B) The successful modifications were confirmed by using ^1^H-NMR. (C) The proposed HA hydrogel was synthesized by photo-cross-linking HASSAC, and part of the scheme was created in BioRender. (D) The aqueous solution of HASSAC lost its fluidity after blue-light irradiation, while the hydrogel prepared from HASSAC at 1.5% (*w*/*v*) was injectable. (E) The injectable hydrogel with encapsulation of BiCD-Ber exhibited excellent shape adaptability and writing capability, as well as fluorescence under excitation. (F) SEM images of fabricated hydrogel displayed its porous structure, enabling the encapsulation of as-synthesized nanomedicines. (G) The constructed hydrogel loaded with red dye was proposed to degrade by disulfide-reducing reagents, such as TCEP and DTT, which were verified in subsequent experiments. (H) *Pg* was able to produce reductive sulfide metabolites and degrade hydrogel (upper scheme), and *Pg*-induced hydrogel degradation was compared to the*S. mutans*(*Sm*)-cocultured hydrogel for 6 days to verify the pathogen-specific degradation mode. (I) The BiCD-Ber encapsulated HA gel was designed to release BiCD-Ber to eliminate *Pg* while keeping the hydrogel intact (upper scheme), as confirmed in a 6-day incubation with *Pg* suspension. (J) BiCD-Ber HA gel with varying BiCD-Ber concentrations was tested against *Pg* at 10^8^ CFU/mL for 3 days. (K) After treatment, the supernatants were collected and applied onto blood agar plates followed by a 7-day anaerobic culture for determining the anti-*Pg* effects of BiCD-Ber HA gel. (L) Releasing profiles of BiCD-Ber from BiCD-Ber HA gel (2 mg/mL) with or without TCEP as a disulfide-reducing reagent.

To construct the pathogen-responsive hydrogel, a disulfide-modified and acrylated derivative of hyaluronic acid (HASSAC) was synthesized from hyaluronic acid (HA) through a two-step reaction (Figure 6A). The proton nuclear magnetic resonance (H-NMR) spectra of HA and compounds obtained in each step are illustrated in Figure 6B. The peak at 1.9 ppm corresponds to the methyl protons on the HA backbone, while those in the range of 3.1–4.5 ppm represent the protons in the sugar backbone (d-glucuronic acid and d-*N*-acetyl glucosamine) of HA. Following the first-step reaction, new peaks emerged at 2.3–3.0 ppm, indicating the protons in −CH_2_CH_2_– from the conjugated cystamine. After the second-step reaction, peaks at 5.6 and 6.1 ppm verified the successful acrylation of HA. The final compound (HASSAC), containing disulfide bonds and acrylate groups, was dissolved in phosphate-buffered saline (PBS) with lithium phenyl-2,4,6-trimethylbenzoylphosphinate (LAP) serving as a photoinitiator to form the HA hydrogel under ultraviolet (UV) irradiation at 405 nm for 30 s (Figure 6C). The concentration of the synthesized HASSAC could directly affect the mechanical properties of the hydrogels. Therefore, in this study, hydrogels were fabricated with varying HASSAC concentrations (1.5% and 2.0%), and their mechanical properties were evaluated through rheological analysis. Both hydrogels exhibited storage moduli (G’) exceeding loss moduli (G’’), confirming their elastic behavior (Figure S21A). Notably, the 1.5% HASSAC hydrogel demonstrated significantly lower viscosity compared with the 2.0% formulation. Moreover, its viscosity decreased more rapidly with increasing shear rates, indicative of pronounced shear-thinning behavior (Figure S21B). This enhanced shear responsiveness, coupled with reduced structural stability, renders the 1.5% HASSAC hydrogel more suitable for injectable delivery into irregular periodontal pockets, where adaptability to anatomical contours is critical. Moreover, the 1.5% HASSAC hydrogel was able to pass through a syringe hub without clogging, demonstrating its excellent injectability (Figure 6D). Furthermore, the hydrogel with encapsulation of BiCD-Ber exhibited excellent shape adaptability and writing capability (as shown in the photo of the written “HKU” letters), as well as fluorescence under excitation, making it suitable for injection into periodontal pockets (Figure 6E). SEM analysis revealed that the hydrogel contained a porous structure capable of accommodating the synthesized nanomedicines for subsequent tests (Figure 6F).

Due to the presence of disulfide bonds in HASSAC, the fabricated hydrogel could undergo degradation mediated by the disulfide-reducing reagents. To confirm this, two representative disulfide-reducing reagents, tris(2-carboxyethyl)phosphine hydrochloride (TCEP) and dithiothreitol (DTT), were applied to the well-constructed rhodamine 123-loaded hydrogels to observe the disulfide bond cleavage-based degradation. As anticipated, both TCEP- and DTT-treated hydrogels lost their integrity after 1 day of treatment. Specifically, the hydrogel treated with TCEP completely dissolved by Day 1, while the one treated with DTT disintegrated on Day 2 (Figure 6G). It has been well documented that *Pg* can produce volatile reductive sulfide metabolites, such as hydrogen sulfide and methanethiol,^73,74^ implying its potential to degrade the disulfide-bond-rich hydrogel. In comparison, another oral pathogen associated with dental caries,*Streptococcus mutans*(*Sm*), capable of producing acid and decreasing the environmental pH value, was chosen to study the specificity of pathogen-responsive hydrogel degradation. As indicated in Figure 6H, *Pg-*treated hydrogel collapsed into a flat shape on Day 3 and completely decomposed by Day 6, while *Sm*-treated hydrogel only underwent slight swelling but maintained an intact structure, suggesting a *Pg*-specific degradation of the hydrogel. Notably, this hydrogel system with encapsulation of BiCD-Ber (BiCD-Ber HA gel) was able to release BiCD-Ber in an environmentally responsive manner, eliminating *Pg*, reducing and neutralizing the reductive sulfide metabolites, and consequently resisting *Pg*-mediated degradation (Figure 6I).

Regarding the anti-*Pg* effect of BiCD-Ber HA gels, hydrogels with different concentrations of encapsulated BiCD-Ber were constructed in a 96-well plate and cultured with a *Pg* suspension at 10^8^ CFU/mL for 3 days. Except for the 5 and 2 mg/mL groups, both the 1 mg/mL and blank hydrogels showed substantial morphological changes, indicating the breakdown of the hydrogel network and inadequate resistance to *Pg* treatment (Figure 6J). The growth of *Pg* on blood agar plates after coculturing with BiCD-Ber HA gels confirmed the anti-*Pg* effects, with the 5 and 2 mg/mL hydrogels nearly eradicating all *Pg* cells, while the 1 mg/mL group partially reduced the *Pg* count (Figure 6K). The obtained results were consistent with the levels of gel degradation observed. Furthermore, the release of BiCD-Ber from the BiCD-Ber HA gel was studied in PBS with or without a disulfide-reducing reagent (Figure 6L). There was minimal release of BiCD-Ber (around 12.3%) from BiCD-Ber HA gel in PBS over 15 days, while the release percentage reached approximately 76.7% on Day 6 in TCEP solution, indicating a disulfide-bond-cleavage-based release mode.

### Therapeutic Effects of BiCD-Ber HA Gel on Experimental Periodontitis in Rats

**Figure 7:**
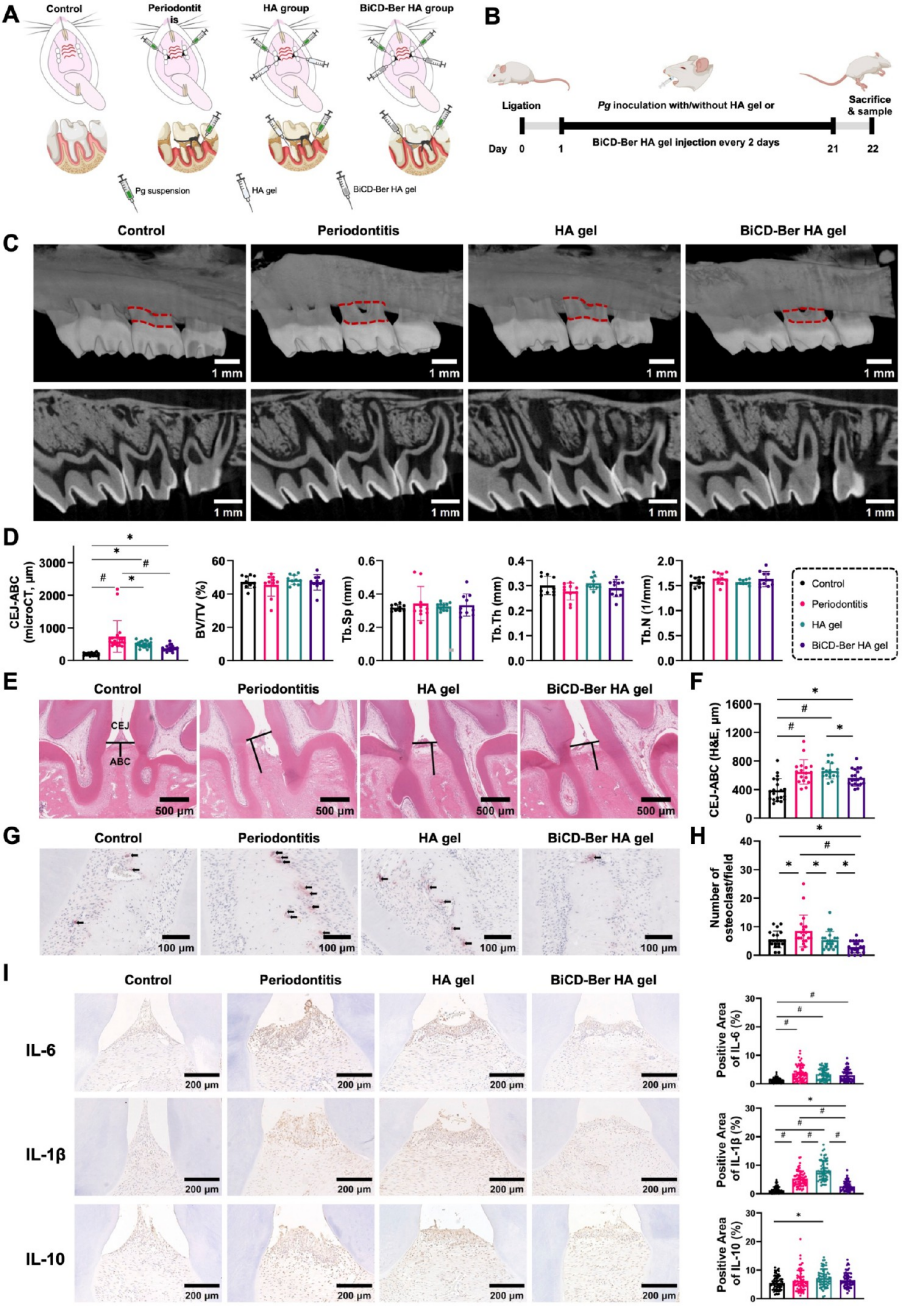
Therapeutic effects of BiCD-Ber HA gel on experimental periodontitis *in vivo*. (A) Schematic illustrations of the grouping and correspondent treatments in each group. (B) Experimental schedule of the *in vivo* study. Part of the scheme was created in BioRender. (C) Representative reconstructed microcomputed tomography (micro-CT) and sagittal images of the maxillary molars from each group (scale bar: 1 mm). Cementum-enamel junction (CEJ) and alveolar bone crest (ABC) were highlighted with red dashed lines. (D) The distance between the CEJ and ABC (CEJ-ABC), bone volume per total volume (BV/TV), trabecular separation (Tb. Sp), trabecular thickness (Tb. Th), and trabecular number (Tb. N) of maxillary alveolar bone surrounding the second molar of each group. (E) Representative histological staining images displaying periodontal tissue of maxillary second molars from different groups, with lines indicating the distance between CEJ and ABC (scale bar: 500 μm). (F) Statistical analysis of the distance between CEJ and ABC obtained from histological staining (*n* = 10 per group). (G) Representative TRAP staining images of each group. Black arrows indicate the positively stained multinucleated osteoclasts (scale bars: 100 μm). (H) Statistical analysis of the active osteoclast numbers surrounding the second molar of each group (*n* = 10 per group). (I) Representative immunohistochemical staining of IL-6, IL-1β, and IL-10 at gingiva tissues from each group (scale bar: 200 μm) and corresponding statistical analysis of each cytokine (*n* = 60 per group). The immunohistochemical images were analyzed using ImageJ. For each sample, six regions of interest (ROIs) were randomly selected, and color deconvolution was applied to isolate 3,3′-diaminobenzidine (DAB) staining. The positive area percentage was calculated using thresholding, ensuring consistent parameters across all images. All the data in the bar charts are presented as mean ± SD, and the statistical analyses are presented as asterisk (*) and pound (#) signs with *p-*value <0.05 or 0.0001, respectively.

An experimental periodontitis model was established in rats through ligation and *Pg* inoculation to evaluate the effects of nanomedicines delivered by the constructed hydrogel *in vivo*. In the treatment groups, either blank HA gel or BiCD-Ber HA gel was injected into the ligatured area along with *Pg* inoculation (Figure 7A). The entire treatment lasted for 21 days, and all the rats were sacrificed to collect their tissue samples on Day 22 (Figure 7B). All procedures were ethically approved by the Ethics Committee for Animal Experiments of Sun Yat-sen University (No. SYSU-IACUC-2023–001853).

To evaluate the *in vivo* biocompatibility of HA gel and BiCD-Ber HA gel, hematoxylin and eosin (H&E) staining of major organs collected from different groups was conducted. No obvious inflammation or destruction was found in any organs (Figure S22), suggesting the favorable biosafety of the treatments. As depicted in the reconstructed micro-CT and sagittal images (Figure 7C), the periodontitis group displayed significant bone loss compared to the control group, suggesting the successful establishment of the experimental periodontitis model. In contrast, both HA gel and BiCD-Ber HA gel displayed therapeutic effects and prevented the bone loss to some extent. Quantitative analysis of the distance between the cemento-enamel junction and alveolar bone crest (CEJ-ABC) of maxillary alveolar bone in each group further verified the positive effects of BiCD-Ber HA gel (Figure 7D), as the distance in BiCD-Ber HA gel was significantly lower (*p* < 0.0001) than that in the periodontitis group. In addition, no significant differences in bone morphology parameters (e.g., bone volume per total volume (BV/TV), trabecular separation (Tb. Sp), trabecular thickness (Tb. Th), and trabecular number (Tb. N)) were found among the groups.

Apart from Micro-CT, the distance between the CEJ and ABC was also examined in H&E-stained slides (Figure 7E). The representative slides indicated obvious bone resorption in the periodontitis group, along with the long CEJ-ABC distance. Typically, the treatments of both HA gel and BiCD-Ber HA gel exhibited a trend toward shortening the CEJ-ABC distance compared to the periodontitis group, while the CEJ-ABC distance in the BiCD-Ber HA gel group was significantly lower than that in the HA gel group (*p* < 0.05), suggesting less alveolar bone loss in the BiCD-Ber HA gel group (Figure 7F). Alveolar bone loss is considered the hallmark of periodontitis advancement, and it remains a significant challenge to prevent alveolar bone loss in periodontal treatment. Current evidence has indicated that bone destruction is mainly mediated by host immuno-inflammatory responses to microbial invasion.^75^ The multinucleated osteoclasts, differentiated from the macrophage cell lineage, are activated in periodontitis and are capable of destroying and resorbing bone tissue.^76^ In this study, the osteoclasts in the alveolar bone from different groups were stained by their biomarker, tartrate-resistant acid phosphatase (TRAP) (Figure 7G). It was noted that the highest number of positively stained osteoclasts was found in the periodontitis group compared with the other three groups, correlating with the most significant bone loss level observed in the analysis of CEJ-ABC distance. In fact, the osteoclast number in the BiCD-Ber HA gel group was significantly lower than in the periodontitis (*p* < 0.0001) and HA gel (*p* < 0.05) groups (Figure 7H), suggesting effective control of the inflammatory response. Immunohistochemistry (IHC) staining further indicated the anti-inflammatory effects of the BiCD-Ber HA gel against ligation- and *Pg* inoculation-induced periodontitis (Figure 7I). For the pro-inflammatory cytokines, such as IL-6 and IL-1β, the positively stained cells in the BiCD-Ber HA gel group were less than those in the periodontitis group, presenting a lighter brown color in the gingiva tissue. However, referring to the anti-inflammatory cytokine IL-10, it was found that the expression of IL-10 in gingiva tissue in the treatment groups did not show a significant difference compared to the periodontitis group. Collectively, these results confirmed that the BiCD-Ber treatment delivered via HA gel could control the inflammatory responses in gum tissue by inhibiting the expression of pro-inflammatory cytokines.

### Hydrogel and Nanomedicines-Mediated Shifting of Subgingival Microbiota

**Figure 8:**
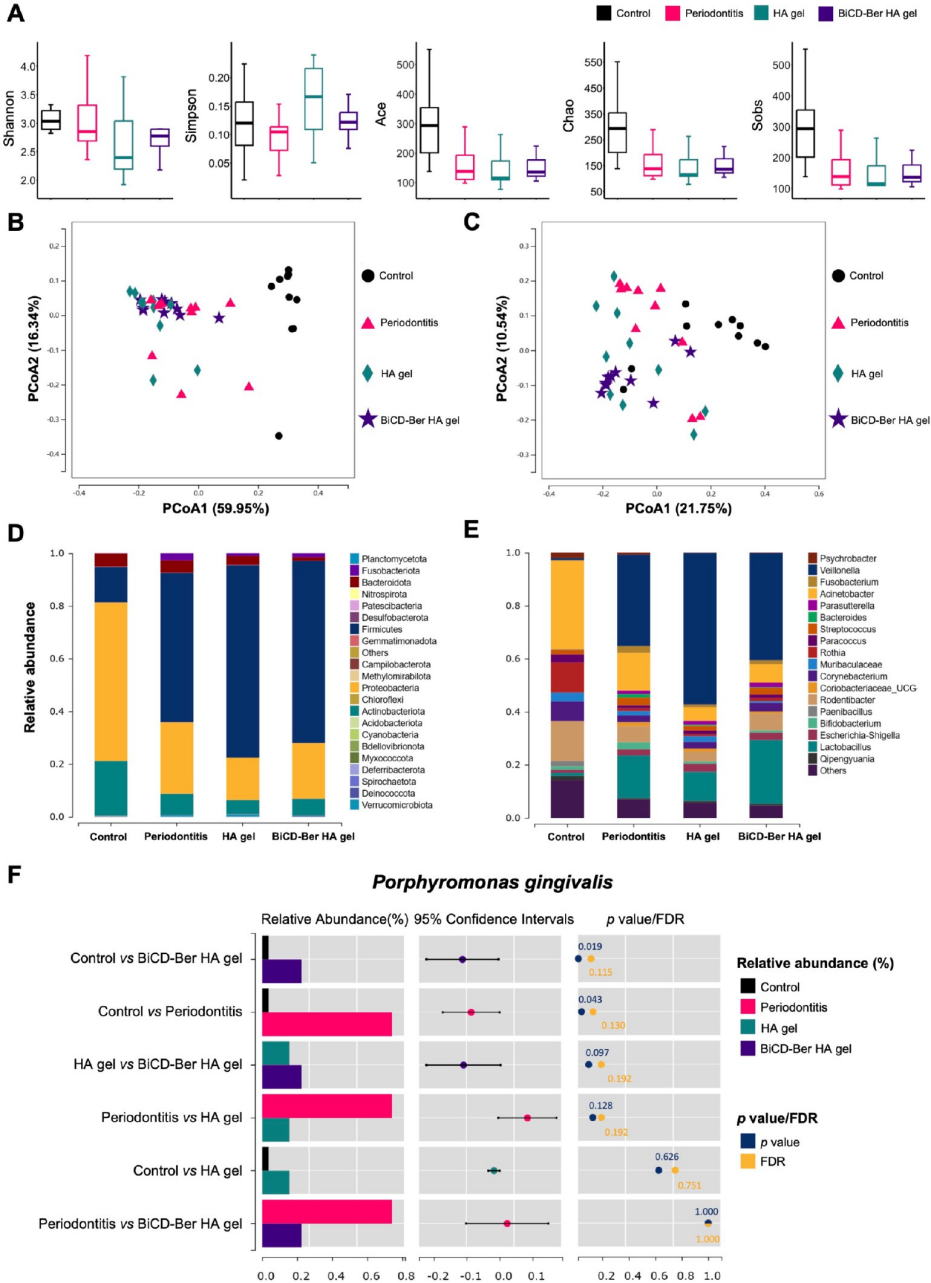
(A) Alpha diversity metrics of Shannon, Simpson, Ace, Chao, and Sobs among different groups. Principal coordinates analysis (PCoA) of (B) the weighted UniFrac and (C) unweighted UniFrac distance categorized by different groups. The relative abundance of periodontal microbiota at the (D) phylum and (E) genus levels among different groups. The phyla/genera with a relative abundance of less than 0.5% in the samples are merged into the ″Others″ item. (F) The relative abundance of *Pg* among different groups and the relevant analyses. The left bar chart shows the comparison between two randomly assigned groups, and the middle column displays the log2 value of the average relative abundance ratio between every two groups, while the right column presents the *p* and FDR values of the comparison.

Increasing evidence suggests that the dysbiotic polymicrobial communities play a crucial role in the development of periodontitis.^77^ Alterations in the subgingival microbiota can disrupt the delicate balance of host-microbial interactions, potentially leading to the onset of periodontal disease. Hence, the profiles of subgingival microbiota in different groups were assessed to investigate the potential impacts of the as-synthesized hydrogel and nanomedicines on dysbiotic microbiota. The alpha diversity of each group was first examined (Figure 8A). Although no significant differences were observed in alpha diversity metrics, including Shannon and Simpson indices for community diversity and Ace, Chao, and Sobs indices for community richness, the control group displayed a higher trend in community richness compared to other groups, while the BiCD-Ber HA group exhibited less variation. This indicates similar levels of biodiversity in terms of alpha diversity across the groups.

Furthermore, beta diversity analysis was conducted to compare the respective community structures. The overall bacterial community compositions in each site were compared based on their weighted/unweighted unique fraction metric (UniFrac) distances and visualized through principal coordinates analysis (PCoA) plots. Two PCoA plots were generated with two principal axes (PCoA1 and PCoA2), which explained 59.95% and 16.34% of the variance, respectively; based on the weighted UniFrac distances (Figure 8B), and 21.75% and 10.54% of the variance based on the unweighted UniFrac distances (Figure 8C) among the bacterial communities within each site. Significant compositional differences across groups were detected in the PCoA analysis of both weighted and unweighted UniFrac distances. Particularly, the ligation and *Pg* inoculation significantly altered the composition of the subgingival microbiota compared with the control group. Moreover, the BiCD-Ber HA group also showed a significant compositional difference with the periodontitis group based on the unweighted UniFrac distances (*p* < 0.001), implying a potential influence of the treatment on the dysbiotic microbiota resulting from the treatment.

To delve deeper into taxonomic composition and explore potential biomarkers, the relative abundance at the phylum and genus levels in each group was examined and is presented in Figure 8D,E, respectively. A diverse range of bacterial taxa, spanning 22 phyla, was identified in the samples. As shown in Figure 8D, *Proteobacteria*, *Firmicutes*, and *Actinobacteriota* emerged as the dominant phyla. Significant differences in the relative abundances of five phyla—including *Chloroflexi*, *Proteobacteria*, *Fusobacteriota*, *Actinobacteriota*, and *Firmicutes*—were observed among the groups. Additionally, 40 genera and 73 species exhibited significant variations across the groups (Table S2). Moreover, the relative abundance of the keystone pathogen *Pg* was selected and analyzed in each group, revealing a decreasing trend in the HA gel and BiCD-Ber HA gel groups compared to the periodontitis group (Figure 8F). Since the treatment only lasted for 21 days, the changes in the microbiota caused by the treatments would likely be more pronounced over a longer treatment period. Finally, linear discriminant analysis effect size (LEfSe) analysis identified biomarkers from 5 phyla, including *Firmicutes*, *Proteobacteria*, *Gemmatimonadota*, *Fusobacteriota*, and *Actinobacteriota* (Figure S23), highlighting potential indicators of group differentiation.

## Conclusions

In conclusion, this work successfully synthesized bismuth-doped carbon dots and conjugated them with a modified berberine derivative (Ber-NH_2_) to develop the BiCD-Ber nanomedicine. This nanomedicine demonstrated potent antimicrobial effects against the keystone pathogen, *Pg*, under various conditions, particularly in eradicating *Pg* that resided in host cells. The modification of Ber-NH_2_ enhanced the antimicrobial efficacy of BiCD due to its higher affinity to pathogens and host cells, resulting in a higher level of intracellular accumulation. Moreover, BiCD-Ber was effective in reversing *Pg*-perturbed immune responses in gingival fibroblasts and protecting against the *Pg*-mediated disruption of cell attachment in gingival epithelial cells. The underlying mechanism is likely related to the inhibition of *Pg*-produced gingipains by BiCD-Ber. To facilitate the delivery of BiCD-Ber *in vivo*, an injectable hyaluronic acid–based hydrogel capable of responding to *Pg* metabolites was constructed to encapsulate and release BiCD-Ber in a controlled manner. Our data demonstrated that the BiCD-Ber HA gel could effectively reduce bone tissue destruction, osteoclast infiltration, and inflammation in the gingiva of rats in the experimental periodontitis model. Importantly, the BiCD-Ber HA gel demonstrated modulatory effects on subgingival microbiota. Overall, the BiCD-based nanomedicine encapsulated in a pathogen-responsive hydrogel system holds significant promise for clinical translation in periodontitis management due to its multifunctional and antibiotic-free design. Unlike conventional antibiotics, the constructed BiCD-Ber could address the multifactorial nature of periodontal diseases by simultaneously eradicating the keystone pathogen, neutralizing virulence factors, modulating host inflammation, and protecting epithelial barriers. The pathogen-responsive degradation of the hydrogel ensures localized drug release within dysbiotic periodontal pockets, minimizing off-target effects and enhancing therapeutic precision. Meanwhile, the gradual degradation of the hydrogel system aligns with the chronicity of periodontitis, enabling long-term microbial control and inflammatory modulation. While the BiCD-Ber HA gel system is promising, several challenges must be addressed to advance this technology to the clinic, such as the scalability and manufacturing of the CD and hydrogels, comprehensive preclinical safety profiling of the BiCD-Ber HA gel system, and patient acceptance of the locally injected nanomedicine-encapsulated hydrogel. Although challenges exist, the BiCD-Ber hydrogel system offers a compelling solution to unmet needs in periodontal therapy. With strategic partnerships and continued innovation, this platform has strong potential to transition from bench to bedside.

## Experimental Section

### Chemicals

Citric acid, *N*-hydroxysuccinimide (NHS), metronidazole (MTZ), gentamicin (GTM), cystamine dihydrochloride, rhodamine 123, dithiothreitol (DTT), and dimethylformamide (DMF) were purchased from Sigma-Aldrich (St. Louis, USA). Potassium bismuth citrate, 1-ethyl-3-(3-(dimethylamino)propyl) 1-ethyl-3-(3-(dimethylamino)propyl) carbodiimide hydrochloride (EDC), hyaluronic acid, *N*-succinimidyl acrylate, lithium phenyl-2,4,6-trimethylbenzoylphosphinate (LAP), tris(2-carboxyethyl)phosphine hydrochloride (TCEP), and formamide were ordered from Meryer (Shanghai, China), while the deionized (DI) water (18.2 MΩ•cm) was obtained from the Milli-Q ICW3000 water system.

### Synthesis of Bismuth-Doped Carbon Dots (BiCD)

BiCD was synthesized using potassium bismuth citrate as the Bi source via a hydrothermal reaction. Initially, 0.5 g of anhydrous citric acid and 0.5 g of potassium bismuth citrate were completely dissolved in 50 mL of a cosolvent system comprising DI water and formamide (4:1, *v*/*v*) at room temperature to obtain a transparent solution. Then, the solution was placed into a 100 mL Teflon-lined hydrothermal autoclave, followed by heating at 180 °C for 4 h. After the reaction solution in the autoclave was cooled to room temperature, it was centrifuged at 8000 rpm for 5 min (Centrifuge 5804, Eppendorf, Hamburg, Germany) to collect the supernatant before being dialyzed against DI water (500 Da, Jiele Pu, China). After 2 days, the dialyzed solution was collected and lyophilized to harvest BiCD as a black powder.

### Conjugation of Amine-Modified Berberine

The amine-modified berberine (Ber-NH_2_) was synthesized from berberine through five-step reactions (Figure S1). For the conjugation with BiCD, 100 mg of BiCD was dispersed in DMF (20 mL), followed by the addition of 10 μL of distilled triethylamine. Then, EDC (53.3 mg) and NHS (30 mg) were dissolved in the reaction solution. After stirring for around 10 min, 20 mg of Ber-NH_2_ was added to the reaction under vigorous stirring. After 24 h, the reaction was diluted with DI water (40 mL) and dialyzed against water for 2 days (1000 Da, Jiele Pu, China). The final product was collected by lyophilizing the dialyzed solution and is denoted as BiCD-Ber.

### Characterization of BiCD and BiCD-Ber

The morphology of the as-synthesized BiCD was assessed using a Tecnai G2 20 S-TWIN transmission electron microscope (TEM, Thermo Fisher Scientific, Massachusetts, USA) equipped with an Energy Dispersive X-ray Spectrometer (EDX). The average size of BiCD was calculated from 40 particles selected from TEM images using ImageJ (Fiji, 2.14.0/1.54f, National Institutes of Health, USA). Regarding the crystalline properties, a Bruker D8 Advance X-ray diffractometer operating at 40 kV and 80 mA with Cu Kα (λ=1.5406 Å) was used. The functional groups and elemental composition were assessed using a Nicolet Magna 550 Series II Fourier transform infrared spectroscopy (FTIR, Nicolet Instrument, Madison) and a Thermo K-Alpha X-ray Photoelectron Spectrometer (XPS) System (Thermo Fisher Scientific, Waltham, USA), respectively. The ζ-potential of different CDs was analyzed by a DelsaMax PRO light scattering analyzer (Beckman Coulter, Brea, USA), and each sample was measured three times to obtain the mean value. For the fluorescent and optical properties, the fluorescent and UV–Vis absorption spectra of BiCD and BiCD-Ber were measured by a Horiba FluoroMax-4 Spectrofluorometer (FM-4, Kyoto, Japan) and an Agilent Cary 8454 UV–Vis spectrometer (Santa Clara, USA), respectively.

### Bacterial Strains and Growth Conditions

The bacterial strains, *P. gingivalis* (*Pg*, W83), *A. actinomycetemcomitans* (*Aa*, ATCC 29523), *F. nucleatum* (*Fn*, ATCC 25586), and *Streptococcus mutans* (*Sm*, ATCC 35668) were ordered from the American Type Culture Collection (ATCC, Manassas, USA). The bacteria were maintained on blood agar plates with 5% horse blood (Hemostat Laboratories, Dixon, CA, USA), 1% hemin, and vitamin K_1_ solution, and incubated in an anaerobic chamber at 37 °C. Before performing different biological assays, *Pg* and *Fn* were inoculated in supplemented tryptic soy broth (TSB, denoted as *Pg* broth), while *Aa* and *Sm* were cultured in brain heart infusion (BHI) broth. The microorganisms at the log phase were diluted to a certain concentration (OD_660_ = 0.1) for the biological assays.

### Cell Culture

The primary human gingival epithelial cells (hGEC) and culture medium (CnT-prime) were ordered from CELLnTEC (Zurich, Switzerland), while primary human gingival fibroblasts (pHGF) and fibroblast basal medium supplemented with a low serum fibroblast growth kit were obtained from ATCC. Both primary cells were cultured in their respective media with Primocin at 100 μg/mL (InvivoGen, San Diego, USA) and placed in a humidified incubator at 37 °C with 5% CO_2_. In general, the third to fifth passages of hGEC and pHGF were used for the subsequent biological experiments.

### Antimicrobial and Antibiofilm Activities of BiCD, Ber-NH_2_ and BiCD-Ber

The antimicrobial effects of BiCD, Ber-NH_2_. and BiCD-Ber were assessed by determining minimum inhibitory concentrations (MICs) for three selected periodontal pathogens (*Pg*, *Aa*, and *Fn*) using a microdilution assay. In general, different types of BiCDs or Ber-NH_2_ were dispersed or dissolved in distilled water to prepare the stock solution. These solutions were then added to a 96-well plate via a 2-fold serial dilution with the broth. Subsequently, the bacterial suspension (OD_660_ = 0.1) was transferred to the wells at a final concentration of 2 × 10^7^ CFU/mL. Following anaerobic incubation for 48 or 72 h, the OD_660_ values of the 96-well plates were measured using the SpectraMax M2 microplate reader (Molecular Devices, California, USA), and the turbidity degree in each well was recorded to calculate MIC values. After obtaining MIC values, the minimal bactericidal concentrations (MBCs) were evaluated by spotting 2 μL of aliquot from each well on blood agar plates, followed by the anaerobic incubation for 3–7 days until obvious bacterial colonies were observed. For assessing the morphology of *Pg* cells after different treatments, 5 mL of *Pg* at 2 × 10^7^ CFU/mL was treated with BiCD, Ber-NH_2_ and BiCD-Ber at sub-MIC concentrations for 24 h. Next, the bacteria were collected by centrifugation at 5000 rpm for 10 min and resuspended in 200 μL of normal saline. Prior to fixation with a 2.5% glutaraldehyde solution for 1 h at room temperature, 10 μL of each suspension was dropped onto 15 mm Thermonox plastic coverslips (Thermo Fisher Scientific, Waltham, USA) and air-dried. The well-fixed samples were washed with phosphate-buffered saline (PBS, pH 7.2–7.4) twice and dehydrated in a series of ethanol solutions (30, 50, 70, 85, 95, and 100%). Lastly, the morphologies of *Pg* under different treatments were examined using a Hitachi S-4800 field emission scanning electron microscope (FE-SEM; Hitachi Ltd., Tokyo, Japan).

Regarding the antibiofilm effects on the 3-day-old *Pg* biofilms, the bacteria at the log phase were diluted in broth to 2 × 10^8^ CFU/mL (OD_660_ = 0.1) and then added to a 96-well plate (100 μL/well) to incubate anaerobically and statically for 3 days. Afterward, the supernatants were discarded, and the biofilms were gently washed with PBS to remove the planktonic and loosely attached bacterial cells. Subsequently, BiCD, Ber-NH_2_, and BiCD-Ber at different concentrations were prepared in broth and added to the biofilms. Following a 24-h incubation, the treatments were removed, and the bacteria in the adhered biofilms were collected. The bacterial cells were then diluted to various concentrations and plated on blood agar before being incubated anaerobically to count visible colonies (CFU). Meanwhile, the antibiofilm activities were examined by using confocal scanning laser microscopy. The biofilm culturing and treatment were identical to the aforementioned procedures, except that the biofilms were cultured in ibidi GmbH μ-Slide 8-well chambers (Munich, Germany). After the treatments, the biofilms were stained with the Live/Dead BacLight viability kit (Thermo Fisher Scientific) for 30 min at room temperature, and the biofilm viabilities and thickness were assessed using an Olympus FLUOVIEW FV1000 confocal scanning laser microscope equipped with a 543 nm HeNe laser and a 488 nm Argon laser (Tokyo, Japan). The fluorescence images were analyzed using ImageJ (Fiji, 2.14.0/1.54f, National Institutes of Health, USA).

### Clearance of Extracellular and/or Intracellular Bacteria

pHGF and HGEC were first cultured in T75 flasks to reach 80% cell confluency. Then, the cells were detached by TrypLE Express Enzyme (1X) (Thermo Fisher Scientific) and seeded in 24-well plates at specific concentrations (1 × 10^5^ cells/well for pHGF and 5 × 10^5^ cells/well for HGEC). After overnight adherence, the cells were infected with *Pg* at a multiplicity of infection (MOI) of 100 for 3 h before being used to study the clearance of intracellular bacteria. In brief, the cells were initially treated with mixed antibiotics at a high concentration (200 μg/mL of MTZ and 300 μg/mL of GTM) for 1 h to eliminate the extracellular bacteria, followed by the treatment with MTZ (20 μg/mL) or BiCD, Ber-NH_2_, and BiCD-Ber (100 μg/mL) for 4 h to eradicate the intracellular bacteria. Finally, the host cells were gently washed with PBS, lysed in DI water for 30 min, and the cell lysates were diluted to proper concentrations and plated on blood agars for CFU counting. Referring to the investigation of both extra- and intracellular bacteria, the cells after *Pg* infection were directly treated with MTZ (20 μg/mL) or BiCD, Ber-NH_2_, and BiCD-Ber (100 μg/mL) for 6 h. After the treatment, the cells were lysed, and the cell lysates were diluted and plated as described above.

### Cell Imaging and Cellular Internalization of BiCD and BiCD-Ber

Both pHGF and HGEC were seeded in 8-well chambers and cultured for 3 days to reach confluency. Then, the cells were treated with BiCD, Ber-NH_2_, and BiCD-Ber at 50 μg/mL for 4 h, respectively. The cellular fluorescence was imaged using a Leica Microsystems DMi8 microscope with LED 8 and DFC9000 sCMOS fluorescence microscope and analyzed by LAS X Office (1.4.4, Leica, Wetzlar, Germany).

Regarding the cellular internalized nanomedicines, the amount was determined using a SPECTRO ARCOS Inductively coupled plasma optical emission spectroscope (ICP-OES, FHE12, AMETEK Inc., Berwyn, USA). In brief, pHGF and HGEC were seeded in 6-well plates with specific densities (1 × 10^5^ cells/well for pHGF and 2 × 10^5^ cells/well for HGEC) and cultured to reach confluency. Afterward, the cells were treated with BiCD and BiCD-Ber at 50 μg/mL for 2, 6, and 24 h. After being treated for a certain period of time, the cells were washed with Hank’s balanced salt solution (HBSS, Thermo Fisher Scientific) to wash the loosely attached nanomedicines. Then, the cells were lysed using 100 μL of M-PER mammalian protein extraction reagent (Thermo Fisher Scientific) and diluted with 900 μL of 10% HNO_3_. The collected cell lysates were centrifuged at 14,000 rpm for 5 min, and the supernatants were collected and diluted with DI water to 5 mL prior to the determination of Bi concentration by ICP-OES.

### Restoring *Pg*-Perturbed Host Immuno-Inflammatory Responses by the Nanomedicines

pHGF were seeded in a 24-well plate at 1 × 10^5^ cells/well for adherence overnight. On the second day, the cells were primed with IL-1β (1 ng/mL) for 6 h, followed by infection of *Pg* (MOI of 100) with or without treatments (MTZ at 20 μg/mL; BiCD, Ber-NH_2_, and BiCD-Ber at 50 μg/mL) for 18 h. After the treatments, the supernatants were collected for analyzing IL-6 and IL-8 levels using the ELISA kits ordered from R&D systems (Minneapolis, USA).

### Protect Against *Pg*-Mediated Disruption of Cell Attachment

HGECs were seeded in six-well plates at 1 × 10^6^ cells/well for adherence overnight. The following day, the cells were infected with *Pg* (MOI of 50) with or without treatments (MTZ at 20 μg/mL; BiCD, Ber-NH_2_, and BiCD-Ber at 50 μg/mL) for 24 h. The cells were then gently washed with ice-cold PBS twice and collected for the analysis of integrin β1 and E-cadherin. The protein concentration from each sample was determined using a Pierce BCA Protein Assay Kit (Thermo Fisher Scientific). Subsequently, an equivalent amount of the protein aliquots (20 μg) from different samples was loaded on and separated using 10% SDS-PAGE gels. The separated proteins were transferred to Amersham Hybond P Western blotting polyvinylidene fluoride membranes (GE Healthcare, Chicago, USA), followed by 1 h blocking in Pierce Protein-Free T20 Blocking Buffer (Thermo Fisher Scientific). The well-blocked membranes were incubated with the diluted (1:1000) rabbit monoclonal primary antibody of integrin β1 and E-cadherin (Cell Signaling Technology, Danvers, USA) at 4 °C overnight, respectively. After the incubation, the membranes were washed and incubated with the diluted (1:3000) secondary antibodies (Cell Signaling Technology) conjugated with horseradish peroxidase for 2 h. The blots were detected using a WesternBright Sirius Chemiluminescent Detection Kit (Advansta, San Jose, USA) and imaged by an Invitrogen iBright 1500 imaging system (Thermo Fisher Scientific).

For the immunofluorescent staining, the cells were seeded in μ-Slide 8-well chambers (ibidi) at 1 × 10^5^ cells/well followed by the same infections and treatments described above. After the treatment, the cells were fixed in 4% formaldehyde for 15 min at room temperature, followed by washing with PBS twice and permeabilization in PBS containing 0.1% Triton X-100 for 10 min. Afterward, the cells were blocked in a 10% normal goat serum solution (Life Technologies, Carlsbad, USA) for 1 h at room temperature and incubated with a rabbit monoclonal primary antibody of integrin β1 (Cell Signaling Technology) at a dilution rate of 1:100 at 4 °C overnight. Subsequently, the cells were washed with PBS twice and incubated with the diluted antirabbit IgG with Alexa Fluor 488 conjugate (1:500, Cell Signaling Technology) for 2 h at room temperature in the dark. Following this, the samples were washed with PBS again, stained with 5 μM of the DRAQ5 fluorescence probe, and examined using a Zeiss LSM900 with Airyscan 2 confocal microscope equipped with diode lasers of excitation wavelengths of 405, 488, 561, and 640 nm (Carl Zeiss NTS Ltd., Germany).

### Inhibitory Effects of BiCD, Ber-NH_2_, and BiCD-Ber on Amidolytic Activities of Recombinant RgpB_230–736_ and Kgp_229–595_, and *Pg* Cells

The amidolytic activities of RgpB_230–736_ and Kgp_229–595_ were determined using the fluorogenic substrate Z-Phe-Arg 7-amido-4-methylcoumarin hydrochloride (Z-FR-AMC; Sigma) and *N*-succinyl-Ala-Phe-Lys 7-amido-4-methylcoumarin acetate salt (Suc-AFK-AMC; Sigma) in assays performed in 96-well plates at 37 °C. Briefly, 200 μL reaction mixtures contained 50 mM Tris-HCl (pH 7.4), 150 mM NaCl, 5 mM CaCl_2_, 0.2 mM corresponding substrate, and 200 nM RgpB_230–736_ or Kgp_229–595_. A microplate reader (SpectraMax M2e Multimode Microplate Reader) was used to quantify the gingipain activities continuously by measuring AMC (release from substrates) fluorescence (λ_ex_ = 365 nm; λ_em_ = 465 nm).

*Pg* W83 was cultivated in *Pg* broth in anaerobic conditions to the late logarithmic phase and harvested via centrifugation at 5000*g* and 4 °C for 20 min. The resulting supernatant was carefully discarded, and the cell pellet was washed once with phosphate-buffered saline (PBS). Following another centrifugation, the cell pellet was resuspended in fresh precold PBS to a density of 1.0 × 10^9^ cells/mL, yielding washed *Pg* cells.

The inhibitory effects of BiCD, Ber-NH_2_ and BiCD-Ber on the amidolytic activities originating from purified RgpB_230–736_ or Kgp_229–595_ or *Pg* cell suspensions were assessed by the same assay as described above. Specifically, inhibition assays were performed with RgpB_230–736_ or Kgp_229–595_ or 10^6^ CFU/mL *Pg* washed cells, either in the absence or presence of varying concentrations (0–200 μg/mL) of BiCD, Ber-NH_2_, and BiCD-Ber. The inhibition was expressed as the percentage of the reaction rate at a given compound concentration relative to the reaction rate in the absence of the compound. Assays were performed in triplicate, with error bars shown alongside the mean value. The IC_50_ value was calculated using four-parameter logistic nonlinear regression models in GraphPad Prism 10.

### Synthesis and Characterization of Disulfide Bond-Modified Acrylated Hyaluronic Acid for Constructing Hydrogel via Photo-Cross-Linking

The disulfide bond-modified acrylated hyaluronic acid was synthesized from hyaluronic acid via a two-step reaction. In brief, 1 g of hyaluronic acid (HA) was dissolved in 50 mL of distilled water under vigorous stirring. Then, 2.7 g of cystamine dihydrochloride, 1.9 g of EDC, and 1.9 g of HNS were subsequently added to the solution, and the reaction was stirred at room temperature overnight. Next, the solution was transferred to a dialysis bag with a molecular weight cutoff (MWCO) of 12–14 KD (Standard RC Tubing, Spectrum Laboratories Inc., Rancho Dominguez, USA) and dialyzed against water for 3 days. The well-dialyzed disulfide bond-modified HA (HASSA) was freeze-dried and collected for the next-step reaction. Then, 0.25 g of HASSA was redissolved in 40 mL of D.I. water, followed by the addition of 1.2 g of *N*-succinimidyl acrylate under vigorous stirring. After an overnight reaction, the solution was centrifuged, and the viscous supernatant was dialyzed using the same dialysis bag with an MWCO of 12–14 KD against water for 3 days. The final product, with modifications of both the disulfide bond and acrylate groups, was lyophilized and denoted as HASSAC for hydrogel construction. The modifications were analyzed using proton nuclear magnetic resonance (^1^H NMR). HA, HASSA, and HASSAC were dissolved in deuterium oxide (D_2_O, Cambridge Isotope Laboratories, Tewksbury, USA), and their spectra were obtained using a 600 MHz Bruker Avance III spectrophotometer (Billerica, USA). Regarding the gelation, HASSAC and LAP were dissolved in sterilized PBS with final concentrations of 1.5% and 0.25% (*w*/*v*), respectively. The gelation was initiated via UV irradiation at 405 nm for 30 s. After the lyophilization, the porosity of the hydrogel was evaluated by a Hitachi SU1510 scanning electron microscope (Tokyo, Japan).

### Disulfide Bond Cleavage-Based *Pg*-Responsive Hydrogel Degradation

For a more obvious observation of hydrogel degradation, 20 μL of hydrogel working solution (HASSAC 1.5% and LAP 0.25% in PBS), mixed with rhodamine 123 (red dye), was photo-cross-linked in the confocal dish. Two reducing agents for disulfide bond cleavage, TCEP and DTT, were dissolved in PBS at 20 mM and added to the cross-linked hydrogels. The solution was changed daily, and the morphology of the hydrogel was recorded. For pathogen-responsive degradation, the hydrogels were prepared as described above. In general, 3 mL of *Pg* or *Sm* suspension with an OD_660_ value of 0.1 was added to the hydrogel-containing confocal dishes and incubated anaerobically. The hydrogels incubated with blank culture media (*Pg* broth or BHI) were considered as controls. The morphology of hydrogels was recorded from Day 0 to Day 6. Meanwhile, the hydrogel working solution mixed with BiCD-Ber, with a final concentration of 5 mg/mL, was also cross-linked in the confocal dish, and its degradation was compared with the blank hydrogel with *Pg* incubation. The gel morphologies were recorded for 7 days.

### In Vitro Anti-*Pg* Effects of Hydrogel-Encapsulated BiCD-Ber and Its Releasing Profile

20 μL of the hydrogel working solution, mixed with different amounts of BiCD-Ber (final concentrations: 5, 2, 1, and 0 mg/mL), was cross-linked in a 96-well plate and incubated with 100 μL of *Pg* suspension (OD_660_ = 0.1, around 2 × 10^8^ CFU/mL) anaerobically for 3 days. After the incubation, 50 μL of the cocultured bacterial suspension was plated on blood agars for examining the anti-*Pg* effect. For the releasing profiles, 1 mL of hydrogel working solution with BiCD-Ber at a final concentration of 2 mg/mL was cross-linked in each well of a 6-well plate. The release of BiCD-Ber was evaluated in both disulfide-bond cleavage and normal modes. In brief, TCEP solution at 5 mM was prepared in PBS and added to the hydrogels for testing the release of BiCD-Ber under the disulfide-bond cleavage-based hydrogel degradation. Meanwhile, blank PBS was also added to other hydrogels to study the releasing BiCD-Ber at normal mode. At each time point, the supernatant was collected, and fresh TCEP or PBS solution was added to the hydrogels. The released BiCD-Ber was measured by determining the bismuth amount using ICP-OES.

### In Vivo Evaluation of BiCD-Ber HA Gel on Experimental Periodontitis in Rats

The female Sprague–Dawley rats (MGI:5651135), weighing 200–220 g, were purchased from the Laboratory Animal Center of Sun Yat-sen University, Guangzhou, China. They were housed in a specific pathogen-free facility with access to food and water, and maintained in a temperature-controlled laboratory with a 12-h light/12-h dark cycle. Prior to the experimental periodontitis induction, all rats underwent a 1-week acclimatization period. The experimental procedures were approved by the Ethics Committee for Animal Experiments of Sun Yat-sen University (No. SYSU-IACUC-2023–001853).

For grouping and treatment, 20 Sprague–Dawley rats were randomly allocated into 4 groups (*n* = 5 in each group): the control group, periodontitis group, HA gel group, and BiCD-Ber HA gel group. In the experimental groups, 4/0 braided silk (JinHuan, China) was firmly tied subgingivally around the bilateral maxillary second molars of the rats while they were anesthetized with pentobarbital sodium (40 mg/kg, i.p.). The ligatures were inspected every 2 days, and at the same time, *Pg* (W83, 1 × 10^9^ CFU/mL) was inoculated into the gingival sulcus using a syringe. HA gel and BiCD-Ber HA gel were injected into the gingival sulcus of the respective groups. Three weeks after treatment, the gingival crevicular fluid from the maxillary second molars was collected. Subsequently, all rats were euthanized, and the maxillae were extracted and fixed in 4% paraformaldehyde for 48 h for subsequent analysis.

The extracted maxillae were scanned using micro-CT (SkyScan 1276, Bruker, Germany) at a resolution of 15 μm, a voltage of 85 kV, and a current of 200 μA. The samples were aligned using Dataviewer software (v.1.5.6.2), whereas the bone volume (BV, μm^3^), tissue volume (TV, mm^3^), and BV/TV (%) of bone around the maxillary second molar were calculated using CTAn software (v.1.20). Additionally, the distances from the cemento-enamel junction to the alveolar bone crest (CEJ-ABC, μm) of the maxillary second molars were measured. For histological analysis, the samples were decalcified in 10% EDTA solution at 4 °C for 8 weeks followed by dehydration and wax leaching. After embedding in wax, the tissues were sectioned at a thickness of 4 μm and mounted on glass slides. After dewaxing and rehydration, the sections were stained with hematoxylin and eosin (HandE) using a kit ordered from Servicebio (China). The well-stained sections were sealed with neutral gum and imaged with Aperio slide scanner (Leica Biosystems Aperio, USA) to evaluate the absorption of alveolar bone around the maxillary second molars (CEJ-ABC). The acquired images were analyzed using ImageJ. To assess the number of osteoclasts around the second molar, tartrate-resistant acid phosphatase (TRAP) staining was performed on the tissue sections. The sections were counterstained with hematoxylin. Multinucleated TRAP-positive cells on the surface of the alveolar bone around the second molar were identified and counted as active osteoclasts. Meanwhile, immunohistochemical techniques were used to evaluate the expression of inflammatory factors around the second molar. The tissue sections were dewaxed, rehydrated, and subjected to antigen retrieval. The sections were then treated with 3% hydrogen peroxide for 25 min to block endogenous peroxidase activity, and 3% BSA was used for 30 min to block nonspecific binding. Diluted primary antibodies against interleukin (IL)-1β (Servicebio, China, 1:50 dilution), IL-6 (Servicebio, China, 1:400 dilution), and IL-10 (Servicebio, China, 1:400 dilution) were incubated overnight at 4 °C. Subsequently, the tissue sections were incubated with antirabbit IgG secondary antibodies for 50 min at room temperature. The chromogenic reaction was performed using 3,3′-diaminobenzidine tetrahydrochloride (DAB, Servicebio, China) as the chromogen substrate, and counterstaining was performed with hematoxylin (Servicebio, China). Images were captured with an Aperio slide scanner (Leica Biosystems Aperio, USA). The region of interest (ROI) around the second molar was captured randomly using Aperio ImageScope (Leica, Germany).

### Sequencing Sample Collection, DNA Extractions, Sequencing, and Data Analysis

Prior to sampling, the clinical sites were first isolated and dried with sterile cotton rolls. Gingival crevicular fluid was acquired from the buccal and lingual regions of the maxillary second molars of rats using extra-fine paper points. The paper points were placed into sterile, empty tubes and stored individually at −80 °C until further analysis. Subgingival bacterial genomic DNA was extracted using the MagPure Stool DNA KF kit B (Magen, China) according to the manufacturer’s instructions. Polymerase chain reaction (PCR) amplification of the hypervariable V3–V4 region of 16S rRNA genes was performed using the universal bacterial primer pairs 338F (5′-ACTCCTACGGGAGGCAGCAG-3′) and 806R (5′-GGACTACHVGGGTWTCTAAT-3′). Paired-end sequencing was performed on the amplicon libraries using the DNB MGI-2000 platform (BGI, Shenzhen, China). The paired forward and reverse sequence reads were assembled, followed by the filtering of noisy sequences and chimera checking, whereas ASV sequences were generated by clustering denoised sequences with 100% similarity using DADA2 (Divisive Amplicon Denoising Algorithm) in the QIIME2 software. Taxonomy assignments were made against the Silva database (V138 2019-12-16). The structure of different peri-implant microbial communities, the relative abundance, alpha diversity, and beta-diversity were estimated using Mothur and Qiime. Beta diversity comparisons were performed by weighted and unweighted UniFrac analyses, which were visualized in principal coordinate analysis (PCoA) plots. The comparisons of the relative abundance among different groups were determined by the R package based on the Kruskal-Test. Differentially abundant taxa were identified with linear discriminant analysis (LDA) effect size (LEfSe). The *p*-value threshold was set to 0.05.

### Statistical Analysis and Data Depository

The data obtained from *in vitro* assays were statistically analyzed by one-way analysis of variance (ANOVA) with Bonferroni’s multiple comparisons test. Regarding the *in vivo* experiments, the data were analyzed by one-way ANOVA with multiple comparisons of Fisher’s LSD test. All of the results were plotted using GraphPad Prism 10. The sequences and metadata were deposited in the NCBI Short Read Archive under the accession of biosamples: PRJNA1150153.

## Supporting Information Available

Synthesis and characterization of amine-modified berberine (NMR and MS spectra); EDX analysis and size distribution of BiCD; UV–vis and fluorescent spectra of Ber-NH_2_; checkerboard analysis of BiCD and Ber-NH_2_ on *Pg*; cytotoxicity assessment of as-synthesized nanomedicines and Ber-NH_2_ on pHGF and HGEC cells; immunofluorescent staining of *Pg*-infected pHGF and HGECs; immunofluorescent staining of integrin β1 and F-actin in HGECs infected by *Pg* with or without treatments; storage modulus (G’) and loss modulus (G’’) of hydrogels constructed at 1.5% and 2.0% HASSAC; H&E staining of vital organs collected from the rats after different treatments; LEfSe analysis of periodontal microbiota after treatments; and PCR primers used in this study (PDF)
