# Supplementary material for: Berberine-Functionalized Bismuth-Doped Carbon Dots in a Pathogen-Responsive Hydrogel System: A Multifaceted Approach to Combating Periodontal Diseases
Source: ACS Nano. 2025 May 2;19(18):17554–77. doi: 10.1021/acsnano.5c00561 (PMC12080333; doi:10.1021/acsnano.5c00561)
Supplement: Supplementary file 1 — nn5c00561_si_001.pdf [file nn5c00561_si_001.pdf]

Supporting Information

Berberine functionalized bismuth-doped carbon dots in a pathogen-responsive hydrogel system: a multifaceted approach to combating periodontal diseases

Xuan Li[1], Regina Huang[1], Pugeng Li[2], Fung Kit Tang[1], Jing He[1], Hanyu Sun[2], Xiaoyu Wang[2], Miao Wang[1], Xinmiao Lan[3], Xinna Wang[4], Sarah Sze Wah Wong[5], Lijian Jin[1], Ken Cham-Fai Leung[6], Hai Ming Wong[1], Sheng Wang[7], Lanping Guo[7], Pei-Hui Ding[8] and Xiaolin Yu*[2]*

[1]Faculty of Dentistry, The University of Hong Kong, Hong Kong SAR, P. R. China.

[2]Hospital of Stomatology, Guanghua School of Stomatology, Guangdong Provincial Key Laboratory of Stomatology, Sun Yat-Sen University, Guangzhou, 510055, P. R. China.

[3]Beijing Area Major Laboratory of Peptide and Small Molecular Drugs, Engineering Research Centre of Ministry of Education of China, Beijing Laboratory of Biomedical Materials, School of Pharmaceutical Science, Capital Medical University, Beijing, 100069, P. R. China.

[4]Department of Mechanical Engineering, The University of Hong Kong, Hong Kong SAR, P. R. China.

[5]Immunology of Fungal Infections Unit, Institut Pasteur, Paris, 75015, France.

[6]Department of Chemistry, The Hong Kong Baptist University, Hong Kong SAR, P. R. China.

[7]State Key Laboratory for Quality Ensurance and Sustainable Use of Dao-di Herbs, National Resource Center for Chinese Materia Medica, China Academy of Chinese Medical Sciences, Beijing, 100700, P. R. China.

[8]Stomatology Hospital, School of Stomatology, Zhejiang University School of Medicine, Hangzhou, 310006, P. R. China.

Materials and methods

Materials. Berberine (97%), methyl bromoacetate, hexafluorophosphate azabenzotriazole tetramethyl uronium (HATU) and *N*-Boc-ethylenediamine were ordered from Meryer (Shanghai, China), while sodium hydroxide, acetonitrile (MeCN), methylene chloride (CH_2Cl_2) and triethylamine (NEt_3) were purchased from Dieckmann (Shenzhen, China).

***Characterization of berberine derivatives*.** Nuclear magnetic resonance (NMR) spectra were recorded from Bruker Advance–III 400 NMR spectrometer operating at 400 MHz for ^{1}H and 101 MHz for $^{13}C\{^{1}H\}$, respectively. Chemical shifts are reported in ppm. ^{1}H and ^{13}C chemical shifts were referenced internally with solvent residue chemical shift values (($(CD_3)_2SO$: ^{1}H, 2.50 ppm; ^{13}C, 39.52 ppm). NMR spectral data were processed using MestReNova Software (Mestrelab). High-resolution mass spectra were recorded on a Bruker Autoflex mass spectrometer (MALDI–TOF).

***Checkerboard assay of BiCD and berberine*.** In brief, BiCD and berberine were separately diluted in two plates. For plate A, BiCD were serially diluted by *Pg* broth with the highest concentration of 1 mg/mL. At the same time, berberine also had serial dilutions by *Pg* broth with the highest concentration of 1 mg/mL in plate B. Afterwards, 45 μL of each dilution from BiCD or berberine were mixed with 10 μL of *Pg* suspension at OD_{660}=0.1 and cultured anaerobically for three days to record the OD value in each well to determine the MIC values of pure BiCD or berberine FIC, and calculate the fractional inhibitory concentration (FIC) index value.

***Cytotoxicity of BiCD, Ber-NH$_2$ and BiCD-Ber*.** pHGF and HGECs were seeded in the 96 well plates at certain density (1×10^4 for pHGF and 2×10^4 for HGECs) and cultured for two days to reach 80% confluency, respectively. BiCD, Ber-NH_2 and BiCD-Ber at different concentrations (500, 250, 125, 62.5 and 31.25 μg/mL) were then added to the cells and incubated for 24 h. After the treatment, the supernatants were collected for measuring the concentration of cytosolic enzymes, lactate dehydrogenase (LDH), to assess the cell membrane integrity using CyQUANT LDH Cytotoxicity Assay Kit (Thermo Fisher Scientific), while the cells were incubated with 100 μL of culture media and 10 μL of CCK-8 reagent (Dojindo Laboratories, Kumamoto, Japan) in each well for 1 h. The absorption was measured at 450 nm in the SpectraMax M2 Microplate Reader (Molecular Devices, California, USA).

Immunofluorescent staining of Pg-infected host cells after different treatments. For the clearance of extracellular and/or intracellular bacteria, after the treatments illustrated in Figure 3A, immunofluorescence staining (IFS) was performed to indicate the amount and location of *Pg* after different treatments. The cells were firstly seeded in ibidi GmbH μ-Slide 8-well chambers with specific concentrations (1×10^4 cells/well for pHGF and 5×10^4 cells/well for HGEC), and then the cells had the same *Pg* infection and treatments as stated above. After the treatment, the cells were fixed using 4% paraformaldehyde at room temperature for 15 min followed by rinsing in PBS twice. Afterward, the cells were permeabilized in 0.1% Triton X-100 PBS solution and blocked in 10% normal goat serum solution (Life technologies, Carlsbad, USA) for 1 h. *Pg* were labelled with mouse anti-*P. gingivalis* primary antibody (Developmental Studies Hybridoma Bank [DSHB] hybridoma product 60BG1.3) followed by Alexa Fluor 488-

conjugated anti-mouse IgG secondary antibody (Cell Signaling Technology, Danvers, USA). The host cells were stained with Rhodamine Phalloidin (Thermo Fisher), and their nuclei were stained with DRAQ5 fluorescence probe (Thermo Fisher Scientific). The fluorescence images were acquired using Olympus FLUOVIEW FV 1000 confocal scanning laser microscope equipped with 543 nm HeNe laser, 488 nm Argon laser and 635 nm diode laser (Tokyo, Japan) and processed using ImageJ (Fiji, 2.14.0/1.54f, National Institutes of Health, USA).

Cloning, expression and purification of recombinant proteins. The nucleotide sequence encoding $RgpB_{230-736}$ and $Kgp_{229-595}$ were PCR amplified from *P. gingivalis* W83 strain genomic DNA and cloned into pET28a (Novagen, Merck Millipore) *via* EcoRI/XhoI or BamHI/XhoI to create plasmids pET28a-$RgpB_{230-736}$ and pET28a-$Kgp_{229-595}$, respectively. The plasmid detail and primer sequence are shown in Table S1. Plasmid integrity was confirmed by Sanger sequencing.

Recombinant plasmids containing PCR inserts with the correct DNA sequences were transformed into *Escherichia coli* BL21 (DE3) for protein expression. 5 mL overnight cultures that were originally inoculated from single colonies on LB agar plates, were expanded into 500 mL LB broth supplemented with 50 μg/mL kanamycin and incubated at 37°C for approximately 2-3 h with shaking at 200 rpm. When the OD600 reached *ca.* 0.4-0.6, protein expression was induced by the addition of 0.2 mM isopropyl β-D-1-thiogalactopyranoside (IPTG; GE Healthcare) and cultures were further incubated at 25°C with shaking at 200 rpm for 12 h. Cells were pelleted by centrifugation at 6000 × g, 4°C, 10 min, and were washed once with 30 mL pre-chilled phosphate-buffered saline (PBS; pH 7.4), then were frozen at -70°C for storage until protein purification later or immediately.

Cell pellets were thawed and resuspended in 30 mL pre-chilled Ni^{2+} binding buffer (20 mM imidazole, 25 mM Tris-HCl pH 7.4, 500 mM NaCl) in a 50 mL Falcon tube. The cells were lysed by pulsed sonication with ice cooling (Vibra Cell, Sonics & Materials Inc.; 36% amplitude, 2 s on, 8 s off, 60 min total). The cell lysate was centrifuged at 13000 × g and 4°C for 45 min. The supernatant was filtered by a 0.45 μm syringe filter. The filtered cell lysate underwent immobilized metal affinity chromatography (IMAC) on a pre-equilibrated 5 mL HiTrap chelating HP column (GE Healthcare, Chicago, USA) impregnated with nickel ions, using an ÄKTA purifier system (GE Health care), at a flow rate of 1 mL/min (monitoring the eluent at 280 nm). After loading the filtered cell lysate, the column was washed with 5 column volumes (CV) of Ni-binding buffer. Expressed recombinant protein was eluted by linear gradient elution of Ni-binding and Ni-elution buffer (250 mM imidazole, 25 mM Tris-HCl pH 7.4, 500 mM NaCl) over 20 CVs. Eluted fractions were routinely analyzed by SDS−PAGE acrylamide/bis-acrylamide gels (Bio-Rad, Hercules, USA). Protein concentrations were determined using the BCA Protein Assay Kit (Thermo Fisher Scientific).

Rheological analysis. The hydrogels were constructed from different HASSAC solutions (*w*/*v*, 1.5% and 2%), and their rheological properties were evaluated by the rheometer (MCR302, Anton Paar, Graz, Austria) under different settings. The variation of the storage modulus (G′) and loss modulus (G″) were examined by placing the prepared hydrogels (1.5% and 2% HASSAC) between parallel plates (25 mm diameter, 0.55 mm gap). Dynamic

frequency scan test was conducted at 37 °C with angular frequency ranging from 0.1 to 100 rad/s and a consistent strain of 1%. The viscosity of hydrogels was measured across shear rates ranging from 0.1 to 100 s^{-1}at 37 °C.

Synthesis

General Scheme

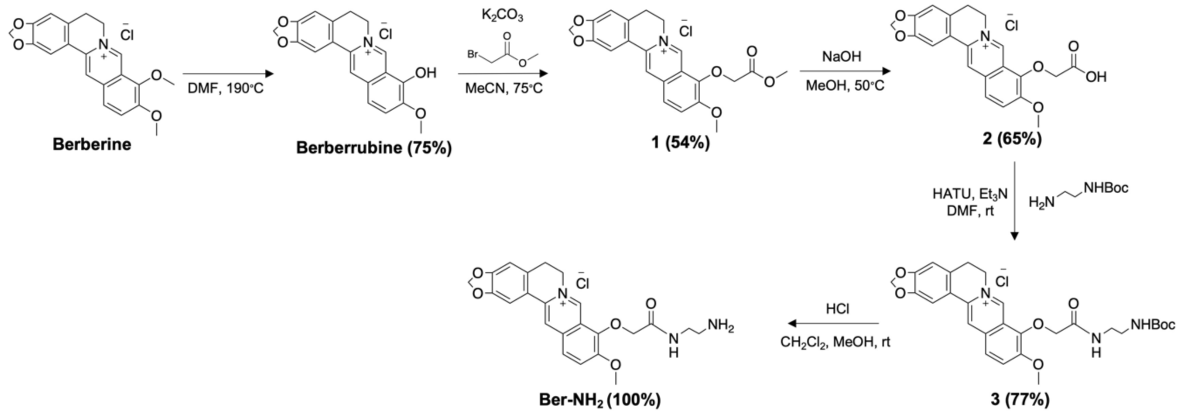

Figure S1 Synthetic scheme of Ber-NH_2.

Berberrubine. Berberine (1.17 g) was dissolved in 160 mL DMF and reflux at 190°C for 2 h. After the reaction, DMF in the solution was removed *via* rotary evaporation. The resultant red compound was dissolved and purified using silica gel with eluent of $CHCl_3$ and MeOH with ratio of 10:1 to afford berberrubine as dark red powder. Yield: 0.838 g, 75%. 1H NMR (400 MHz, $(CD_3)_2SO$, 298K) δ 9.07 (s, 1H), 7.98 (s, 1H), 7.62 (s, 1H), 7.20 (d, J = 7.9 Hz, 1H), 6.96 (s, 1H), 6.34 (d, J = 7.8 Hz, 1H), 6.10 (s, 2H), 4.48 (t, J = 6.1 Hz, 2H), 3.72 (s, 3H), 3.04 (t, J = 6.1 Hz, 2H), O*H* signal missing (Figure S2).

Compound 1. A mixture of **berberrubine** (2.32 g, 6.5 mmol), K_2CO_3 (10 g, 72 mmol) and methyl bromoacetate (7 g, 46 mmol) in 100 mL MeCN was stirred at room temperature for 4 h. Insoluble materials were filtered, the filtrate was collected and concentrated. The product was purified by silica gel chromatography using CH_2Cl_2/MeOH (*v*/*v* = 10:1) to afford **compound 1** as yellow powder. Yield: 1.51 g, 54 %. ^{1}H NMR (400 MHz, $(CD_3)_2SO$, 298K) δ 9.94 (s, 1H), 8.94 (s, 1H), 8.20 (d, *J* = 9.2 Hz, 1H), 7.99 (d, *J* = 9.0 Hz, 1H), 7.80 (s, 1H), 7.10 (s, 1H), 6.18 (s, 2H), 5.07 (s, 2H), 4.94 (t, *J* = 6.3 Hz, 2H), 4.03 (s, 3H), 3.72 (s, 3H), 3.21 (t, *J* = 6.4 Hz, 2H) (Figure S3). ^{13}C NMR (101 MHz, $(CD_3)_2SO$, 298K) δ 169.33, 149.87, 149.27, 147.69, 145.72, 141.46, 137.58, 132.93, 130.69, 126.74, 123.56, 121.16, 120.40, 120.09,

108.44, 105.45, 102.10, 69.25, 57.19, 55.42, 51.89, 26.37 (Figure S4). HRMS (MADLI–TOF): calculated for $C_{22}H_{20}NO_6^+$ $[M]^+$ *m/z* 394.1285, found 394.0598 (Figure S5).

Compound 2. **Compound 1** (1.51 g, 3.51 mmol) was dissolved in 2% NaOH in MeOH (1 g of NaOH in 50 mL MeOH). The mixture was heated at 60°C for 2 h. The solvent was removed by a rotary evaporator and the residue was acidified by 6 M HCl to around pH 2. The mixture was cooled in an ice bath and the yellow precipitate was collected by Buchner funnel filtering. The solid was rinsed with cold water and **compound 2** was obtained after air dry as yellow powder. Yield: 0.95 g, 65%. ^{1}H NMR (400 MHz, $(CD_3)_2SO$, 298K, –COO*H* proton signal is missing) δ 9.96 (s, 1H), 8.95 (s, 1H), 8.20 (d, J = 9.2 Hz, 1H), 7.98 (d, J = 8.9 Hz, 1H), 7.80 (s, 1H), 7.10 (s, 1H), 6.18 (s, 2H), 4.98 (s, 2H), 4.93 (t, J = 6.4 Hz, 2H), 4.04 (s, 3H), 3.21 (t, J = 6.3 Hz, 2H) (Figure S6). ^{13}C NMR (101 MHz, $(CD_3)_2SO$, 298K) δ 170.21, 149.86, 149.27, 147.70, 145.90, 141.73, 137.51, 132.95, 130.68, 126.80, 123.31, 121.25, 120.44, 120.08, 108.45, 105.47, 102.10, 69.16, 57.20, 55.41, 26.39 (Figure S7). HRMS: calculated for $C_{21}H_{18}NO_6^+$ $[M]^+$ *m/z* 380.1129, found 380.0815 (Figure S8).

Compound 3. **Compound 2** (40 mg, 0.1 mmol), HATU (55 mg, 0.14 mmol) and NEt_3 (14 µL, 0.1 mmol) and *N*-Boc-ethylenediamine (24 µL, 0.15 mmol) were dissolved in 8 mL DMF. The mixture was stirred at room temperature overnight. The solvent was removed by a rotary evaporator and the product was purified by silica gel column chromatography using CH_2Cl_2/MeOH (*v/v* = 10:1) to afford **compound 3** as yellow solid. Yield: 41.1 mg, 77%. ^{1}H NMR (400 MHz, $(CD_3)_2SO$, 298K) δ 10.00 (s, 1H), 8.93 (s, 1H), 8.27 (t, J = 5.5 Hz, 1H), 8.21 (d, J = 9.2 Hz, 1H), 8.00 (d, J = 9.1 Hz, 1H), 7.80 (s, 1H), 7.11 (s, 1H), 6.90 (t, J = 5.6 Hz, 1H), 6.18 (s, 2H), 4.93 (t, J = 6.4 Hz, 2H), 4.77 (s, 2H), 4.05 (s, 3H), 3.2 – 3.15 (m, 4H), 3.14 – 3.00 (m, 2H), 1.33 (s, 9H) (Figure S9). ^{13}C NMR (101 MHz, $(CD_3)_2SO$, 298K) δ 167.89, 155.77, 149.90, 149.85, 147.73, 145.91, 141.98, 137.55, 132.91, 130.66, 126.66, 123.77, 121.24, 120.45, 120.12, 108.48, 105.45, 102.13, 77.74, 71.71, 57.14, 55.47, 39.57, 38.75, 28.19, 26.41 (Figure S10). HRMS: calculated for $C_{28}H_{32}N_3O_7^+$ $[M]^+$ *m/z* 522.2235, found 522.2278 (Figure S11).

Ber-NH$_2$. **Compound 3** (31.6 mg, 0.06 mmol) was dissolved in 9 mL mixture of solvent 37% HCl: CH_2Cl_2: MeOH (*v/v/v* = 3:2:4 mL/mL/mL). The reaction mixture was stirred overnight. The solvent was removed by a rotary evaporator and the product was dried in vacuum as yellow solid. The final product was further recrystallized in MeOH with diethyl ether to give a yellow solid. Yield: 29 mg. ^{1}H NMR (400 MHz, $(CD_3)_2SO$, 298K) δ 10.25 (s, 1H), 8.96 (s, 1H), 8.67

(t, J = 5.6 Hz, 1H), 8.22 (d, J = 9.2 Hz, 1H), 8.18 – 8.09 (m, 2H), 8.02 (d, J = 9.1 Hz, 1H), 7.81 (s, 1H), 7.11 (s, 1H), 6.18 (s, 2H), 5.05 – 4.92 (m, 2H), 4.80 (s, 2H), 4.07 (s, 3H), 3.26 – 3.17 (m, 3H), 3.10 – 3.04 (m, 1H), 3.01 – 2.92 (m, 2H) (Figure S12). ^{13}C NMR (101 MHz, $(CD_3)_2SO$, 298K) δ 168.43, 150.02, 149.89, 147.74, 146.13, 141.70, 137.54, 132.93, 130.71, 126.63, 123.95, 121.16, 120.49, 120.11, 108.52, 105.49, 102.15, 71.61, 57.18, 55.19, 38.57, 36.30, 26.40 (Figure S13). HRMS: calculated for $C_{23}H_{24}N_3O_5^+$ $[M]^+$ m/z 422.1719, found 422.1710 (Figure S14).

NMR, mass and UV-Vis spectra

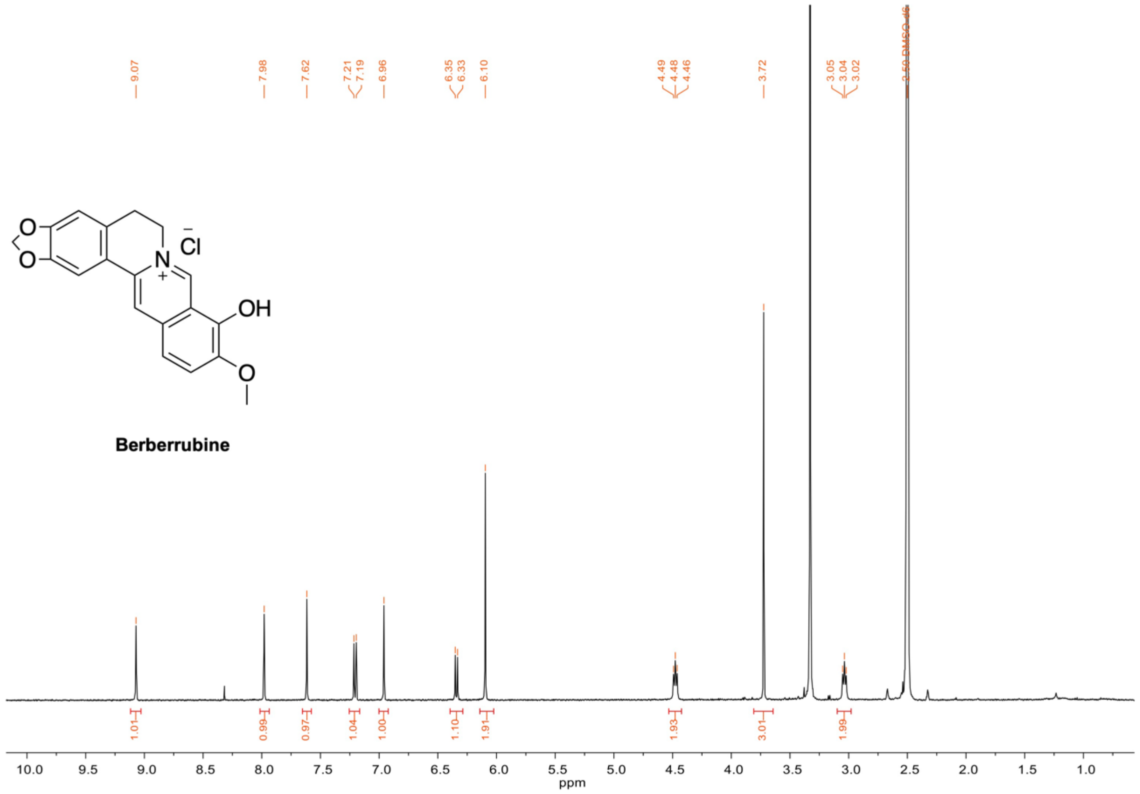

Figure S2 ^{1}H NMR (400 MHz, $(CD_3)_2SO$, 298K) of berberrubine.

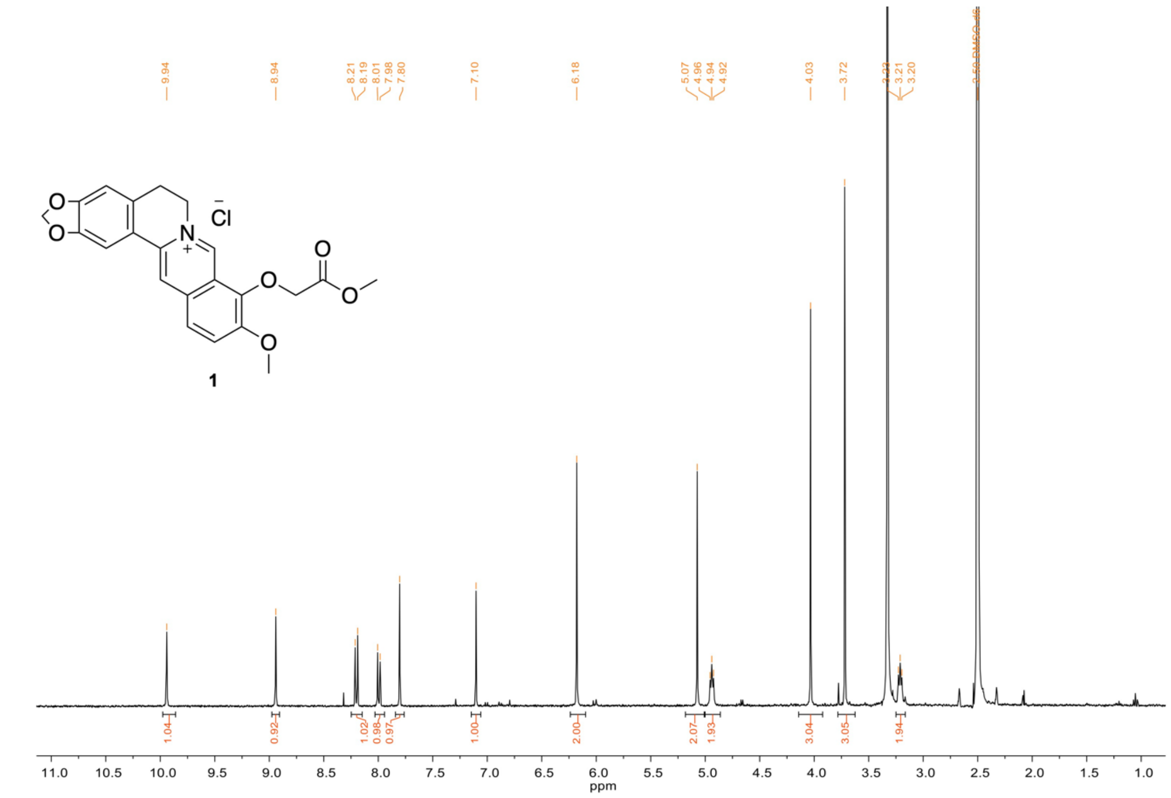

Figure S3 ^{1}H NMR (400 MHz, $(CD_3)_2SO$, 298K) of **compound 1**.

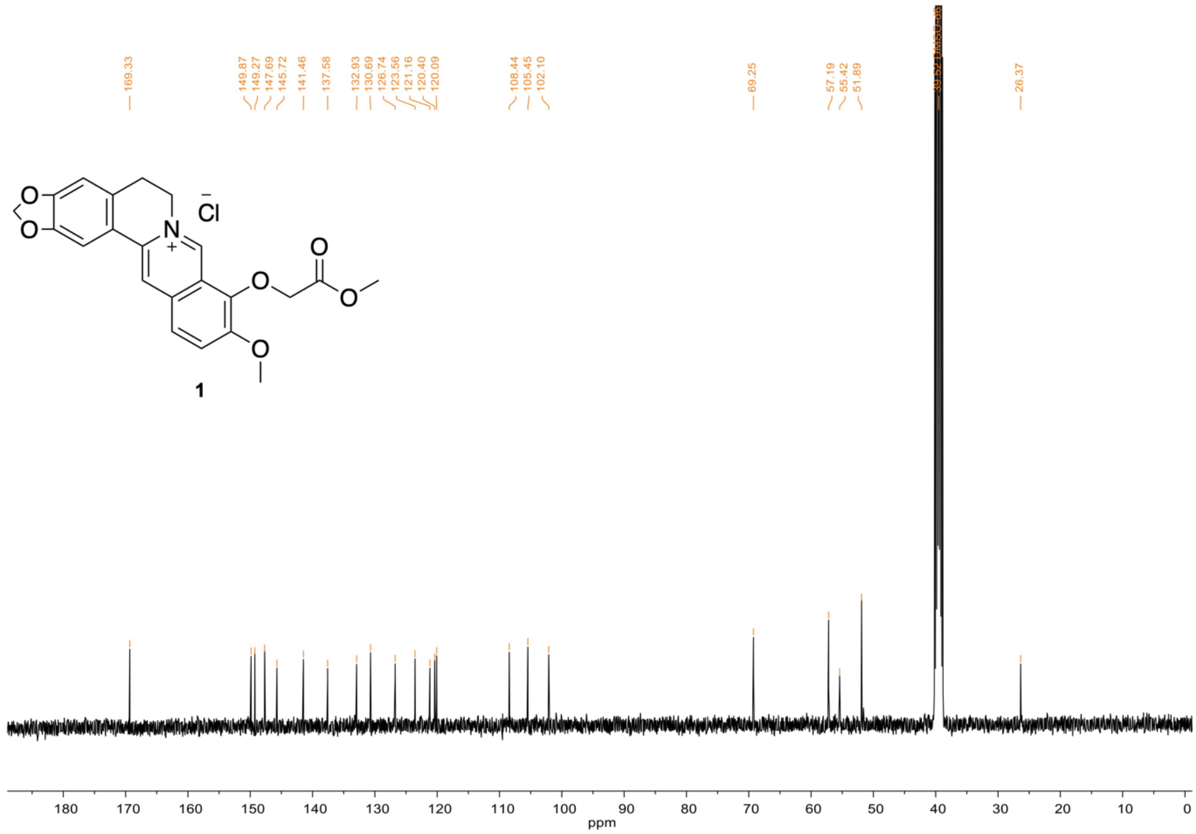

Figure S4 ^{13}C NMR (101 MHz, $(CD_3)_2SO$, 298K) of **compound 1**.

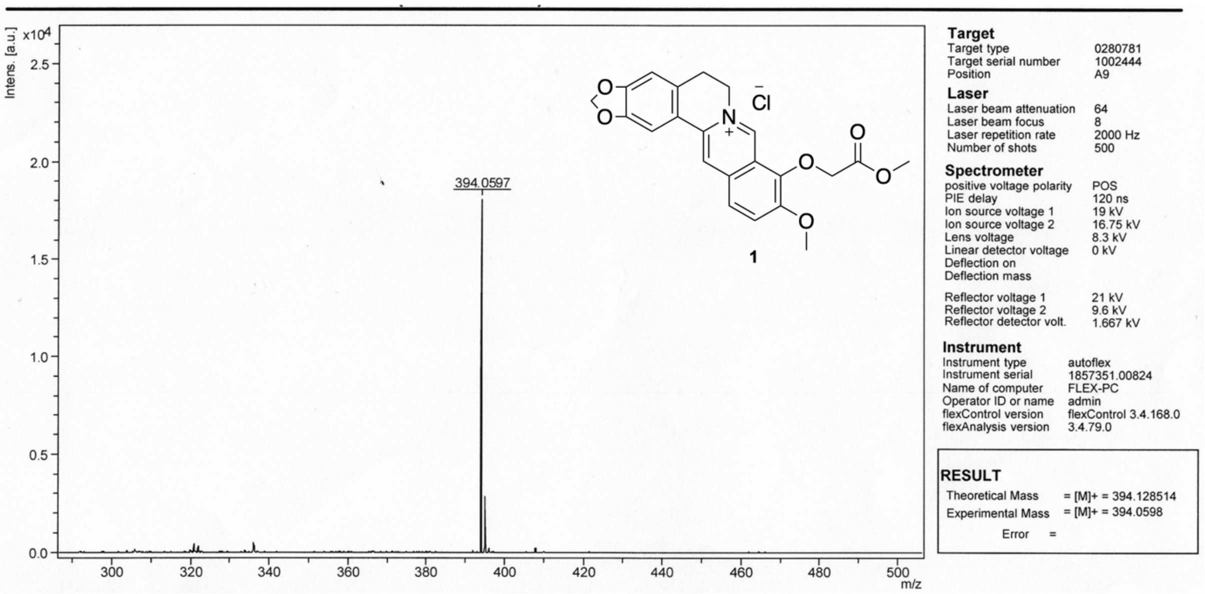

Figure S5 HRMS (MADLI–TOF) analysis of **compound 1**: calculated for $C_{22}H_{20}NO_6^+$ $[M]^+$ *m/z* 394.1285, found 394.0598.

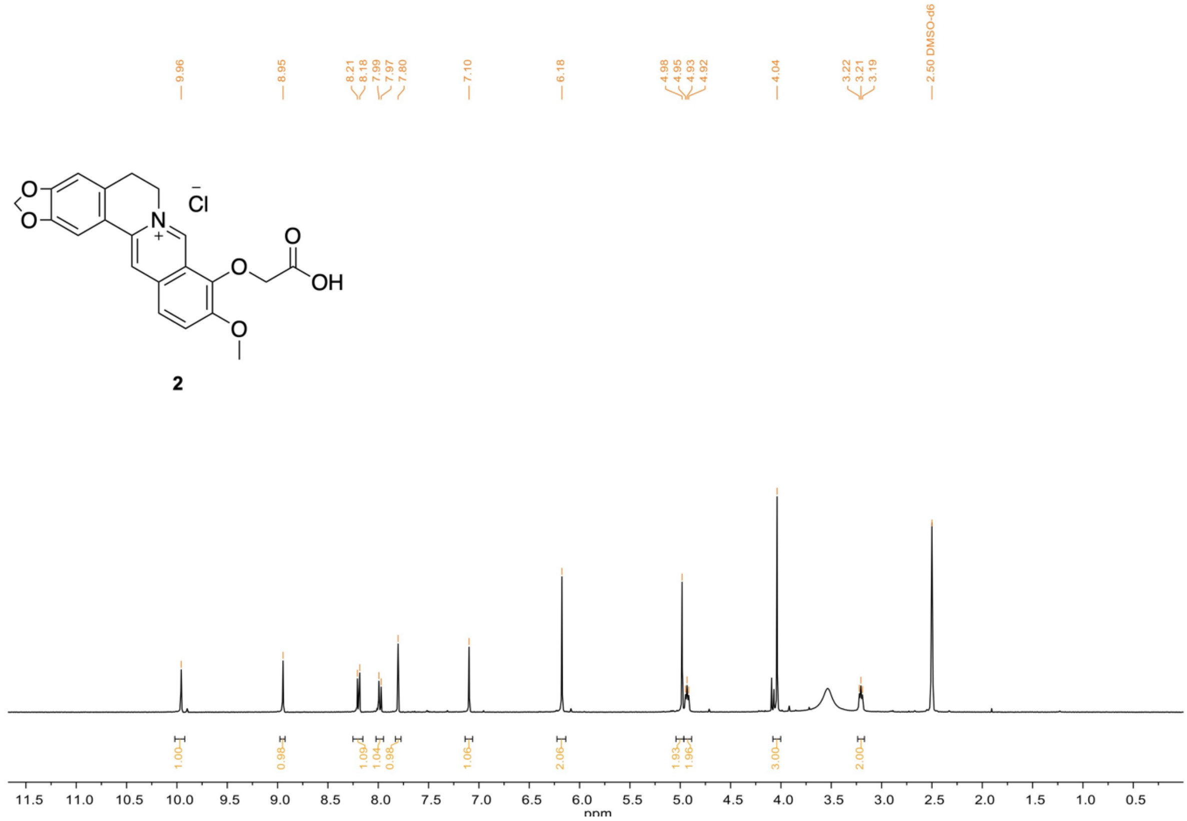

Figure S6 ^1H NMR (400 MHz, $(CD_3)_2SO$, 298K) of **compound 2**.

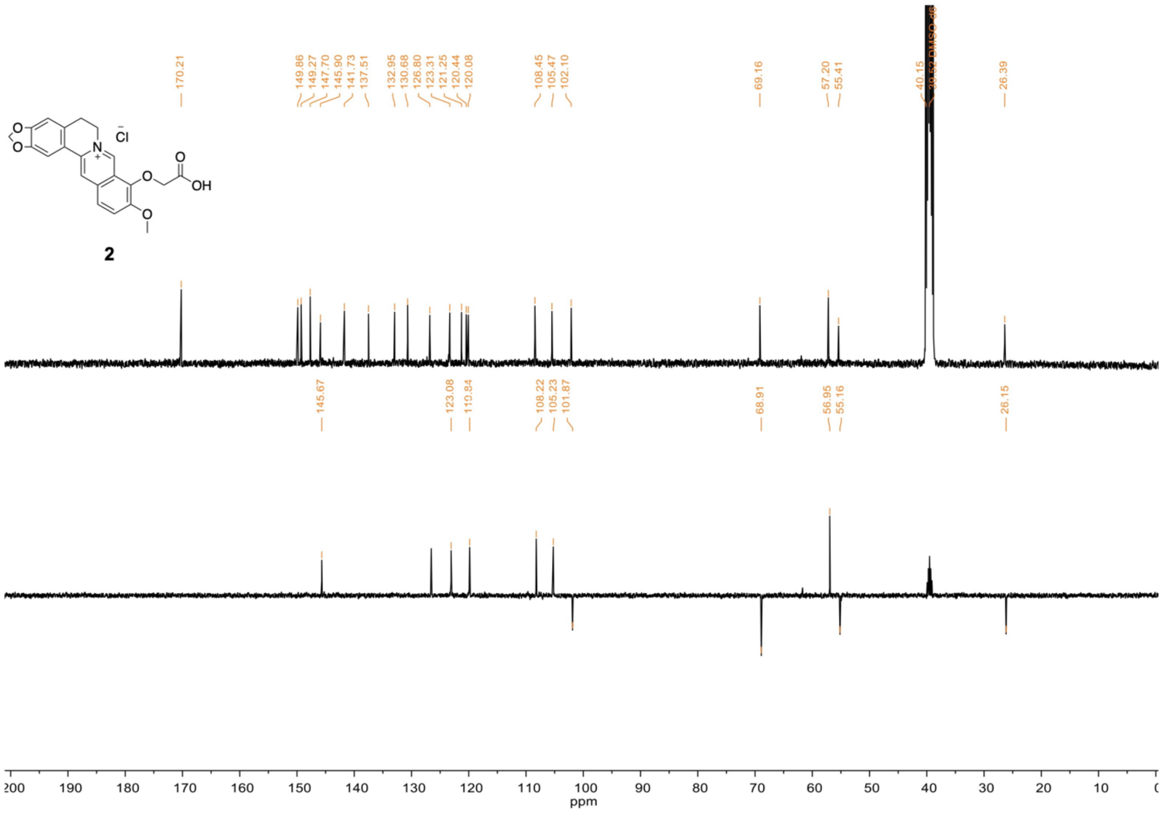

Figure S7 ^{13}C NMR (101 MHz, $(CD_3)_2SO$, 298K) of **compound 2**.

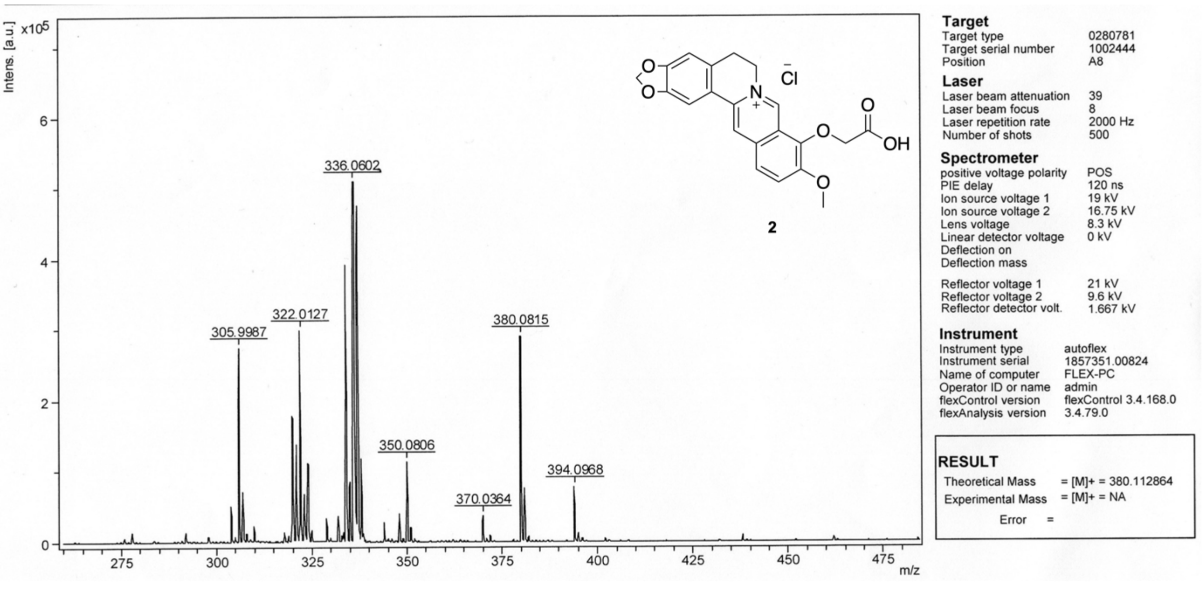

Figure S8 HRMS (MADLI–TOF) analysis of **compound 2**: calculated for $C_{21}H_{18}NO_6^+$ $[M]^+$ *m/z* 380.1129, found 380.0815.

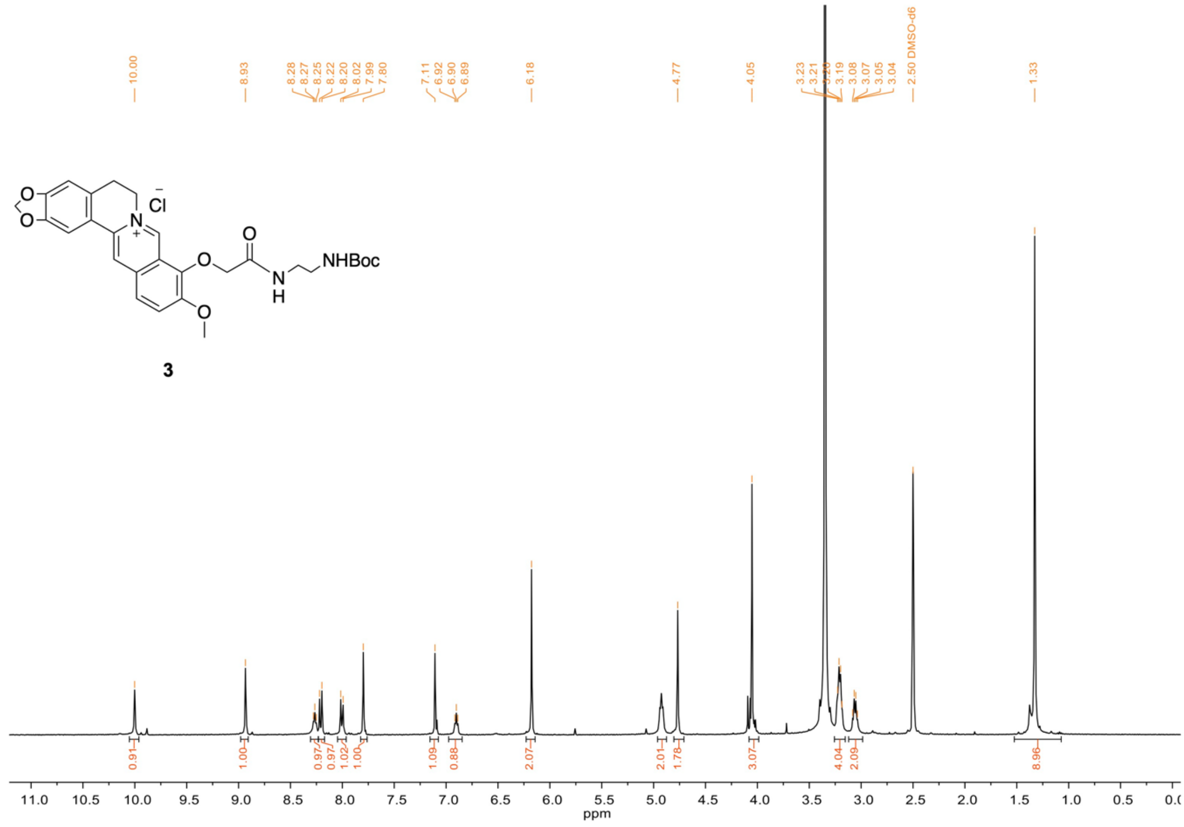

Figure S9 ^{1}H NMR (400 MHz, $(CD_3)_2SO$, 298K) of **compound 3**.

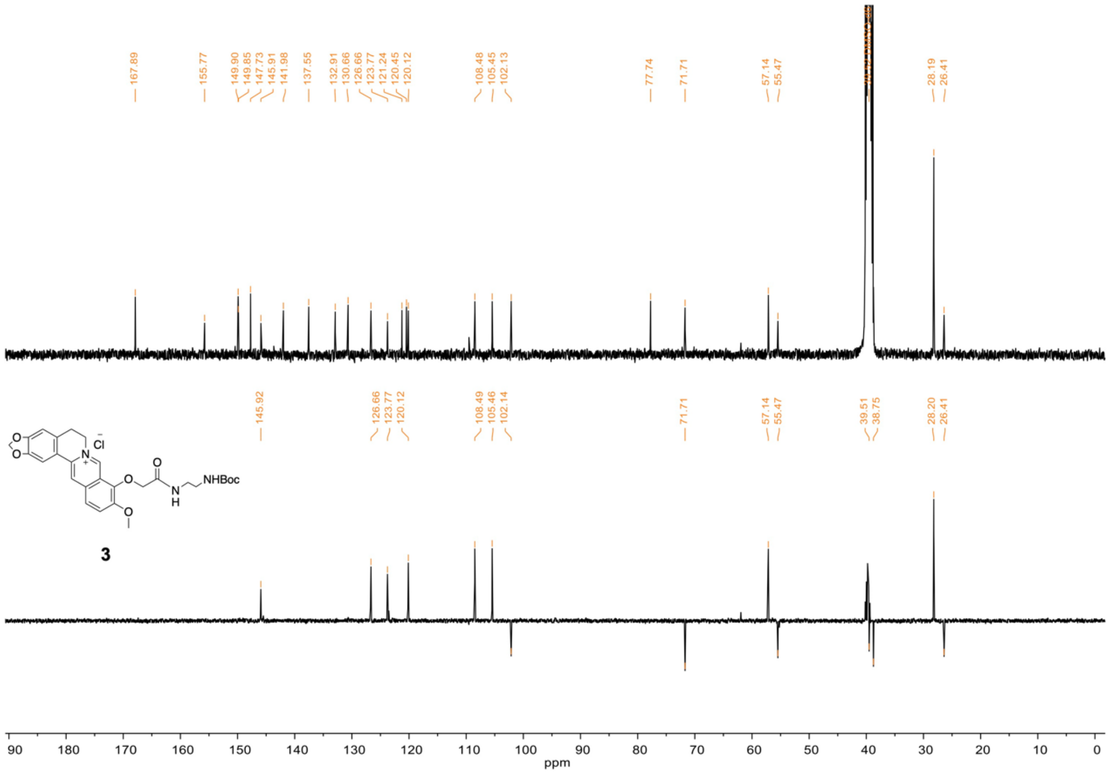

Figure S10 ^{13}C NMR (101 MHz, $(CD_3)_2SO$, 298K) of **compound 3**.

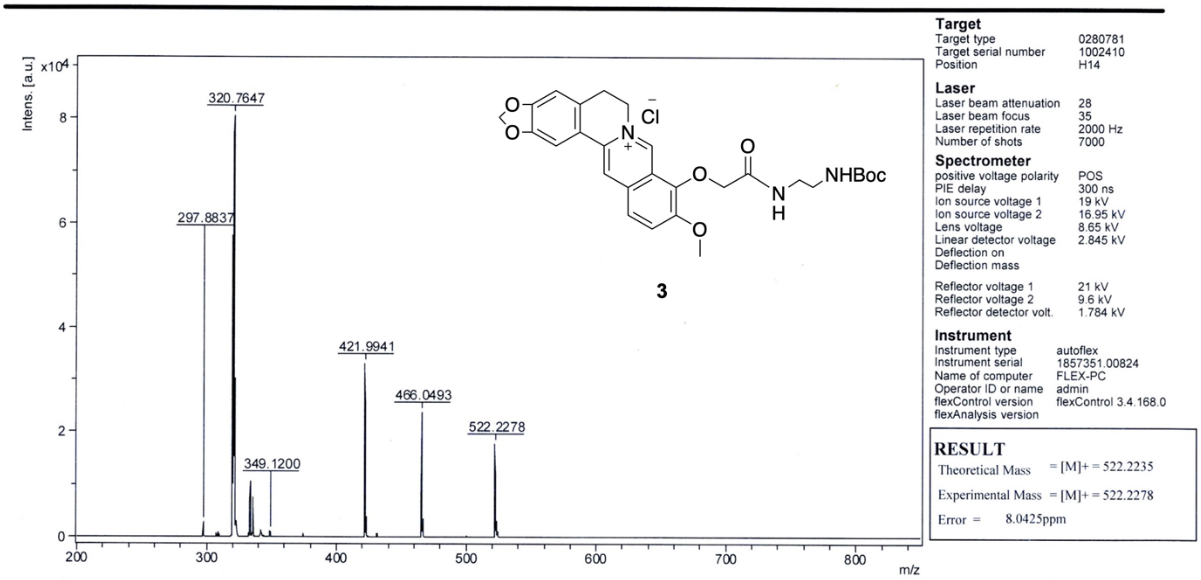

Figure S11 HRMS (MADLI–TOF) analysis of **compound 3**: calculated for $C_{28}H_{32}N_{3}O_{7}^{+}$ $[M]^{+}$ *m/z* 522.2235, found 522.2278.

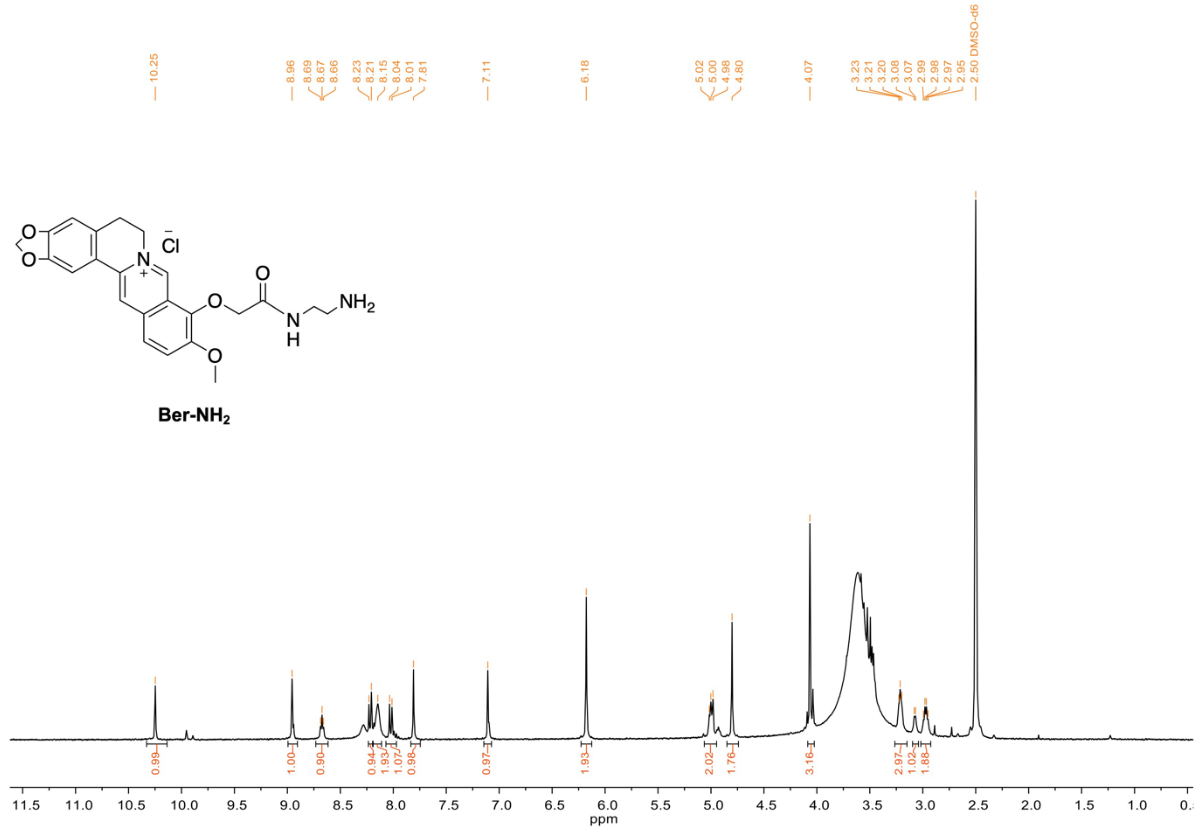

Figure S12 ^{1}H NMR (400 MHz, $(CD_3)_2SO$, 298K) of **Ber-NH₂**.

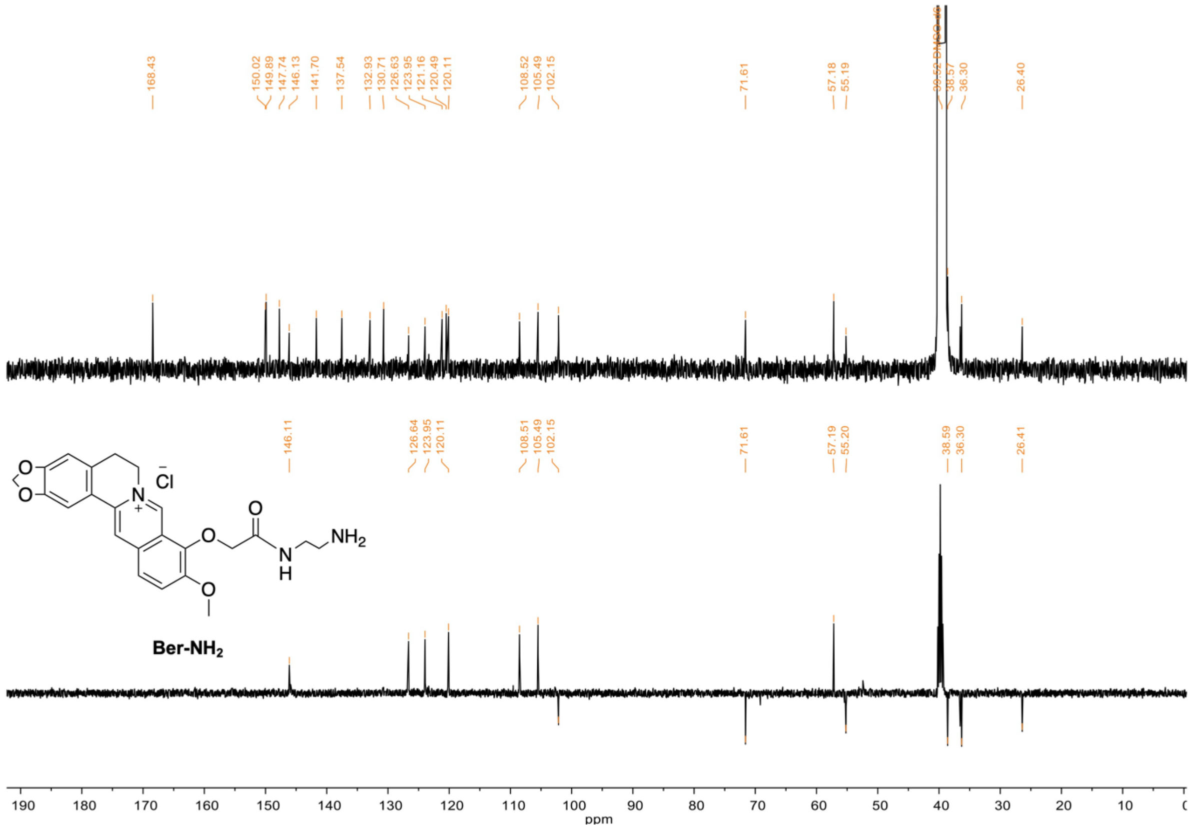

Figure S13 ^{13}C NMR (101 MHz, $(CD_3)_2SO$, 298K) of **Ber-NH₂**.

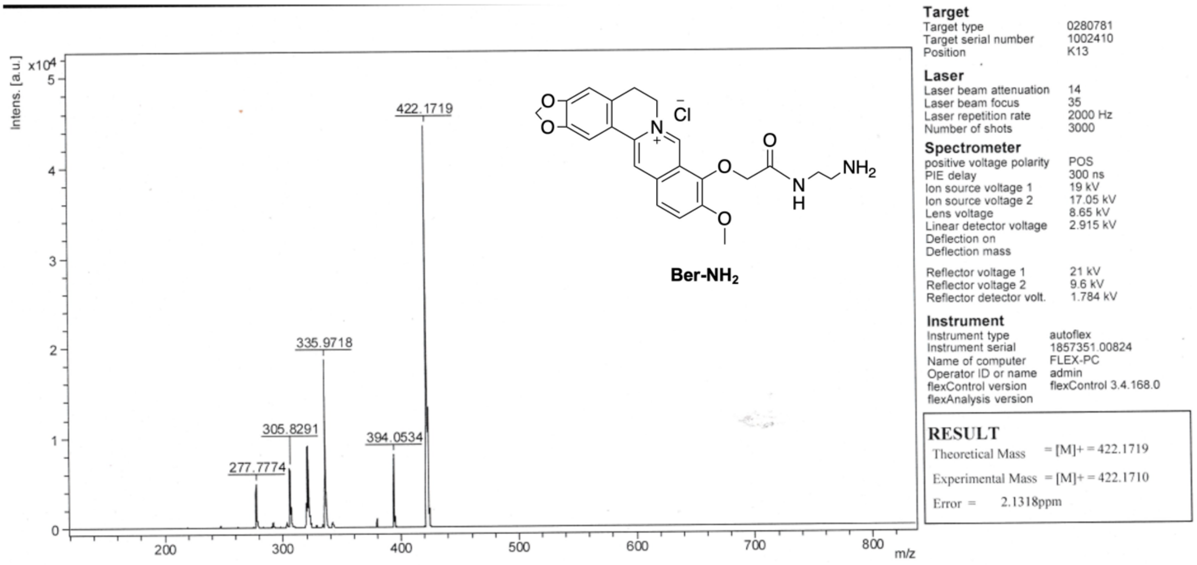

Figure S14 HRMS (MADLI–TOF) analysis of **Ber-NH₂**: calculated for $C_{23}H_{24}N_3O_5^+$ $[M]^+$ *m/z* 422.1719, found 422.1710.

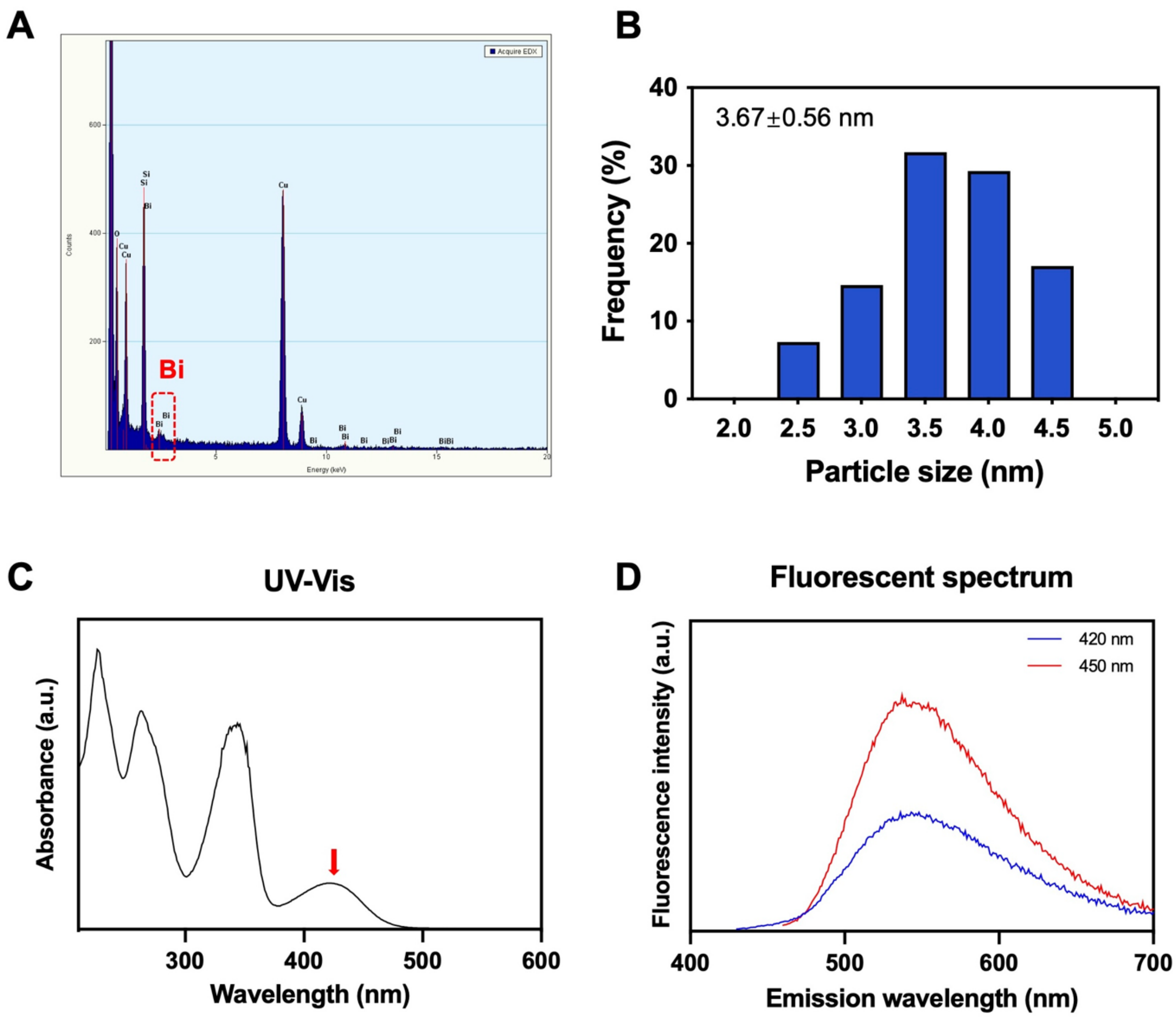

Figure S15 (A) Energy dispersive X-ray spectroscopy (EDX) analysis showing the general elemental composition of BiCD. (B) Estimated size distribution of BiCD (n = 41). (C) UV-Vis and (D) fluorescent spectra of Ber-NH_2, and red arrow highlight its absorption at 430 nm.

Biological assays

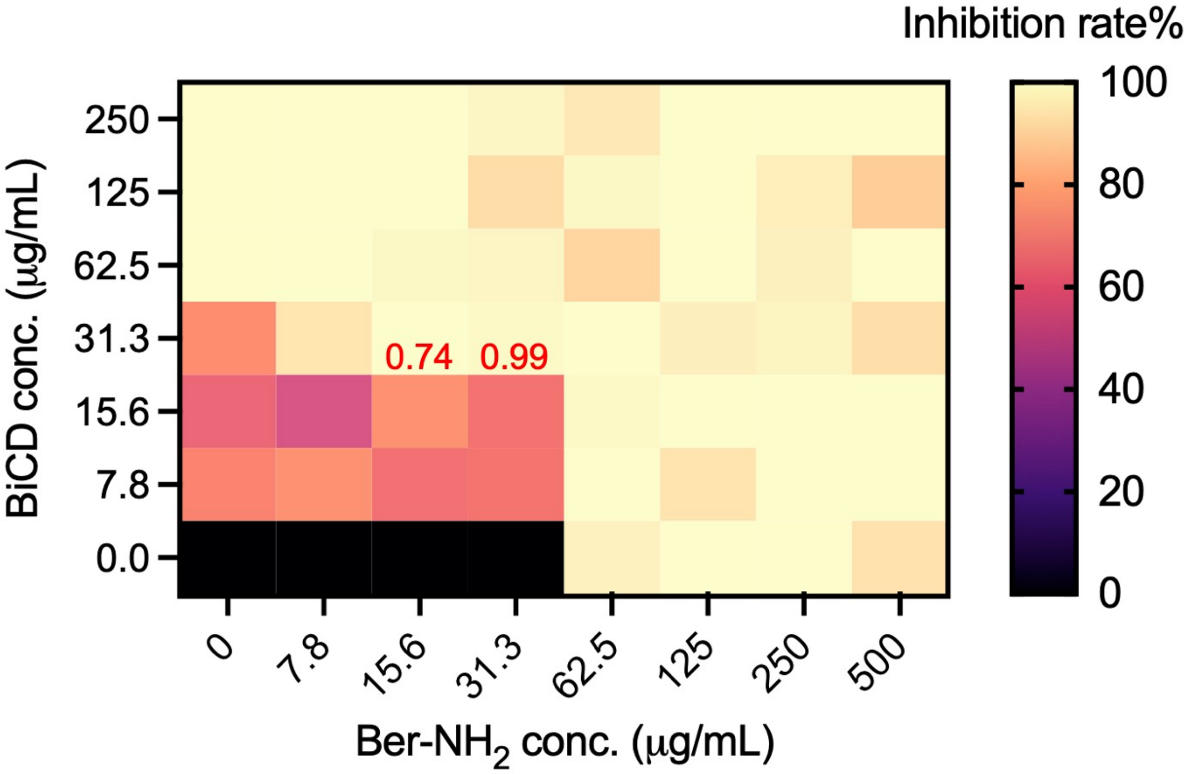

Figure S16 Checkerboard analysis of the combined effects of BiCD and Ber-NH_2 on *P. gingivalis* and the FIC indices.

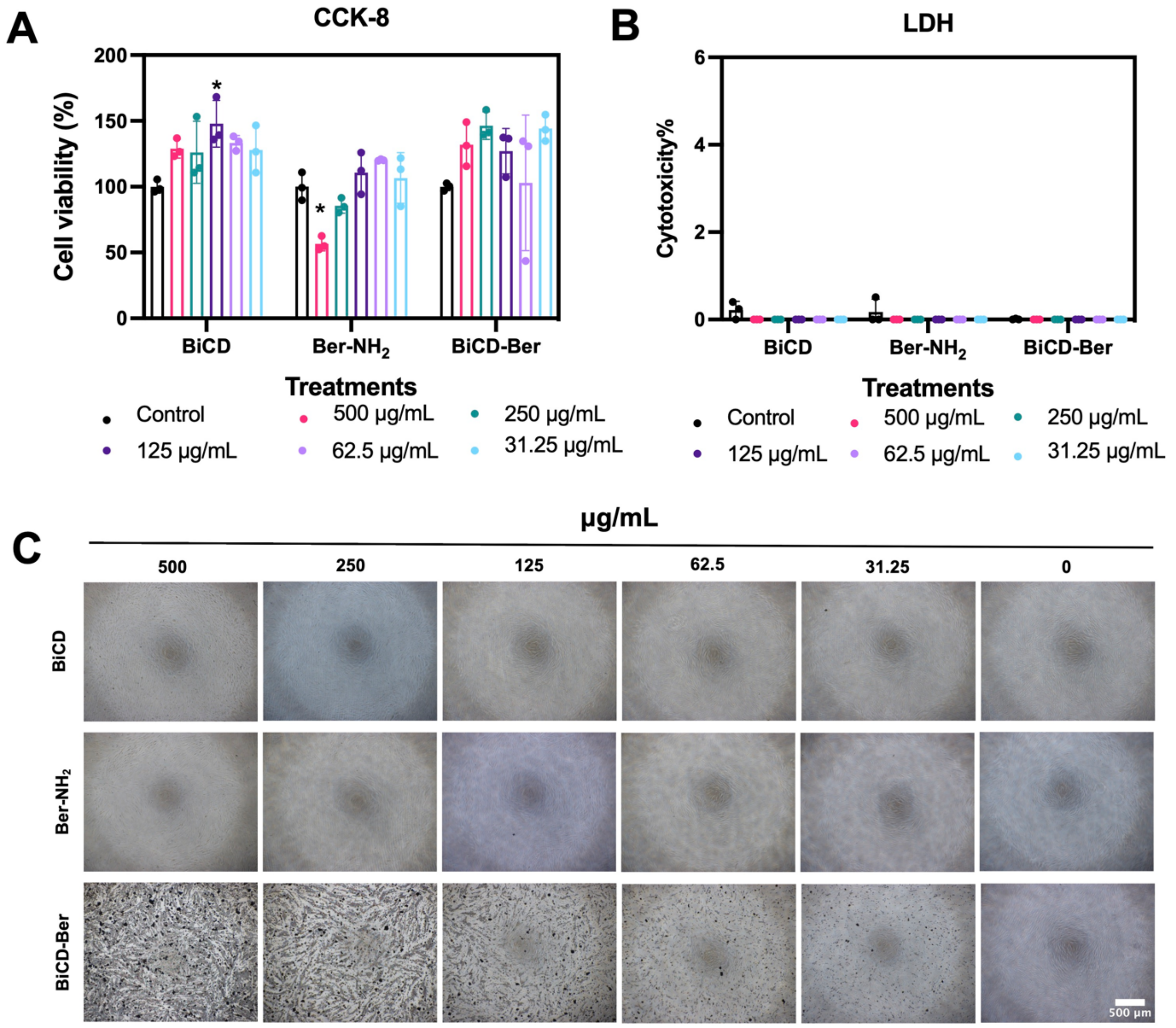

Figure S17 Cytotoxicity evaluation of BiCD, Ber-NH_2 and BiCD-Ber on pHGF using CCK-8 (A) and LDH (B) assays, and the optical images of cells after the treatments at different concentrations (scale bar: 500 μm) (C).

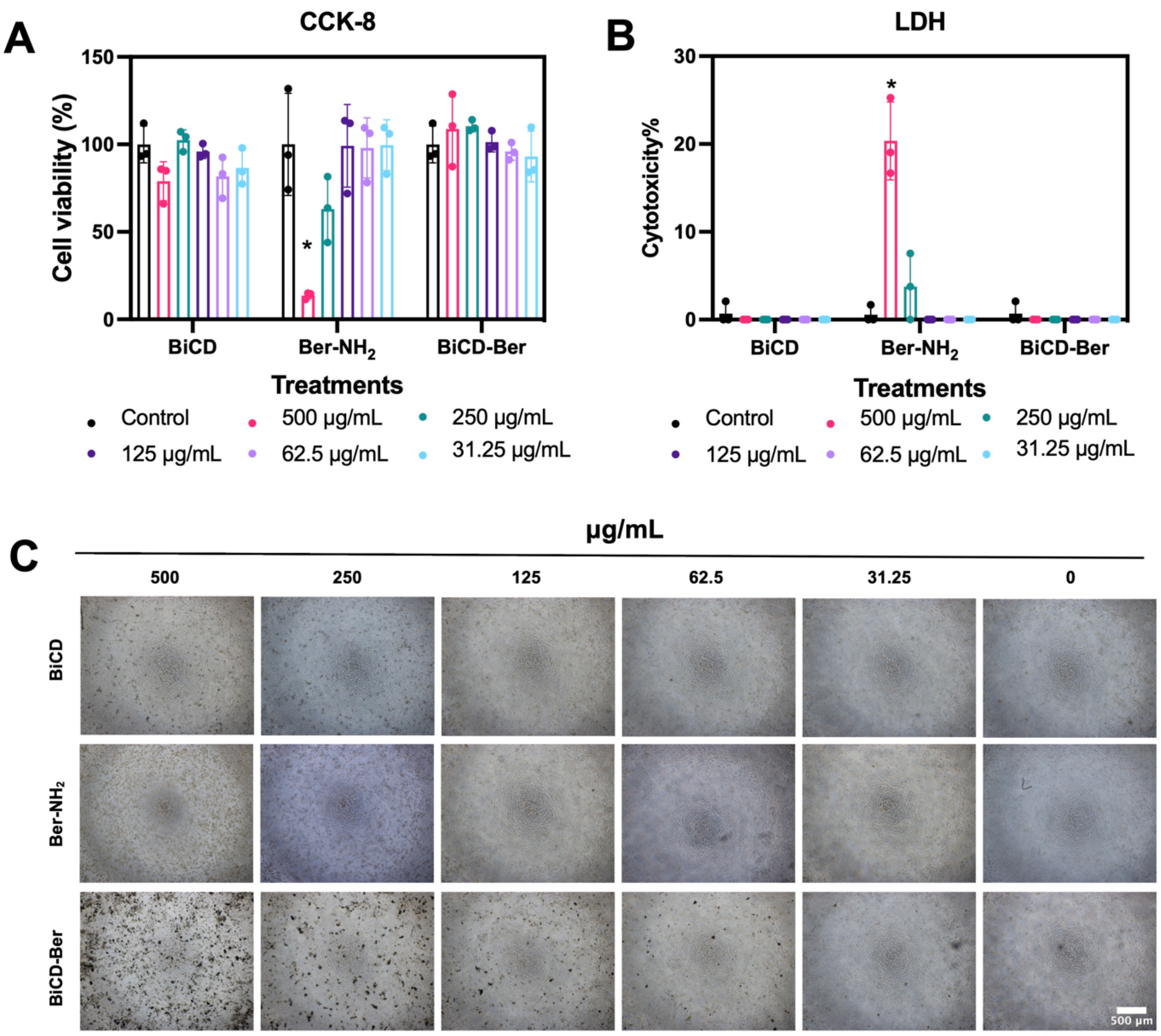

Figure S18 Cytotoxicity evaluation of BiCD, Ber-NH$_2$ and BiCD-Ber on HGECs using CCK-8 (A) and LDH (B) assays, and the optical images of cells after the treatments at different concentrations (scale bar: 500 μm) (C).

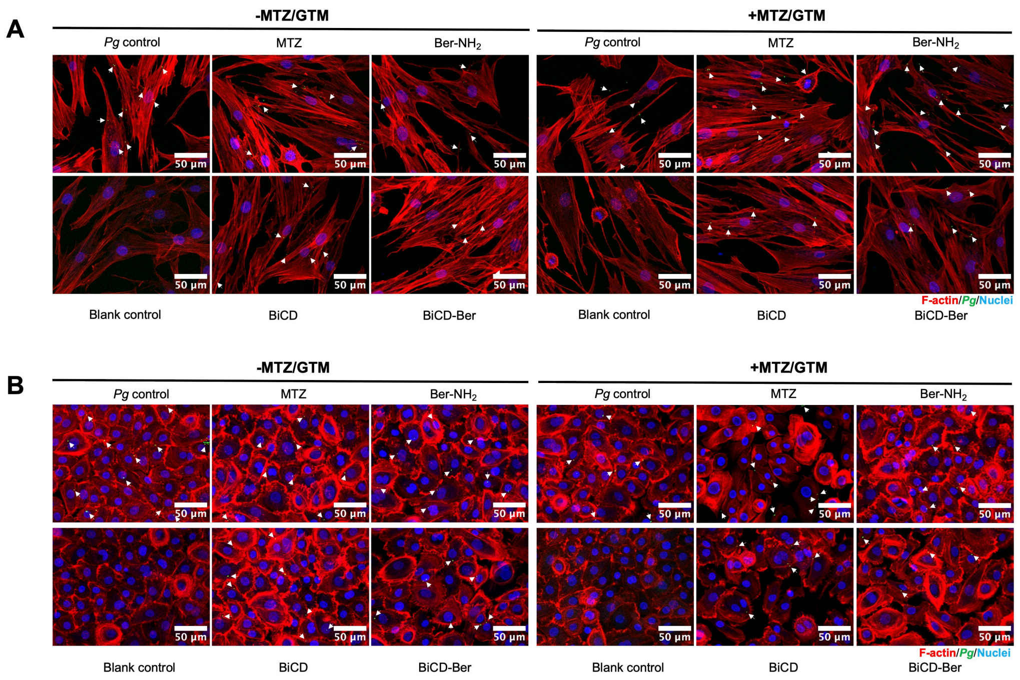

Figure S19 Immunofluorescent staining of *Pg*-infected pHGF (A) and HGECs (B) with and without high-concentration washing of MTZ and GTM. Following the infection, the cells were treated with MTZ (20 μg/mL), Ber-NH2, BiCD, and BiCD-Ber (100 μg/mL) for a specific duration. *Pg* was stained green, F-actin in the cells was labeled red, and the cell nuclei were highlighted in a pseudo-color of blue (scale bar: 50 μm, white arrows indicate the presence of *Pg*).

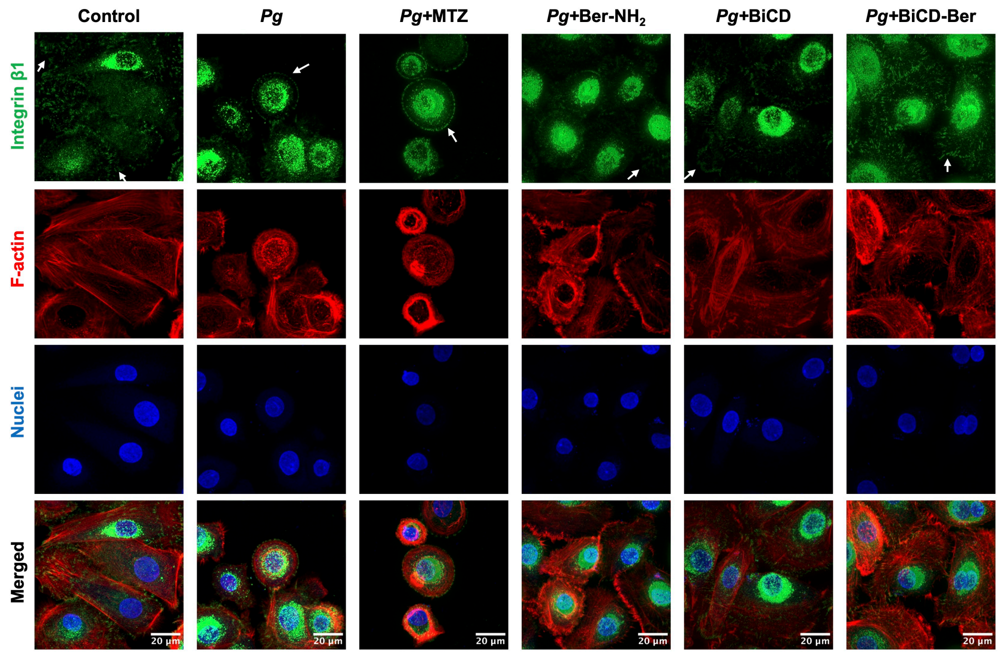

Figure S20 Immunofluorescent staining of integrin β1 and F-actin in HGECs infected by *Pg* with or without treatments of drugs (MTZ or Ber-NH_2) or nanomedicines (BiCD or BiCD-Ber)

for 24 h (scale bar: 20 μm). The white arrows indicate membrane-bound integrin β1 located at cell edges.

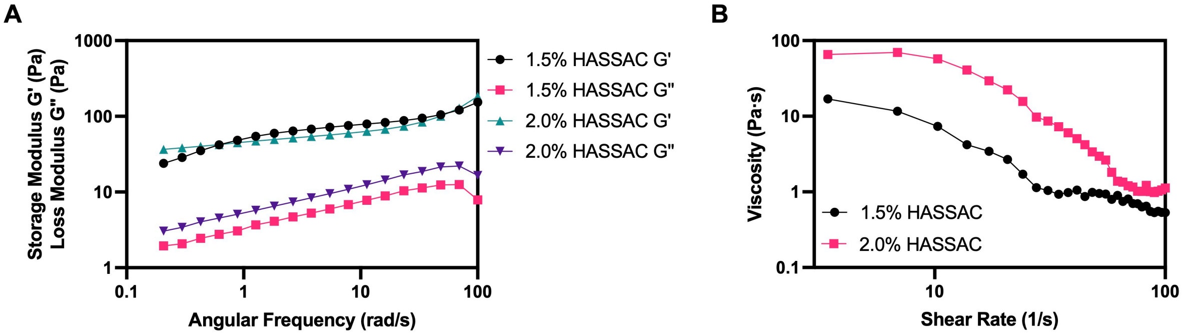

Figure S21 Storage modulus (G') and loss modulus (G'') of hydrogels constructed at 1.5% and 2.0% HASSAC (A). The viscosity-shear rate of hydrogels constructed from different HASSAC concentration (1.5% and 2.0%).

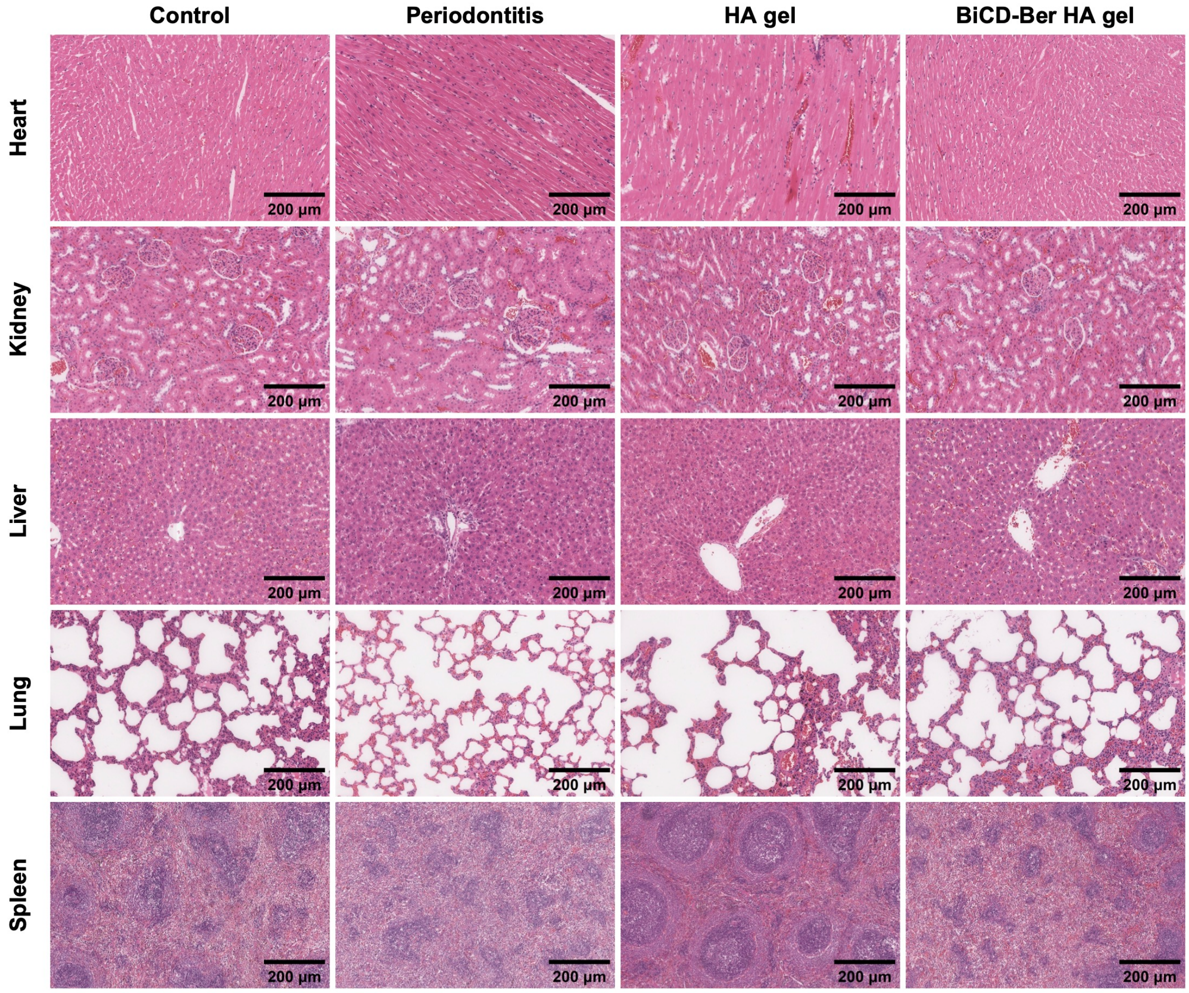

Figure S22 Hematoxylin and eosin staining of vital organs (heart, kidney, liver, lung and spleen) in different groups (scale bar: 200 μm).

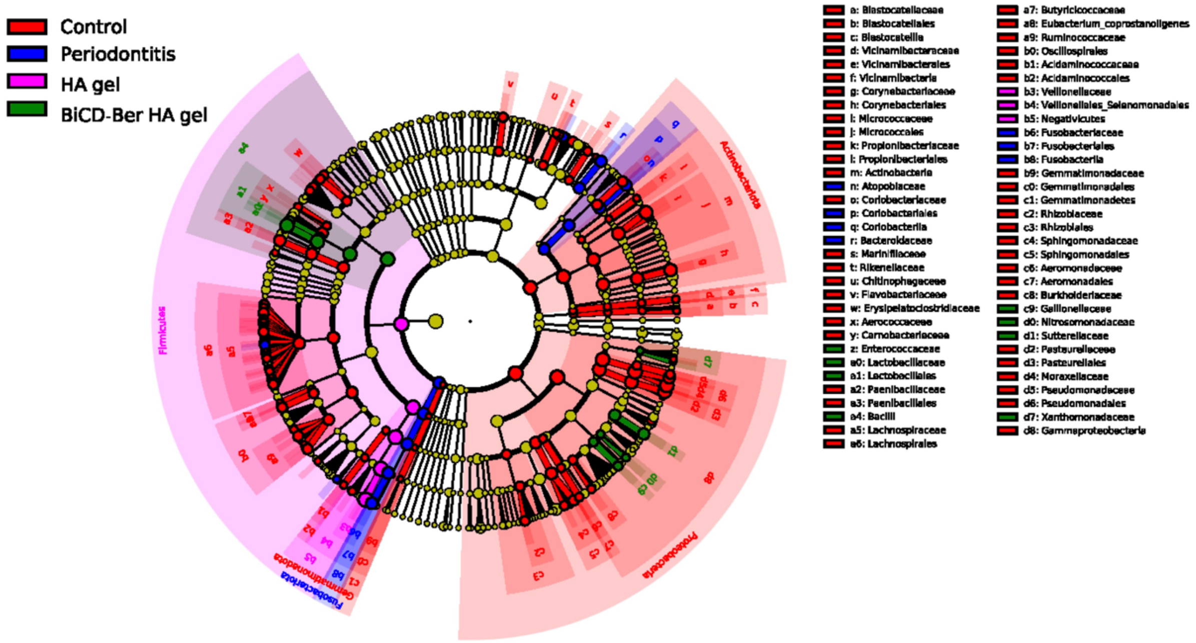

Figure S23 LEfSe analysis of periodontal microbiota. In the LEfSe tree, different colours indicated different groups, while the important microbe biomarkers affiliated to each group at upper right side were labelled with the corresponding colour of the groups present at upper left side. The yellow dots represented the biomarker which did not show any importance in different groups.

Table S1 Primers used in this study.

**Primer**	**Sequence (5'-3')**	**Description/Use**
pET28a-RgpB$_{230-736}$-Forward	ATATTA<u>GAATTC</u>TATACGCCTGTTGAAGAAAA	Cloning RgpB$_{230-736}$ into pET28a
pET28a-RgpB$_{230-736}$-Reverse	ATATAT<u>CTCGAG</u>TTACTTCACTATAACCTTTT	Cloning RgpB$_{230-736}$ into pET28a
pET28a-Kgp$_{229-595}$-Forward	ATATAT<u>GGATCC</u>GATGTTTATACAGATCATGGC	Cloning Kgp$_{229-595}$ into pET28a

pET28a-Kgp$_{229-595}$-Reverse	ATATATCTCGAGTTAAGGCATAACCGAACCAT	Cloning Kgp$_{229-595}$ into pET28a

Table S2 Relative abundances of phylum, genus and species levels in different groups.

	Mean (BiCD-Ber HA gel)	SD (BiCD-Ber HA gel)	Mean (Control)	SD (Control)	Mean (HA gel)	SD (HA gel)	Mean (Periodontitis)	SD (Periodontitis)	*p*-value	FDR
**Phylum**										
Chloroflexi	0.057696	0.026096	0.073192	0.09101	0.019433	0.02729	0.013221	0.028334	0.007971	0.035072
Myxococcota	0.0	0.0	0.003506	0.006949	0.0	0.0	3.8E-4	0.001202	0.023031	0.074357
Planctomycetota	0.0	0.0	0.001777	0.003106	0.0	0.0	0.0	0.0	0.023659	0.074357
Deinococcota	0.210142	0.205916	0.197053	0.417807	0.113945	0.145308	0.080647	0.194706	0.065778	0.18089
Cyanobacteria	0.017989	0.014679	0.034991	0.038749	0.012843	0.015281	0.007615	0.013604	0.082528	0.201735
Methylomirabilota	0.0	0.0	0.001597	0.003546	0.0	0.0	0.0	0.0	0.104361	0.224674
Unclassified	0.0	0.0	0.013536	0.021715	0.001301	0.004113	0.016864	0.048403	0.112337	0.224674
Verrucomicrobiota	0.150455	0.189501	0.035315	0.023203	0.913448	1.729687	0.668192	1.176939	0.127367	0.233506
Acidobacteriota	0.009162	0.009494	0.05704	0.088155	0.003899	0.005387	0.006277	0.016234	0.217897	0.368749
Nitrospirota	0.0	0.0	0.002537	0.005373	0.0	0.0	4.18E-4	0.001323	0.260901	0.398981
Deferribacterota	0.005371	0.012481	0.018702	0.024945	0.006216	0.009972	0.005274	0.013095	0.286221	0.398981
Patescibacteria	0.0017	0.003635	0.070374	0.174892	0.054398	0.121799	0.022481	0.036937	0.290168	0.398981
Bdellovibrionota	0.004907	0.007882	0.001555	0.003431	0.003289	0.010402	5.74E-4	0.001815	0.320107	0.414256
Bacteroidota	1.280518	1.10489	5.363505	10.487223	3.509593	4.543951	4.785825	5.732283	0.520605	0.636295
Desulfobacterota	0.011614	0.013062	0.054224	0.102693	0.039364	0.063959	0.023587	0.021031	0.567796	0.642332
Campilobacterota	0.018569	0.012335	0.087653	0.182322	0.024348	0.024097	0.016281	0.021852	0.583938	0.642332
Gemmatimonadota	0.00131	0.003054	0.002912	0.005307	0.002068	0.004804	5.85E-4	0.00185	0.719133	0.753377
Spirochaetota	0.001909	0.004381	5.17E-4	0.001635	0.001021	0.003228	0.00234	0.005661	0.81916	0.81916
Proteobacteria	21.025605	11.364168	59.573944	15.438715	16.158659	6.175702	26.958326	10.90539	3.7E-5	3.45E-4
Fusobacteriota	1.480022	1.034192	0.059965	0.04532	1.029927	0.832013	2.57927	3.527351	4.7E-5	3.45E-4
Actinobacteriota	6.339108	4.279118	20.581902	8.394396	5.370595	4.249692	8.292753	6.67791	5.32E-4	0.002926
Firmicutes	69.383923	12.464186	13.764202	8.726797	72.735653	11.445791	56.519089	14.83758	9.0E-6	1.98E-4
**Genus**										
Coprococcus	9.44E-4	0.002986	0.16127	0.275905	0.0	0.0	0.0	0.0	0.0	0.0
Eubacterium_ruminantium	0.0	0.0	0.295129	0.585488	0.0016	0.003388	9.56E-4	0.003024	6.1E-5	0.001017
Enterobacter	0.016612	0.027724	0.115712	0.079688	0.01003	0.024902	0.071677	0.037598	6.8E-5	0.00105

Subdoligranulum	0.012654	0.017653	0.384408	0.529872	0.017251	0.017045	0.00961	0.018048	7.0E-5	0.00105
Parasutterella	1.689031	1.55401	0.086297	0.109509	1.436768	0.633578	1.292487	0.876053	8.0E-5	0.001143
Rothia	1.208799	0.899389	11.220014	6.814837	0.675831	1.122837	1.072013	0.891571	9.0E-5	0.001227
Lachnospira	0.006393	0.011537	0.046002	0.057105	0.0	0.0	0.001889	0.004326	1.06E-4	0.001383
Pygmaiobacter	0.0	0.0	0.007422	0.010153	0.0	0.0	0.0	0.0	1.36E-4	0.0017
Sphingomonas	0.047842	0.033216	0.416113	0.399641	0.039815	0.036313	0.043447	0.02717	1.81E-4	0.002172
Roseburia	0.001957	0.006189	0.085801	0.129698	0.004852	0.010921	0.0	0.0	2.81E-4	0.003242
Acinetobacter	6.984636	9.082853	33.249203	18.385667	5.082749	3.782914	13.992748	10.827388	3.37E-4	0.003744
Faecalibacterium	0.029731	0.038011	0.397253	0.478659	0.027164	0.031945	0.023513	0.032436	3.74E-4	0.004007
Paenibacillus	0.056569	0.122117	2.003359	5.051419	0.022786	0.035493	0.052246	0.057821	4.47E-4	0.004624
Eubacterium_coprostanoligenes	0.00628	0.010288	0.261544	0.415407	0.007611	0.01138	0.029859	0.047429	5.01E-4	0.00501
Dorea	9.02E-4	0.002853	0.140442	0.189408	0.01126	0.019785	0.006214	0.010952	6.11E-4	0.005913
Klebsiella	0.028846	0.039999	0.273129	0.219455	0.088453	0.061106	0.04456	0.036602	6.82E-4	0.006394
Burkholderia_Caballeronia_Paraburkholderia	0.007867	0.008815	0.040449	0.021364	0.023986	0.014816	0.02663	0.011624	8.01E-4	0.006943
Erysipelotrichaceae_UCG_003	0.00468	0.007972	0.084254	0.144386	0.007044	0.014259	0.001236	0.002611	8.08E-4	0.006943
Lachnospiraceae_ND3007	5.87E-4	0.001857	0.036211	0.037499	0.001819	0.003863	0.002432	0.00392	8.1E-4	0.006943
Ruminococcus_gauvreauii	0.0	0.0	0.036803	0.067558	0.0	0.0	0.0	0.0	8.46E-4	0.00705
Aquabacterium	0.01916	0.052266	0.269668	0.292849	0.015446	0.02306	0.084584	0.093935	9.99E-4	0.0081
Carnobacterium	0.031935	0.08192	0.636002	0.945799	0.01007	0.026579	0.118909	0.110828	0.001247	0.009845
Pseudomonas	0.033418	0.034978	0.065415	0.054218	0.003771	0.006234	0.013305	0.012426	0.001311	0.010085
Prevotellaceae_UCG_001	0.001556	0.003464	0.113561	0.250569	0.210375	0.465559	0.26114	0.457728	0.001493	0.011197
Blastomonas	0.007175	0.02269	0.087738	0.079311	0.004255	0.008973	0.01958	0.02664	0.001598	0.011693
Cutibacterium	5.66E-4	0.001791	0.007307	0.007691	0.0	0.0	6.26E-4	0.001979	0.001758	0.012557
Psychrobacter	0.151443	0.370151	1.957112	1.49262	0.091317	0.156409	0.686507	1.118156	0.001801	0.012565
Alistipes	0.004366	0.006868	0.092879	0.184168	0.001617	0.003541	0.004217	0.005619	0.003303	0.02252
Eubacterium_eligens	0.0	0.0	0.030138	0.043573	0.0	0.0	0.001148	0.003629	0.003388	0.022587
Allorhizobium_Neorhizobium_Pararhizobium_Rhizobium	0.050012	0.031619	0.182815	0.170763	0.032795	0.037547	0.081309	0.060346	0.003802	0.024796
Allobaculum	0.003063	0.006964	0.025811	0.021793	6.96E-4	0.0022	0.004773	0.010858	0.004497	0.027317
Butyricicoccus	0.0	0.0	0.009221	0.012847	0.0	0.0	0.0	0.0	0.004735	0.027317
Catenibacillus	0.0	0.0	0.02201	0.047526	0.0	0.0	0.0	0.0	0.004735	0.027317

Flavobacterium	0.008775	0.020439	0.103	0.079359	0.011061	0.016272	0.028319	0.031736	0.004453	0.027317
Rikenella	0.0	0.0	0.021246	0.039093	0.0	0.0	0.0	0.0	0.004735	0.027317
Vicinamibacteraceae	0.0	0.0	0.013956	0.021865	0.0	0.0	0.001521	0.00481	0.004547	0.027317
Duganella	0.099251	0.252697	0.37503	0.329998	0.021117	0.036249	0.165532	0.143237	0.004911	0.027528
Ruminococcus	0.021464	0.030265	0.180101	0.266353	0.071491	0.14685	0.239558	0.390138	0.004955	0.027528
Lachnospiraceae_UCG_006	0.00137	0.004332	0.017187	0.033486	0.0	0.0	0.0	0.0	0.005237	0.028565
Bacteroides	0.266759	0.169049	0.116696	0.09701	0.494727	0.470473	1.406186	2.363016	0.006183	0.032984
Dialister	0.11076	0.118025	0.036203	0.062227	8.08E-4	0.002554	0.0	0.0	0.006267	0.032984
Blautia	0.072334	0.122046	0.434486	0.45689	0.092758	0.167815	0.067652	0.072914	0.007184	0.037159
Clostridia_UCG_014	0.006606	0.008867	0.450349	0.85164	0.067441	0.135137	0.109184	0.146286	0.008386	0.042641
Aerococcus	0.00464	0.008379	0.024382	0.021203	0.001235	0.002862	0.012771	0.026218	0.009337	0.046685
Holdemanella	3.61E-4	0.001141	0.053954	0.101667	5.48E-4	0.001734	0.0	0.0	0.010498	0.05163
Phascolarctobacterium	0.003924	0.010558	0.012504	0.013991	0.002833	0.008957	0.002281	0.007215	0.011211	0.054247
Limnobacter	0.011964	0.031564	0.051847	0.074389	0.002565	0.008111	0.04265	0.051835	0.013058	0.062181
Corynebacterium	3.261753	3.933767	7.143934	4.150962	2.500633	2.835833	2.611236	3.184141	0.013579	0.063652
Oscillospiraceae	0.0	0.0	0.010186	0.011321	0.0	0.0	0.006144	0.011801	0.013978	0.064514
Planococcus	0.007594	0.020228	0.086026	0.137916	0.001988	0.004744	0.030534	0.035583	0.015408	0.070036
Escherichia_Shigella	2.804834	3.322833	1.147898	0.592893	3.009162	1.325137	2.22251	1.239024	0.016152	0.072322
Eubacterium_brachy	0.0	0.0	0.00584	0.009178	0.0	0.0	4.34E-4	0.001373	0.017915	0.076779
Hungatella	0.0	0.0	0.0	0.0	0.001821	0.005759	0.196776	0.515345	0.017915	0.076779
Sphingobium	0.042073	0.093059	0.278761	0.267377	0.026311	0.03702	0.096685	0.101974	0.017877	0.076779
Actinomyces	0.0	0.0	0.009993	0.013504	0.0	0.0	0.002086	0.006596	0.020357	0.082531
Anaerostignum	0.0	0.0	0.019871	0.042622	0.0	0.0	0.0	0.0	0.023659	0.082531
Anaerotruncus	0.0	0.0	0.014246	0.028937	0.0	0.0	0.0	0.0	0.023659	0.082531
Arenimonas	0.002572	0.00417	0.0	0.0	0.0	0.0	0.0	0.0	0.023659	0.082531
Aridibacter	0.0	0.0	0.006131	0.010595	0.0	0.0	0.0	0.0	0.023659	0.082531
Barnesiella	0.0	0.0	0.003857	0.006364	0.0	0.0	0.0	0.0	0.023659	0.082531
Eubacterium_siraeum	0.0	0.0	0.023083	0.043268	0.0	0.0	0.007627	0.0118	0.022092	0.082531
Jeotgalicoccus	0.0	0.0	0.005533	0.010621	0.0	0.0	0.0	0.0	0.023659	0.082531
Lachnospiraceae_FCS020	0.0	0.0	0.016958	0.032477	0.0	0.0	0.0	0.0	0.023659	0.082531

Lachnospiraceae_UCG_010	0.0	0.0	0.018548	0.038395	0.0	0.0	0.0	0.0	0.023659	0.082531
Lentimicrobium	0.002818	0.004156	6.9E-4	0.00218	0.0	0.0	0.0	0.0	0.023031	0.082531
Lysobacter	0.0	0.0	0.003021	0.005135	0.0	0.0	0.0	0.0	0.023659	0.082531
Megasphaera	0.0	0.0	0.011124	0.023	0.0	0.0	0.0	0.0	0.023659	0.082531
Microbacterium	0.136334	0.17196	0.056555	0.11928	0.077088	0.112967	0.014545	0.036583	0.021094	0.082531
Muribaculum	0.0	0.0	0.041403	0.082073	0.002627	0.005717	0.001255	0.003969	0.02026	0.082531
Senegalimassilia	0.001133	0.003583	0.005332	0.007297	0.0	0.0	0.0	0.0	0.023031	0.082531
Kocuria	0.003151	0.006805	0.032015	0.030829	0.001044	0.0033	0.009852	0.018606	0.025854	0.089152
Rodentibacter	6.269301	3.973393	14.818698	11.844099	3.731934	2.198107	5.934684	3.406657	0.026387	0.089956
Odoribacter	0.004134	0.005494	0.059972	0.08978	0.002343	0.005811	0.004143	0.004461	0.030652	0.103321
Kapabacteriales	0.00528	0.007198	0.0	0.0	0.003456	0.005618	0.0	0.0	0.032993	0.109977
Megamonas	7.45E-4	0.002357	0.026128	0.040314	0.005431	0.011603	0.001808	0.003867	0.037652	0.122778
Negativibacillus	0.0	0.0	0.014467	0.02537	0.001817	0.005745	0.003225	0.007721	0.037475	0.122778
Alloprevotella	0.018102	0.014991	0.468959	1.067062	0.043536	0.036902	0.085901	0.12598	0.045034	0.145271
Anaeroplasma	0.017538	0.010723	0.021357	0.013367	0.010175	0.009452	0.009845	0.007493	0.048968	0.156281
Romboutsia	0.01921	0.019967	0.0818	0.090841	0.278645	0.512232	0.225273	0.297505	0.056075	0.177079
Unclassified	0.18439	0.096314	1.12481	1.748567	0.382169	0.54454	0.268645	0.334759	0.0567	0.177187
Chryseobacterium	0.131766	0.210419	0.050715	0.118321	0.087932	0.245208	0.004943	0.015632	0.058646	0.181379
Deinococcus	0.210142	0.205916	0.196637	0.418022	0.113945	0.145308	0.080647	0.194706	0.059873	0.183285
Micrococcus	0.073529	0.04209	0.157535	0.27182	0.066196	0.077113	0.044305	0.10251	0.061663	0.185019
Weissella	0.001305	0.003075	0.007923	0.020329	0.0	0.0	0.005734	0.006792	0.061673	0.185019
Brevundimonas	0.365249	0.380343	0.858737	0.671715	0.171739	0.157454	0.423657	0.375985	0.06532	0.19402
Gemella	0.002156	0.004547	0.006948	0.006604	0.003624	0.005873	0.001043	0.003298	0.073365	0.215779
Staphylococcus	0.037309	0.051589	0.142774	0.19987	0.047628	0.02992	0.02339	0.028449	0.075139	0.218851
Eubacterium_ventriosum	0.0	0.0	0.011318	0.023061	0.0	0.0	3.83E-4	0.00121	0.078085	0.225245
Porphyromonas	0.227167	0.362729	0.037454	0.077315	0.155638	0.401974	0.743062	1.868247	0.079304	0.226583
Desemzia	0.001511	0.004777	0.016967	0.025734	0.0	0.0	0.003929	0.00894	0.082013	0.232112
Acetatifactor	0.0	0.0	0.009891	0.029441	0.0	0.0	0.0	0.0	0.104361	0.237184
Actinoplanes	0.0	0.0	0.002256	0.005158	0.0	0.0	0.0	0.0	0.104361	0.237184
Bacteriovorax	0.004528	0.008032	0.0	0.0	0.002741	0.008668	0.0	0.0	0.103817	0.237184

Blastocatella	0.0	0.0	0.001873	0.003957	0.0	0.0	0.0	0.0	0.104361	0.237184
Blastococcus	0.0	0.0	0.00238	0.005056	0.0	0.0	0.0	0.0	0.104361	0.237184
Brevibacterium	0.0	0.0	0.002087	0.004894	0.0	0.0	0.0	0.0	0.104361	0.237184
Candidatus_Saccharimonas	0.0	0.0	0.069525	0.175245	0.054398	0.121799	0.021716	0.037224	0.086684	0.237184
Christensenellaceae_R_7	0.008977	0.01763	0.036972	0.033207	0.132075	0.268302	0.075483	0.107928	0.086981	0.237184
Chthonobacter	0.0	0.0	0.0	0.0	0.00278	0.006617	0.0	0.0	0.104361	0.237184
Chujaibacter	0.0	0.0	0.0	0.0	0.001996	0.004228	0.0	0.0	0.104361	0.237184
Devosia	8.69E-4	0.002747	0.00339	0.00585	0.0	0.0	0.0	0.0	0.086301	0.237184
Eggerthella	0.0	0.0	0.004377	0.009349	0.0	0.0	0.0	0.0	0.104361	0.237184
Eubacterium_nodatum	0.0	0.0	0.009666	0.021524	0.0	0.0	0.001464	0.004631	0.086301	0.237184
Gemmatimonas	0.0	0.0	7.06E-4	0.001492	0.0	0.0	0.0	0.0	0.104361	0.237184
IMCC26256	0.0	0.0	0.001344	0.003211	0.0	0.0	0.0	0.0	0.104361	0.237184
Janibacter	0.0	0.0	0.004797	0.010138	0.0	0.0	0.0	0.0	0.104361	0.237184
Lautropia	0.0	0.0	0.00125	0.00264	0.0	0.0	0.0	0.0	0.104361	0.237184
Lysinibacillus	0.0	0.0	0.001084	0.002296	0.0	0.0	0.0	0.0	0.104361	0.237184
Microcoleus_Es_Yyy1400	0.0	0.0	0.001035	0.002354	0.0	0.0	0.0	0.0	0.104361	0.237184
Nocardioides	0.0	0.0	0.002049	0.004323	0.0	0.0	0.0	0.0	0.104361	0.237184
Novosphingobium	0.023392	0.035118	0.057131	0.062564	0.078447	0.19409	0.023951	0.055305	0.095286	0.237184
Oscillospira	0.0	0.0	0.0	0.0	0.0	0.0	0.007851	0.01707	0.104361	0.237184
Pelomonas	0.0	0.0	0.002622	0.005972	0.0	0.0	0.0	0.0	0.104361	0.237184
Pseudorhodoplanes	0.0	0.0	0.00157	0.003341	0.0	0.0	0.0	0.0	0.104361	0.237184
Rokubacteriales	0.0	0.0	0.001597	0.003546	0.0	0.0	0.0	0.0	0.104361	0.237184
Stenotrophobacter	0.0	0.0	0.003485	0.007348	0.0	0.0	0.0	0.0	0.104361	0.237184
Thermomonas	0.004915	0.008055	0.0	0.0	0.002986	0.006489	0.0	0.0	0.10797	0.243541
Akkermansia	0.150455	0.189501	0.034939	0.023362	0.913448	1.729687	0.668192	1.176939	0.111227	0.248034
Pedobacter	0.0	0.0	0.002531	0.004173	0.0	0.0	0.002102	0.005415	0.112442	0.248034
Terrisporobacter	0.006651	0.012907	0.003832	0.009001	0.0	0.0	0.0	0.0	0.112442	0.248034
Lachnospiraceae_UCG_001	0.0	0.0	0.011643	0.025615	0.0	0.0	0.00806	0.017622	0.116642	0.25542
Qipengyuania	0.557876	0.678611	1.651351	3.505555	0.661628	1.022271	0.409408	1.009788	0.118886	0.258448
Enhydrobacter	0.095944	0.130979	0.096346	0.17508	0.048906	0.102488	0.030516	0.057317	0.134547	0.290389

Clostridia_vadinBB60	0.00226	0.003949	0.010733	0.015304	0.001009	0.003192	0.001836	0.004184	0.135828	0.290806
Kytococcus	0.036976	0.026057	0.06094	0.149819	0.026552	0.040297	0.030144	0.075824	0.136679	0.290806
Granulicatella	0.439792	0.308024	0.593694	0.479492	0.269643	0.282652	0.471255	0.292869	0.144107	0.304451
Sphingobacterium	0.001313	0.003082	0.010878	0.015225	0.001832	0.005794	0.013343	0.023428	0.145914	0.306113
Paracoccus	1.263901	1.400841	3.168838	6.893005	1.347927	1.894313	1.152309	2.555222	0.15003	0.312563
Anaerostipes	0.004696	0.008866	0.030697	0.032384	0.00904	0.019675	0.04205	0.062445	0.165581	0.341933
Frisingicoccus	0.010659	0.014563	5.17E-4	0.001635	0.025284	0.052329	0.015898	0.026946	0.167336	0.341933
Lachnoclostridium	0.033858	0.04988	0.209312	0.376931	0.015602	0.036255	0.117762	0.227562	0.167547	0.341933
Haemophilus	0.001304	0.004125	0.013399	0.017031	0.005303	0.009996	0.005928	0.008117	0.18654	0.378122
Bosea	0.0	0.0	0.00579	0.00944	3.48E-4	0.0011	9.56E-4	0.003024	0.189189	0.378378
Clostridium_innocuum	5.66E-4	0.001791	0.0	0.0	0.004811	0.009441	0.00486	0.009991	0.188307	0.378378
Flavonifractor	0.005889	0.008972	0.002085	0.004506	0.0	0.0	0.004163	0.009446	0.193865	0.385162
Pantoea	0.209853	0.656084	0.01425	0.034847	0.0	0.0	0.014342	0.018832	0.196836	0.388492
Eubacterium_xylanophilum	0.001566	0.004951	0.063222	0.1192	0.012746	0.027171	0.01887	0.024647	0.213613	0.418849
Anaerovorax	0.0	0.0	0.007234	0.020911	0.001819	0.003865	0.0	0.0	0.229078	0.429521
Bradyrhizobium	0.002268	0.0051	0.001689	0.003561	0.0	0.0	0.0	0.0	0.229078	0.429521
Campylobacter	0.002005	0.004244	0.003002	0.004168	0.002945	0.00511	0.0	0.0	0.224114	0.429521
Comamonas	0.001333	0.002892	0.029634	0.091838	0.0	0.0	0.0	0.0	0.228562	0.429521
Dyadobacter	0.001143	0.002535	0.0	0.0	0.001262	0.002975	0.0	0.0	0.229078	0.429521
Lachnospiraceae_NK4B4	0.0	0.0	0.0	0.0	0.005456	0.011512	0.004046	0.008536	0.22702	0.429521
Peptococcus	0.0	0.0	0.001756	0.003779	0.002828	0.006608	0.0	0.0	0.228562	0.429521
Neisseria	0.01069	0.010964	0.024166	0.016536	0.026145	0.021798	0.015456	0.018726	0.232597	0.433411
Desulfovibrio	0.002606	0.00824	0.028167	0.060618	0.004564	0.009632	0.002295	0.007259	0.24287	0.449759
AKYG1722	0.0	0.0	3.31E-4	0.001046	0.0	0.0	0.0	0.0	0.391625	0.458936
AKYG587	0.0	0.0	8.62E-4	0.002726	0.0	0.0	0.0	0.0	0.391625	0.458936
Abiotrophia	0.0	0.0	0.0	0.0	0.0	0.0	5.74E-4	0.001815	0.391625	0.458936
Absconditabacteriales	7.22E-4	0.002283	0.0	0.0	0.0	0.0	0.0	0.0	0.391625	0.458936
Acidibacter	0.0	0.0	0.0	0.0	6.07E-4	0.00192	0.0	0.0	0.391625	0.458936
Acidothermus	0.0	0.0	0.0	0.0	0.0	0.0	5.74E-4	0.001815	0.391625	0.458936
Acidovorax	0.01333	0.014948	0.023742	0.021832	0.006907	0.010335	0.01192	0.013637	0.26879	0.458936

Acuticoccus	0.0	0.0	0.0	0.0	0.003471	0.010975	0.0	0.0	0.391625	0.458936
Adlercreutzia	0.0	0.0	0.002079	0.006573	0.0	0.0	0.002976	0.006335	0.292774	0.458936
Aggregatibacter	0.001624	0.005136	0.0	0.0	0.005837	0.012331	0.0	0.0	0.260901	0.458936
Agromyces	0.0	0.0	6.61E-4	0.002092	0.0	0.0	0.0	0.0	0.391625	0.458936
Alicyclobacillus	0.0	0.0	3.92E-4	0.00124	0.0	0.0	0.0	0.0	0.391625	0.458936
Altererythrobacter	0.0	0.0	6.61E-4	0.002092	0.0	0.0	0.0	0.0	0.391625	0.458936
Amycolatopsis	0.0	0.0	3.75E-4	0.001187	0.0	0.0	0.0	0.0	0.391625	0.458936
Bacillus	0.009669	0.020975	0.01384	0.015971	0.004986	0.015768	0.008274	0.018163	0.369853	0.458936
Bilophila	0.0	0.0	0.002074	0.004372	0.0	0.0	3.83E-4	0.00121	0.260901	0.458936
Brachybacterium	0.022349	0.044958	0.004191	0.009177	0.004144	0.008812	0.003387	0.007693	0.341167	0.458936
Butyricimonas	0.0	0.0	5.28E-4	0.001671	0.0	0.0	0.0	0.0	0.391625	0.458936
Capnocytophaga	0.001564	0.004944	0.00294	0.004776	0.001351	0.004273	0.0	0.0	0.294598	0.458936
Caulobacter	0.0	0.0	0.0	0.0	0.0	0.0	9.77E-4	0.00309	0.391625	0.458936
Centipeda	0.0	0.0	0.0	0.0	0.001279	0.004045	0.0	0.0	0.391625	0.458936
Cereibacter	0.0	0.0	0.0	0.0	0.0	0.0	0.001173	0.003708	0.391625	0.458936
Chitinophagaceae	0.0	0.0	4.96E-4	0.001569	0.0	0.0	0.0	0.0	0.391625	0.458936
Christensenellaceae	0.0	0.0	0.0	0.0	0.0	0.0	6.28E-4	0.001984	0.391625	0.458936
Chroococcidiopsis	7.22E-4	0.002283	0.0	0.0	0.0	0.0	0.0	0.0	0.391625	0.458936
Clostridium	0.106497	0.127392	0.050532	0.037971	0.121281	0.195495	0.141954	0.146866	0.360448	0.458936
Colidextribacter	0.007344	0.011972	0.052163	0.119969	0.012561	0.021973	0.013969	0.026762	0.369777	0.458936
Coprobacter	0.0	0.0	0.0	0.0	4.08E-4	0.001291	0.0	0.0	0.391625	0.458936
Dechloromonas	0.004771	0.010085	0.0	0.0	0.00251	0.007936	0.0	0.0	0.277221	0.458936
Defluviimonas	0.0	0.0	0.0	0.0	0.009799	0.030989	0.0	0.0	0.391625	0.458936
Delftia	0.0	0.0	0.006383	0.020183	0.0	0.0	0.00193	0.004183	0.292774	0.458936
Denitratisoma	0.002835	0.006375	0.0	0.0	6.06E-4	0.001915	0.0	0.0	0.260901	0.458936
Dojkabacteria	9.78E-4	0.003094	0.0	0.0	0.0	0.0	0.0	0.0	0.391625	0.458936
Dongia	0.0	0.0	0.001158	0.00366	0.0	0.0	0.0	0.0	0.391625	0.458936
Ensifer	0.0	0.0	6.9E-4	0.00218	0.0	0.0	0.0	0.0	0.391625	0.458936
Erysipelatoclostridium	0.003135	0.006614	0.005503	0.012174	0.023666	0.044969	0.0	0.0	0.309728	0.458936
Facklamia	0.0	0.0	8.81E-4	0.002785	0.0	0.0	0.0	0.0	0.391625	0.458936

Fournierella	0.0	0.0	6.24E-4	0.001972	0.00202	0.005125	0.0	0.0	0.277221	0.458936
Glutamicibacter	0.011405	0.029293	0.028678	0.05739	0.002015	0.006373	0.012333	0.01564	0.253559	0.458936
Haliangium	0.0	0.0	3.52E-4	0.001114	0.0	0.0	0.0	0.0	0.391625	0.458936
Herbaspirillum	0.0	0.0	0.0	0.0	5.47E-4	0.001731	0.0	0.0	0.391625	0.458936
Ideonella	0.0	0.0	0.001034	0.003271	0.0	0.0	0.0	0.0	0.391625	0.458936
Ileibacterium	9.78E-4	0.003094	0.0	0.0	0.0	0.0	0.0	0.0	0.391625	0.458936
Intestinibacter	0.0	0.0	8.81E-4	0.002785	0.0	0.0	0.0	0.0	0.391625	0.458936
JG30_KF_CM66	0.0	0.0	6.61E-4	0.002092	0.0	0.0	0.0	0.0	0.391625	0.458936
Janthinobacterium	0.0	0.0	0.011172	0.026713	0.0	0.0	0.00234	0.0074	0.260901	0.458936
Johnsonella	0.0	0.0	3.31E-4	0.001046	0.0	0.0	0.0	0.0	0.391625	0.458936
Lechevalieria	0.0	0.0	0.001409	0.004457	0.0	0.0	0.0	0.0	0.391625	0.458936
Leptolyngbya_EcFYyyy_00	0.0	0.0	0.0	0.0	0.0	0.0	4.18E-4	0.001323	0.391625	0.458936
Luteolibacter	0.0	0.0	3.75E-4	0.001187	0.0	0.0	0.0	0.0	0.391625	0.458936
Mesorhizobium	0.0	0.0	0.0	0.0	9.23E-4	0.002918	0.0	0.0	0.391625	0.458936
Methylobacterium_Methylorubrum	0.001624	0.005136	0.023415	0.067126	0.014719	0.035101	0.0	0.0	0.292431	0.458936
Mitsuokella	0.0	0.0	3.45E-4	0.00109	0.0	0.0	0.0	0.0	0.391625	0.458936
Morganella	0.0	0.0	0.001158	0.00366	0.0	0.0	0.0	0.0	0.391625	0.458936
Mucispirillum	0.005371	0.012481	0.018702	0.024945	0.006216	0.009972	0.005274	0.013095	0.286221	0.458936
Mycobacterium	0.0	0.0	0.001488	0.004706	0.0	0.0	0.0	0.0	0.391625	0.458936
Myxococcus	0.0	0.0	5.37E-4	0.001698	0.0	0.0	0.0	0.0	0.391625	0.458936
Nitrosospira	0.0	0.0	3.52E-4	0.001114	0.0	0.0	0.0	0.0	0.391625	0.458936
Nitrospira	0.0	0.0	0.002537	0.005373	0.0	0.0	4.18E-4	0.001323	0.260901	0.458936
Obscuribacteraceae	0.0	0.0	3.31E-4	0.001046	0.0	0.0	0.0	0.0	0.391625	0.458936
Oribacterium	7.55E-4	0.002388	0.0	0.0	0.0	0.0	0.0	0.0	0.391625	0.458936
Ottowia	0.0	0.0	0.004048	0.008886	0.0	0.0	0.001673	0.005292	0.277221	0.458936
PLTA13	0.0	0.0	0.0	0.0	8.08E-4	0.002554	0.0	0.0	0.391625	0.458936
Paeniclostridium	0.0	0.0	8.27E-4	0.002615	0.0	0.0	0.0	0.0	0.391625	0.458936
Paraprevotella	0.0	0.0	0.001247	0.003944	0.0	0.0	0.0	0.0	0.391625	0.458936
Parasegetibacter	0.0	0.0	8.62E-4	0.002726	0.0	0.0	0.0	0.0	0.391625	0.458936
Parvimonas	0.003367	0.007554	0.004552	0.007953	0.0	0.0	6.26E-4	0.001979	0.25735	0.458936

Pedomicrobium	0.0	0.0	3.31E-4	0.001046	0.0	0.0	0.0	0.0	0.391625	0.458936
Phaeodactylibacter	3.79E-4	0.001197	0.0	0.0	0.0	0.0	0.0	0.0	0.391625	0.458936
Planomicrobium	0.0	0.0	0.003004	0.009498	0.0	0.0	0.0	0.0	0.391625	0.458936
Pontibacter	0.0	0.0	6.61E-4	0.002092	0.0	0.0	0.0	0.0	0.391625	0.458936
Prevotella	0.015747	0.017375	0.021912	0.015923	0.019391	0.027242	0.031503	0.025749	0.391286	0.458936
Proteus	0.0	0.0	7.05E-4	0.002228	0.0	0.0	0.0	0.0	0.391625	0.458936
Pseudolabrys	5.41E-4	0.001712	0.0	0.0	0.0	0.0	0.0	0.0	0.391625	0.458936
Pseudoxanthomonas	0.0	0.0	0.0	0.0	0.0	0.0	4.18E-4	0.001323	0.391625	0.458936
Reyranella	0.0	0.0	6.9E-4	0.00218	0.0	0.0	0.0	0.0	0.391625	0.458936
Rheinheimera	0.05663	0.078137	0.027709	0.042807	0.030768	0.072878	0.005856	0.012195	0.3083	0.458936
Roseisolibacter	0.0	0.0	6.9E-4	0.00218	0.0	0.0	0.0	0.0	0.391625	0.458936
Ruminiclostridium	0.0	0.0	0.0	0.0	6.07E-4	0.00192	0.0	0.0	0.391625	0.458936
Saccharimonadaceae	0.0	0.0	0.0	0.0	0.0	0.0	7.65E-4	0.002419	0.391625	0.458936
Saccharimonadales	0.0	0.0	4.96E-4	0.001569	0.0	0.0	0.0	0.0	0.391625	0.458936
Segetibacter	0.0	0.0	3.52E-4	0.001114	0.0	0.0	0.0	0.0	0.391625	0.458936
Sellimonas	0.0	0.0	0.0	0.0	0.001214	0.003839	0.002775	0.005884	0.260901	0.458936
Shinella	0.0	0.0	0.0	0.0	0.0	0.0	0.001148	0.003629	0.391625	0.458936
Solirubrobacteraceae	0.0	0.0	5.28E-4	0.001671	0.0	0.0	0.0	0.0	0.391625	0.458936
Solobacterium	6.68E-4	0.002112	0.0	0.0	0.0	0.0	0.0	0.0	0.391625	0.458936
Spirosoma	0.0	0.0	0.0	0.0	0.001518	0.004799	0.0	0.0	0.391625	0.458936
Sporocytophaga	0.0	0.0	6.61E-4	0.002092	0.0	0.0	0.0	0.0	0.391625	0.458936
Stenotrophomonas	0.002606	0.00824	0.0	0.0	0.0	0.0	0.0	0.0	0.391625	0.458936
Terrimonas	0.0	0.0	4.96E-4	0.001569	0.0	0.0	0.0	0.0	0.391625	0.458936
Thauera	0.0	0.0	0.0	0.0	7.31E-4	0.002312	0.0	0.0	0.391625	0.458936
Truepera	0.0	0.0	4.16E-4	0.001315	0.0	0.0	0.0	0.0	0.391625	0.458936
Tyzzerella	0.0	0.0	5.82E-4	0.001842	0.0	0.0	0.0	0.0	0.391625	0.458936
Yimella	0.0	0.0	0.0	0.0	0.0	0.0	0.001046	0.003307	0.391625	0.458936
Oscillibacter	0.003252	0.006875	0.024891	0.0445	0.007069	0.010752	0.005566	0.012178	0.403501	0.471013
Peptostreptococcus	0.003971	0.006661	0.001034	0.003271	8.09E-4	0.002559	0.002802	0.005014	0.439307	0.510822
Candidatus_Soleaferrea	5.66E-4	0.001791	0.00157	0.003341	0.001617	0.003541	0.0	0.0	0.45259	0.520218

Holdemania	0.003651	0.008747	0.0	0.0	0.003031	0.006407	8.37E-4	0.002646	0.45259	0.520218
Selenomonas	0.002292	0.004967	3.45E-4	0.00109	0.0	0.0	0.002387	0.005695	0.45259	0.520218
Streptococcus	2.526464	1.316453	1.675032	0.860805	1.833013	1.21252	2.853633	2.293867	0.459975	0.526689
Prevotellaceae_NK3B31	0.065699	0.108177	0.41434	0.997378	0.134801	0.294019	0.371952	0.715717	0.495046	0.564691
Parabacteroides	0.01828	0.020142	0.03606	0.070439	0.024271	0.048663	0.059779	0.091397	0.507488	0.576691
Bdellovibrio	3.79E-4	0.001197	0.001225	0.002592	0.0	0.0	5.74E-4	0.001815	0.517023	0.583109
Bifidobacterium	0.810525	0.496389	1.320371	1.05137	0.836354	0.498964	2.965827	4.49352	0.515518	0.583109
Acholeplasma	3.78E-4	0.001194	0.0	0.0	0.0	0.0	5.86E-4	0.001854	0.561306	0.599259
Asticcacaulis	0.0	0.0	0.0	0.0	7.33E-4	0.002317	9.77E-4	0.00309	0.561306	0.599259
Bergeyella	0.0	0.0	3.92E-4	0.00124	0.0	0.0	8.37E-4	0.002646	0.561306	0.599259
Brevibacillus	0.00166	0.00525	0.0	0.0	0.0	0.0	7.82E-4	0.002472	0.561306	0.599259
Butyrivibrio	5.87E-4	0.001857	5.37E-4	0.001698	0.0	0.0	0.0	0.0	0.561306	0.599259
Craurococcus_Caldovatus	9.02E-4	0.002853	3.52E-4	0.001114	0.0	0.0	0.0	0.0	0.561306	0.599259
Defluviitaleaceae_UCG_011	0.0	0.0	6.9E-4	0.00218	0.0	0.0	6.28E-4	0.001984	0.561306	0.599259
Diaphorobacter	0.0	0.0	0.003138	0.009923	9.12E-4	0.002884	0.0	0.0	0.561306	0.599259
Elizabethkingia	7.55E-4	0.002388	5.28E-4	0.001671	0.0	0.0	0.0	0.0	0.561306	0.599259
Hymenobacter	0.0	0.0	5.17E-4	0.001635	0.005854	0.018511	0.0	0.0	0.561306	0.599259
Lachnospiraceae_NK4A136	0.039473	0.030541	0.454785	0.988476	0.088793	0.11858	0.125831	0.184241	0.546683	0.599259
Paludicola	0.0	0.0	0.0	0.0	6.07E-4	0.00192	6.28E-4	0.001984	0.561306	0.599259
Peredibacter	0.0	0.0	3.31E-4	0.001046	5.48E-4	0.001734	0.0	0.0	0.561306	0.599259
Roseomonas	9.22E-4	0.002917	8.62E-4	0.002726	0.0	0.0	0.0	0.0	0.561306	0.599259
Variovorax	0.0	0.0	0.002065	0.00653	0.005854	0.018511	0.0	0.0	0.561306	0.599259
Faecalibaculum	0.139157	0.159062	0.052407	0.034163	0.723972	1.259995	0.427553	0.780197	0.612073	0.648841
Gastranaerophilales	3.8E-4	0.001202	0.018128	0.040434	0.005054	0.011491	0.003395	0.007394	0.611166	0.648841
Massilia	0.003776	0.011942	0.01201	0.023067	0.004986	0.015768	0.003633	0.007983	0.628485	0.663893
Monoglobus	0.001686	0.003808	0.018128	0.035038	0.007699	0.02041	0.007938	0.014439	0.634431	0.667822
Turicibacter	0.033497	0.010295	0.063735	0.052739	0.053899	0.053163	0.056541	0.05537	0.645644	0.677249
Tuzzerella	9.78E-4	0.003094	0.010012	0.020579	0.007275	0.015452	0.005968	0.012585	0.700466	0.732194
Muribaculaceae	0.502108	0.613485	3.687718	7.578837	2.305189	3.929965	1.766368	2.961413	0.751952	0.783283
Aureimonas	0.0	0.0	0.001961	0.006202	0.006533	0.020659	0.001086	0.003433	0.786938	0.811276

Citrobacter	0.0	0.0	0.005241	0.016574	0.002602	0.008227	0.002871	0.009079	0.786938	0.811276
Filifactor	0.001174	0.003713	3.45E-4	0.00109	3.69E-4	0.001167	0.0	0.0	0.786938	0.811276
Treponema	0.001909	0.004381	5.17E-4	0.001635	0.001021	0.003228	0.00234	0.005661	0.81916	0.841603
Bryobacter	7.83E-4	0.001653	0.002716	0.00598	6.07E-4	0.00192	3.8E-4	0.001202	0.828769	0.848569
Helicobacter	0.016564	0.012074	0.084651	0.1835	0.021404	0.025474	0.016281	0.021852	0.849604	0.866943
Alkanindiges	9.46E-4	0.002993	0.004182	0.009444	0.001615	0.005107	0.002092	0.006615	0.857413	0.871945
Intestinimonas	0.002839	0.00638	0.036248	0.085149	0.012738	0.029791	0.008317	0.017556	0.867246	0.878966
JG30_KF_CM45	0.010848	0.018839	0.041287	0.086388	0.007731	0.015547	0.01226	0.028718	0.886534	0.895489
Enterorhabdus	9.78E-4	0.003094	0.00977	0.030896	0.002023	0.006398	0.002349	0.004988	0.896822	0.902841
Leptotrichia	0.002545	0.006378	0.004087	0.00749	0.001645	0.003922	0.001805	0.003862	0.908982	0.912022
Marvinbryantia	0.010005	0.016705	0.003823	0.009864	0.029918	0.064908	0.009501	0.022916	0.938629	0.938629
Candidatus_Nitrotoga	0.093551	0.021986	0.0	0.0	0.026756	0.043134	0.0	0.0	2.0E-6	1.5E-4
Nitrosomonas	0.020167	0.006688	0.0	0.0	0.005401	0.008744	0.0	0.0	2.0E-6	1.5E-4
Ruminococcus_torques	0.001805	0.005707	0.166413	0.263944	0.001601	0.00339	0.0	0.0	1.0E-6	1.5E-4
Dubosiella	0.002153	0.006807	0.049373	0.048413	0.001097	0.003467	0.0	0.0	5.0E-6	3.0E-4
Eubacterium_hallii	0.0	0.0	0.049758	0.059735	0.0	0.0	0.001141	0.003607	9.0E-6	3.38E-4
Lactobacillus	24.082938	12.60177	1.257475	1.266143	11.185858	6.224073	16.191434	6.3353	9.0E-6	3.38E-4
Veillonella	40.774876	9.832521	0.842188	0.509258	56.722053	15.953529	34.171698	14.331654	7.0E-6	3.38E-4
Agathobacter	0.005574	0.011505	0.245051	0.348268	0.002744	0.00617	0.002026	0.004417	1.4E-5	4.67E-4
Aeromonas	0.0	0.0	0.040069	0.029715	0.0	0.0	0.0	0.0	1.9E-5	4.75E-4
Finegoldia	0.0	0.0	0.0	0.0	0.0	0.0	0.009078	0.00666	1.9E-5	4.75E-4
Fusicatenibacter	0.011122	0.01396	0.22566	0.22632	0.00794	0.008814	0.003458	0.00618	1.9E-5	4.75E-4
Collinsella	0.016988	0.03737	0.422384	0.527632	0.010048	0.014018	0.004412	0.009308	2.4E-5	4.8E-4
Coriobacteriaceae_UCG_002	0.727185	0.448299	0.05501	0.060448	1.153734	0.740201	1.512674	0.962769	2.3E-5	4.8E-4
Enterococcus	0.563382	0.280254	0.106578	0.086028	0.442512	0.202213	0.293753	0.07924	2.4E-5	4.8E-4
Catenibacterium	0.004627	0.008463	0.075134	0.050846	0.003193	0.006517	0.001423	0.003382	2.7E-5	5.06E-4
Fusobacterium	1.477476	1.034263	0.055878	0.047475	1.028282	0.831386	2.577465	3.528167	4.6E-5	8.12E-4
**Species**										
Bifidobacterium_pseudolongum	0.752633	0.503219	0.083116	0.038644	0.798616	0.506708	2.919423	4.515095	6.5E-5	0.001155
Blautia_sp	0.001322	0.00418	0.055345	0.0696	0.0	0.0	0.0	0.0	7.6E-5	0.001208

Eubacterium_hallii	0.0	0.0	0.044221	0.058992	0.0	0.0	0.001141	0.003607	7.6E-5	0.001208
Rothia_mucilaginosa	0.0	0.0	0.025556	0.019074	0.0	0.0	0.002472	0.007816	9.1E-5	0.001309
Rothia_sp	1.208799	0.899389	11.194457	6.823622	0.675831	1.122837	1.069541	0.887561	9.0E-5	0.001309
Coprococcus_catus	9.44E-4	0.002986	0.023974	0.028971	0.0	0.0	0.0	0.0	1.09E-4	0.001431
Fusobacterium_nucleatum	0.0	0.0	0.002865	0.009059	0.0	0.0	0.026068	0.019771	1.09E-4	0.001431
Brevundimonas_intermedia	0.005328	0.013248	0.042987	0.03068	0.0	0.0	0.002678	0.008468	1.16E-4	0.00146
Pygmaiobacter_massiliensis	0.0	0.0	0.007422	0.010153	0.0	0.0	0.0	0.0	1.36E-4	0.001643
Klebsiella_pneumoniae	0.017533	0.033722	0.112114	0.07723	0.005639	0.012278	0.028349	0.018114	1.49E-4	0.001689
Lactobacillus_reuteri	0.998803	1.142105	0.029228	0.018614	0.877367	1.268885	0.767466	1.337952	1.51E-4	0.001689
Enterococcus_gallinarum	0.060924	0.048492	0.0	0.0	0.010151	0.032101	0.0	0.0	1.82E-4	0.001895
Streptococcus_salivarius	0.0	0.0	0.059719	0.049648	0.0	0.0	0.012063	0.011515	1.8E-4	0.001895
Enterococcus_faecalis	0.14925	0.097556	0.003021	0.006386	0.066878	0.070069	0.040832	0.043337	2.07E-4	0.002084
Fusobacterium_varium	0.0	0.0	0.0	0.0	0.027338	0.020904	0.005526	0.017476	2.15E-4	0.002095
Faecalibacterium_prausnitzii	0.02795	0.039035	0.283749	0.290134	0.027164	0.031945	0.023513	0.032436	3.53E-4	0.003249
Paenibacillus_odorifer	0.045668	0.097701	1.799892	4.590146	0.017514	0.028749	0.045629	0.046832	3.55E-4	0.003249
Clostridium_perfringens	0.010916	0.014266	0.01258	0.010083	0.0	0.0	0.040749	0.024439	3.98E-4	0.003535
Alistipes_putredinis	7.55E-4	0.002388	0.014793	0.013518	0.0	0.0	0.002097	0.004442	4.9E-4	0.004228
Agrobacterium_radiobacter	0.013187	0.029498	0.085433	0.06691	0.005084	0.013303	0.060015	0.054501	5.89E-4	0.004941
Bifidobacterium_bifidum	0.0	0.0	0.056451	0.101565	0.0	0.0	0.0	0.0	8.46E-4	0.006387
Clostridium_baratii	0.017959	0.020427	0.0	0.0	0.0	0.0	0.0	0.0	8.46E-4	0.006387
Dialister_pneumosintes	0.035235	0.037331	0.0	0.0	0.0	0.0	0.0	0.0	8.46E-4	0.006387
Pseudomonas_otitidis	0.0	0.0	0.026785	0.029624	0.0	0.0	0.0	0.0	8.46E-4	0.006387
Burkholderia_cenocepacia	0.007867	0.008815	0.040449	0.021364	0.020855	0.016327	0.02663	0.011624	0.001111	0.008183
